# Synthesis and application of [Zr-UiO-66-PDC-SO_3_H]Cl MOFs to the preparation of dicyanomethylene pyridines via chemical and electrochemical methods

**DOI:** 10.1038/s41598-021-96001-7

**Published:** 2021-08-19

**Authors:** Amir Mohammad Naseri, Mahmoud Zarei, Saber Alizadeh, Saeed Babaee, Mohammad Ali Zolfigol, Davood Nematollahi, Jalal Arjomandi, Hu Shi

**Affiliations:** 1 Faculty of Chemistry, Bu-Ali-Sina University, 65174-38683 Hamedan, Iran; 2 School of Chemistry and Chemical Engineering, Institute of Molecular Science, Shanxi University, Taiyuan, 030006 China

**Keywords:** Electrochemistry, Green chemistry, Organic chemistry, Chemical synthesis

## Abstract

A metal–organic framework (MOF) with sulfonic acid tags as a novel mesoporous catalyst was synthesized. The precursor of Zr-UiO-66-PDC was synthesized both via chemical and electrochemical methods. Then, zirconium-based mesoporous metal–organic framework [Zr-UiO-66-PDC-SO_3_H]Cl was prepared by reaction of Zr-UiO-66-PDC and SO_3_HCl. The structure of [Zr-UiO-66-PDC-SO_3_H]Cl was confirmed by FT-IR, PXRD, FE-SEM, TEM, BET, EDX, and Mapping analysis. This mesoporous [Zr-UiO-66-PDC-SO_3_H]Cl was successfully applied for the synthesis of dicyanomethylene pyridine derivatives via condensation of various aldehyde, 2-aminoprop-1-ene-1,1,3-tricarbonitrile and malononitrile. At the electrochemical section, a green electrochemical method has successfully employed for rapid synthesis of the zirconium-based mesoporous metal–organic framework UiO-66-PDC at room temperature and atmospheric pressure. The synthesized UiO-66-PDC has a uniform cauliflower-like structure with a 13.5 nm mean pore diameter and 1081.6 m^2^ g^−1^ surface area. The described catalyst [Zr-UiO-66-PDC-SO_3_H]Cl was also employed for the convergent paired electrochemical synthesis of dihydropyridine derivatives as an environmentally friendly technique under constant current at 1.0 mA cm^−2^ in an undivided cell. The proposed method proceeds with moderate to good yields for the model via a cooperative vinylogous anomeric based oxidation.

## Introduction

Functionalized metal–organic frameworks (MOFs) are crystalline structures composed of suitable organic ligands and metal centers^1^. So far, these materials have been applied such as catalyst^2^, adsorbents and so on^3,4^. Ionic liquids-like MOFs (IL@MOFs) as novel homolog porous materials are some useful properties such as nonflammability, high thermal and chemical stability. Therefore, the joining of ionic liquids (ILs) and metal–organic frameworks (MOFs) as novel materials have been used as catalysts, gas adsorption and reagents^5,6^. This strategy have been modified to adjust to physical or chemical properties, pore size, surface area, topology, and polarity of IL@MOFs by suitable choice of metal, ligands and other moieties.

Zirconium is widespread in nature and used in biological systems. Firstly Zr-based metal–organic frameworks (Zr-MOFs) by Lillerud et al. in 2008 was prepared and reported^7^. Zr-MOFs has higher thermal stability and outstanding chemical stability in solvent and air^8^ than other M-MOFs (M = metal) which made them applicable for industrial process and organic synthesis. Topology or morphology of Zr-MOFs are such as Fcu, Csq, Ftw, Bct, Spn, Sqc and etc.^9–12^. Topology of Zr-UiO-66-PDC as one of the precursors for the preparation of the presented [Zr-UiO-66-PDC-SO_3_H]Cl have been studied^13^.

Ionic liquids with N–S bonds have been introduced by Zolfigol et al*.* in 2011^14^. These materials have been applied as catalysts, reagents and solvents in the synthesis of a wide range of organic compounds^15,16^. In 2013, 1-sulfopyridinium chloride [pyridine-SO_3_H]Cl has been synthesized by reaction of chlorosulfonic acid and pyridine (1:1) at 0 °C, which have been applied for the synthesis of other ILs with other anions via anion exchange methods^15–18^. To combined metal–organic frameworks (MOFs) and ionic liquids (ILs), we have reacted Zr-UiO-66-PDC and ClSO_3_H for preparing [Zr-UiO-66-PDC-SO_3_H]Cl as a novel porous catalyst.

In recent years, many efforts have been made to investigate the biological properties pyridine and its derivatives. Therefore, it is very important to provide novel and easy strategies for the synthesis of target molecules with specific properties. In this regard, pyridines and 1,4-dihydropyridines are suitable candidates for biological and pharmacological studies^19,20^. These compounds have been applied as drugs for cancer, malaria, HIV, antimicrobial, anti-tumour, antifungal, anticonvulsant, antihypertension and urinary incontinence treatment^19–27^.

**Figure 1:**
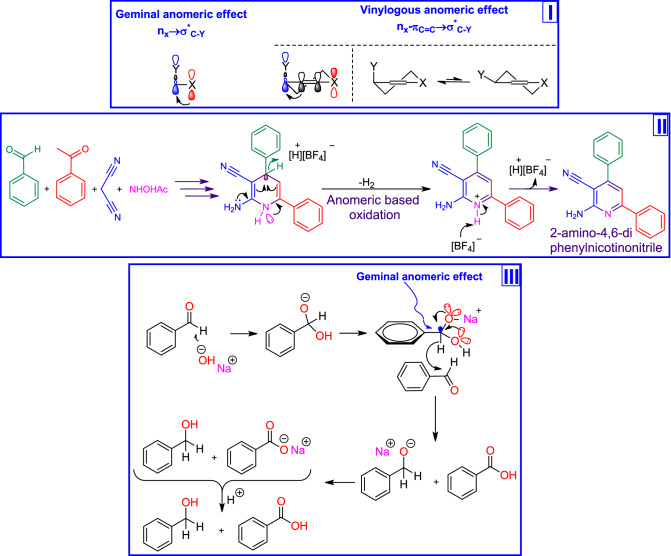
Part I: Geminal versus vinylogous anomeric effect. Part II: A cooperative vinylogous anomeric based oxidation leads to the preparation of 2-amino-4,6-diphenylnicotinonitrile^37^. Part III: A cooperative geminal anomeric based oxidation leads to hydride transfer in the mechanism of Cannizzaro reaction^38^ (CambridgeSoft).

On the other hand, chemical reactivity is very complex. Numerous factors control the reaction mechanisms which are subject to everyday experiences. According to the alabugin’s theory, one of the most effective factors is stereoelectronic effects, which are the stabilizing interactions of orbitals in space, are based on the quantum nature of molecular bonding but express this nature in a set of simple and intuitive practical rules that build a bridge between structure and reactivity^28,29^. Anomeric effect (AE) has been divided to different kinds such as geminal (Endo, Exo and reverse), vinylogous and so on (Fig. 1, Part I)^28^.

The relationship and detail of the impact of AE as an oldest stereoelectronic effect on structure and reactivity is also our main interest. This paper attempts to describe the role of vinylogous AE in the course of the synthesis of target molecules. Recently, we have introduced and developed a new term entitled “anomeric based oxidation” (ABO) in the course of special reactions^30–36^. Cooperative geminal and vinylogous ABO has been reviewed (Fig. 1, Parts II and III)^37,38^. Behind the chemical studies, many efforts have been done to access a mild and green condition for the electrosynthesis and application of MOFs^39–50^. Anodic and cathodic electrosynthesis methods are the important techniques that recently have been used for the preparation of various kinds of MOFs^39–44^. Even though the significant chemical^51–53^ and electrochemical synthesis^54–56^ of Zr based MOFs have been dedicated to the UiO-66 MOF, there are not any reports using cathodic electrosynthesis of UiO-66 derivatives at room temperature and pressure.

In this work, we wish to report the electrosynthesis of the mesoporous UiO-66-PDC (Zr-mMOF) via a reductive electrosynthesis technique as the first example. It was found that the electrosynthesis of Zr-mMOF by this method is rapid and could be done at room temperature and pressure without the need for any base or pre-base additive for activation of the ligand.

These results proved by the Fourier Transforms Infrared (FT-IR) spectroscopy, Field Emission Scanning Electron Microscopy (FE-SEM) and N_2_ adsorption–desorption isotherm. At the second step and after treatment of electro-synthesized UiO-66-PDC by the SO_3_HCl, the catalyst, [Zr-UiO-66-PDC-SO_3_H]Cl was employed in a green procedure for convergent paired electrosynthesis of dihydropyridine compounds. “Paired electrosynthesis” have been successfully employed for the synthesis of organic and inorganic compounds^40,57–59^. This positive glance comes from the improved energy efficiency, enhanced atom economy, time-saving, and increasing electrochemical yield^60–63^. We imagined that the convergent pairing of two electrochemical reactions would provide a promising protocol towards the green chemistry principles. In other words, by the implementation of this strategy, cooperative anodic and cathodic reactions lead to a one-step process at green solvent and room temperature and without the need for any ex-situ base additive and replacement of the electrodes.

**Figure 2:**
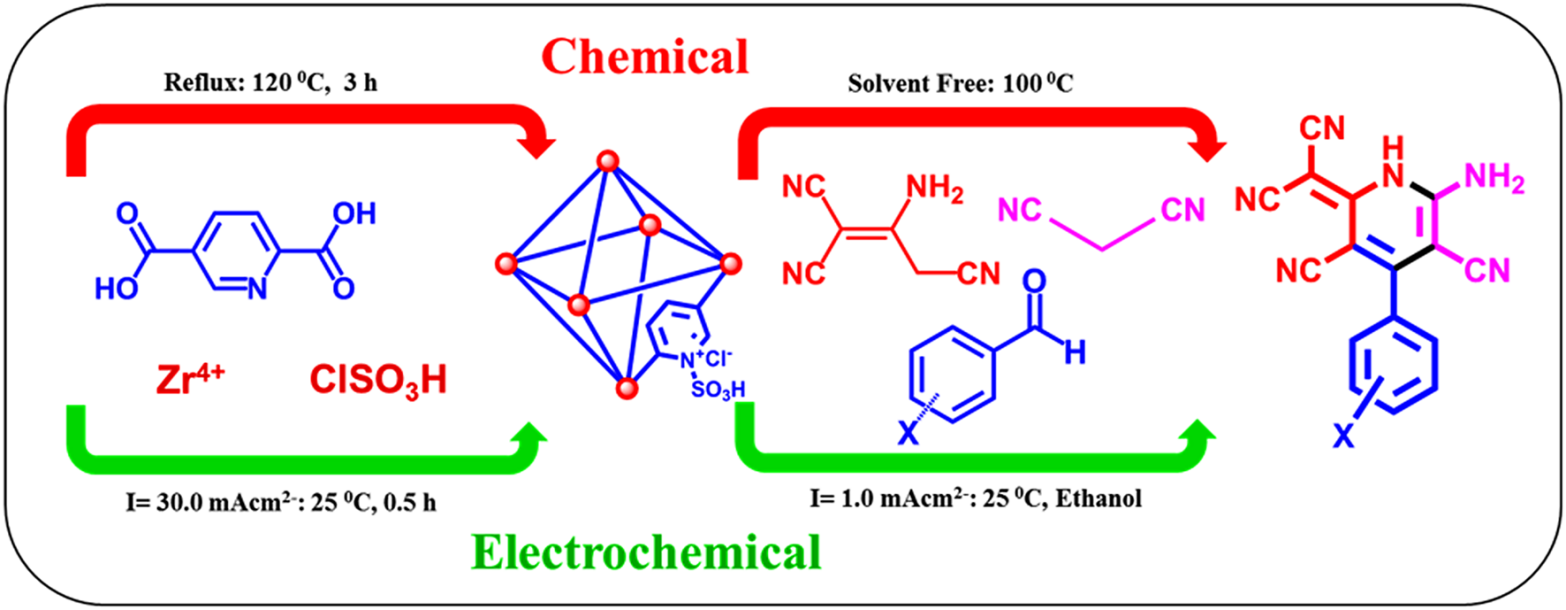
Synthesis of dicyanomethylene pyridines using [Zr-UiO-66-PDC-SO_3_H]Cl as a catalyst by chemical (A) and electrochemical (B) methods.

According to the above concepts, after preparation of Zr-metal–organic frameworks [Zr-UiO-66-PDC-SO_3_H]Cl as a mesoporous catalyst, it was employed for the synthesis and electrosynthesis of special dicyanomethylene pyridines by condensation of various aldehydes (bearing electron-donating and electron-withdrawing groups), malononitrile and 2-aminoprop-1-ene-1,1,3-tricarbonitrile under solvent-free conditions at 100 °C (method A) and constant current electrolysis via the convergent paired electrosynthesis in the ethanol at room temperature and pressure (method B) (Fig. 2).

## Results and discussion

Nowadays inter and multidisciplinary researches and investigations are a great demand both for academics and industries researchers. On the other hand, making bridges between basic to advanced concepts are necessary for the development of knowledge. In this research, chemical and electrochemical methods for the preparation of designed molecules were applied. Stereoelectronic effects as a bridge between structure and reactivity was also considered in the course of reactions^28^. With this aim, we have studied reactions and the obtained results are presented.

### Chemical and electrochemical preparation of Zr-mMOF

Metal–organic frameworks (MOFs) based on Zr is attractive groups for preparing catalysts and/or reagents. Herein, we attempt to the preparation of Zr-UiO-66-PDC with sulfonic acid groups. For this purpose, we prepared Zr-UiO-66-PDC via H_2_PDC and ZrCl_4_^13^. Then, [Zr-UiO-66-PDC-SO_3_H]Cl was prepared as ionic liquid transported into Zr-UiO-66-PDC. The structure of mentioned ionic liquid transported into Zr-MOFs, [Zr-UiO-66-PDC-SO_3_H]Cl, fully was characterized by applying FT-IR, XRD, BET/BJH, TG, DTG, EDX, FE-SEM as well as TEM analysis.

**Figure 3:**
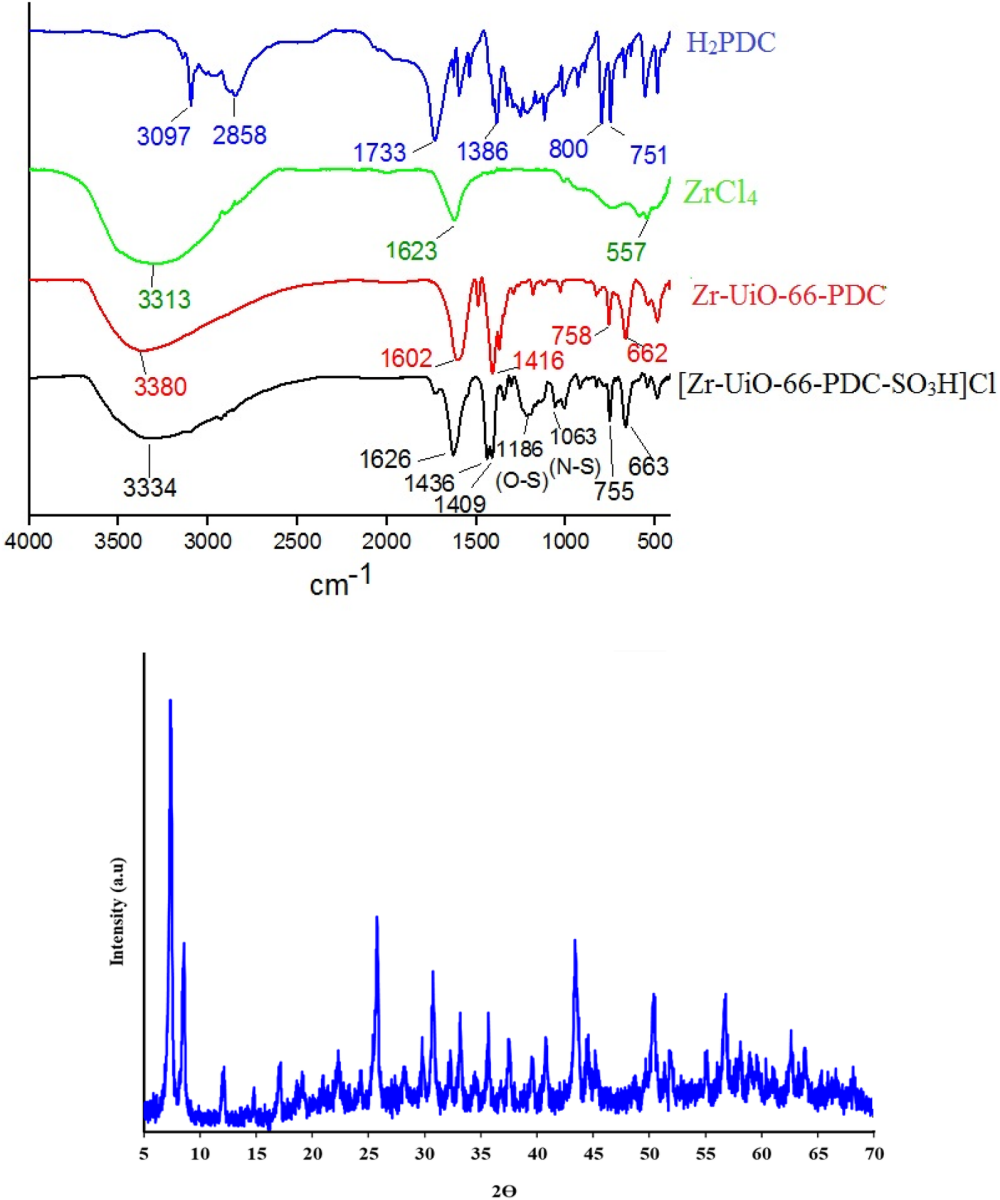
Up: FT-IR spectra of pyridine-2,5-dicarboxylic acid (H_2_PDC), ZrCl_4_, Zr-UiO-66-PDC and [Zr-UiO-66-PDC-SO_3_H]Cl. Down: PXRD pattern of the [Zr-UiO-66-PDC-SO_3_H]Cl.

The FT-IR spectrum of pyridine-2,5-dicarboxylic acid (H_2_PDC), ZrCl_4_, Zr-UiO-66-PDC and [Zr-UiO-66-PDC-SO_3_H]Cl were compared in Fig. 3. The broad peak of O–H stretching related to SO_3_H group at 2700–3500 cm^−1^ and peaks observed at 1186 and 1063 cm^−1^ were related to stretching O-S and N-S respectively^16^. The peak 1733 cm^−1^ in H_2_PDC was related to stretching C = O bond. Also, the PXRD pattern indicates the crystallinity of synthesized [Zr-UiO-66-PDC-SO_3_H]Cl (Fig. 3)^13^.

**Figure 4:**
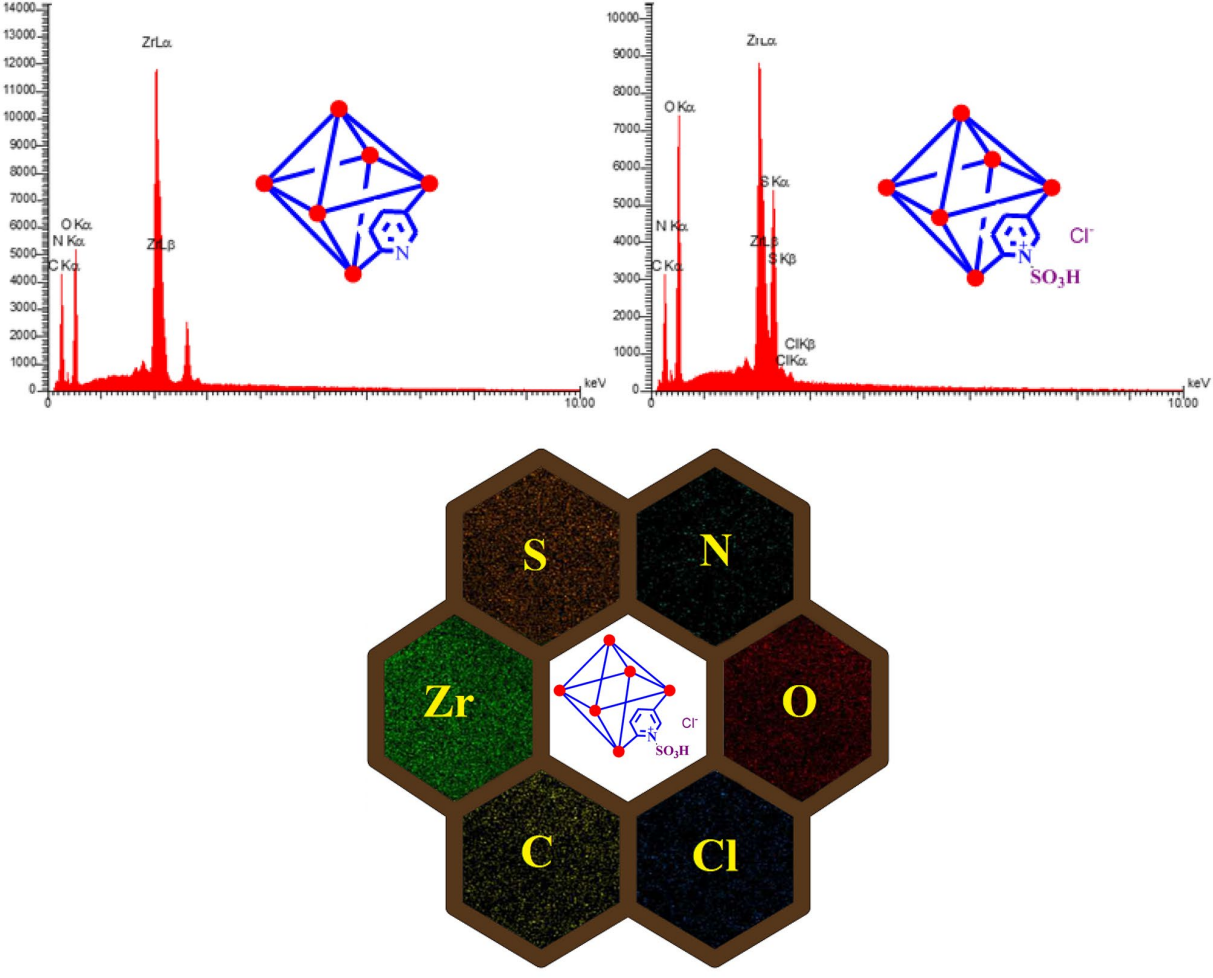
Up: Energy-dispersive X-ray spectroscopy (EDX) of [Zr-UiO-66-PDC-SO_3_H]Cl and Zr-UiO-66-PDC. Down: Elemental mapping analysis of [Zr-UiO-66-PDC-SO_3_H]Cl. The structures of the compounds were drawn using Chem Office 12.0 (Cambridge Soft).

The materials in the structure of [Zr-UiO-66-PDC-SO_3_H]Cl and Zr-UiO-66-PDC were characterized by energy-dispersive X-ray spectroscopy (EDX) (Fig. 4). The [Zr-UiO-66-PDC-SO_3_H]Cl has confirmed the existence of Zr, C, O, S, Cl and N atoms whereas the structure of Zr-UiO-66-PDC which is contained Zr, C, N and O atoms. Furthermore, the well-dispersed distribution of elements in the [Zr-UiO-66-PDC-SO_3_H]Cl was determined and verified by elemental mapping (Fig. 4). The difference between FT-IR, EDX and SEM-elemental mapping of Zr-UiO-66-PDC and [Zr-UiO-66-PDC-SO_3_H]Cl vouched for the structure of target Zr-MOFs.

**Figure 5:**
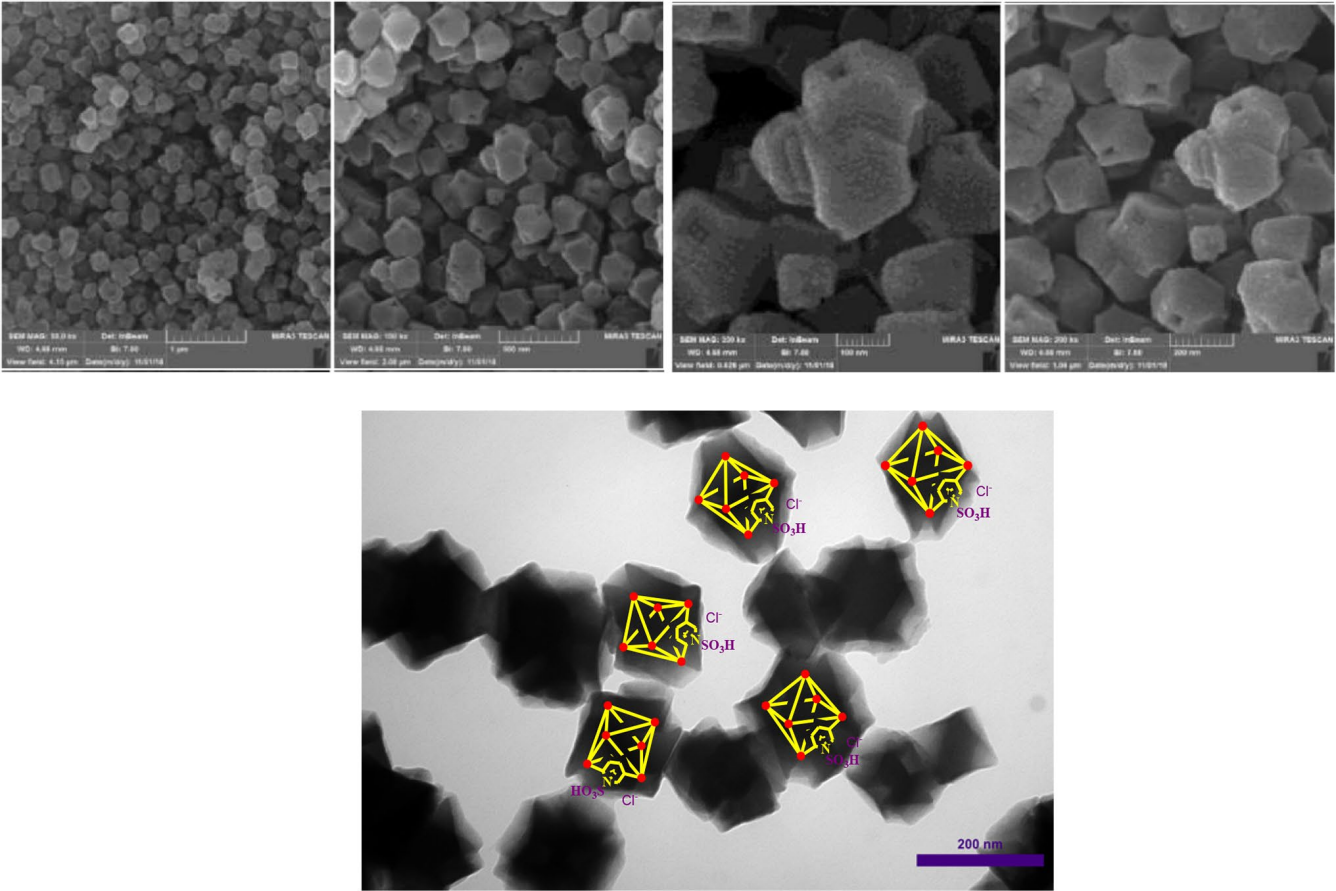
Up: Field-Emission Scanning electron microscopy (FE-SEM) images of [Zr-UiO-66-PDC-SO_3_H]Cl. Down: Transmission electron microscopy (TEM) micrograph of [Zr-UiO-66-PDC-SO_3_H]Cl.

In another investigation, the topography of [Zr-UiO-66-PDC-SO_3_H]Cl was examined by scanning electron microscopy (SEM) images. As shown in Fig. 5, topography particles of the catalysts are Fcu which are in good agreement and not completely accumulated. In addition, the topography structure of [Zr-UiO-66-PDC-SO_3_H]Cl was studied more closely using transmission electron microscopy (TEM) micrograph in Fig. 5. Therefore, [Zr-UiO-66-PDC-SO_3_H]Cl are fcu topological network with 12-connected Zr clusters.

**Figure 6:**
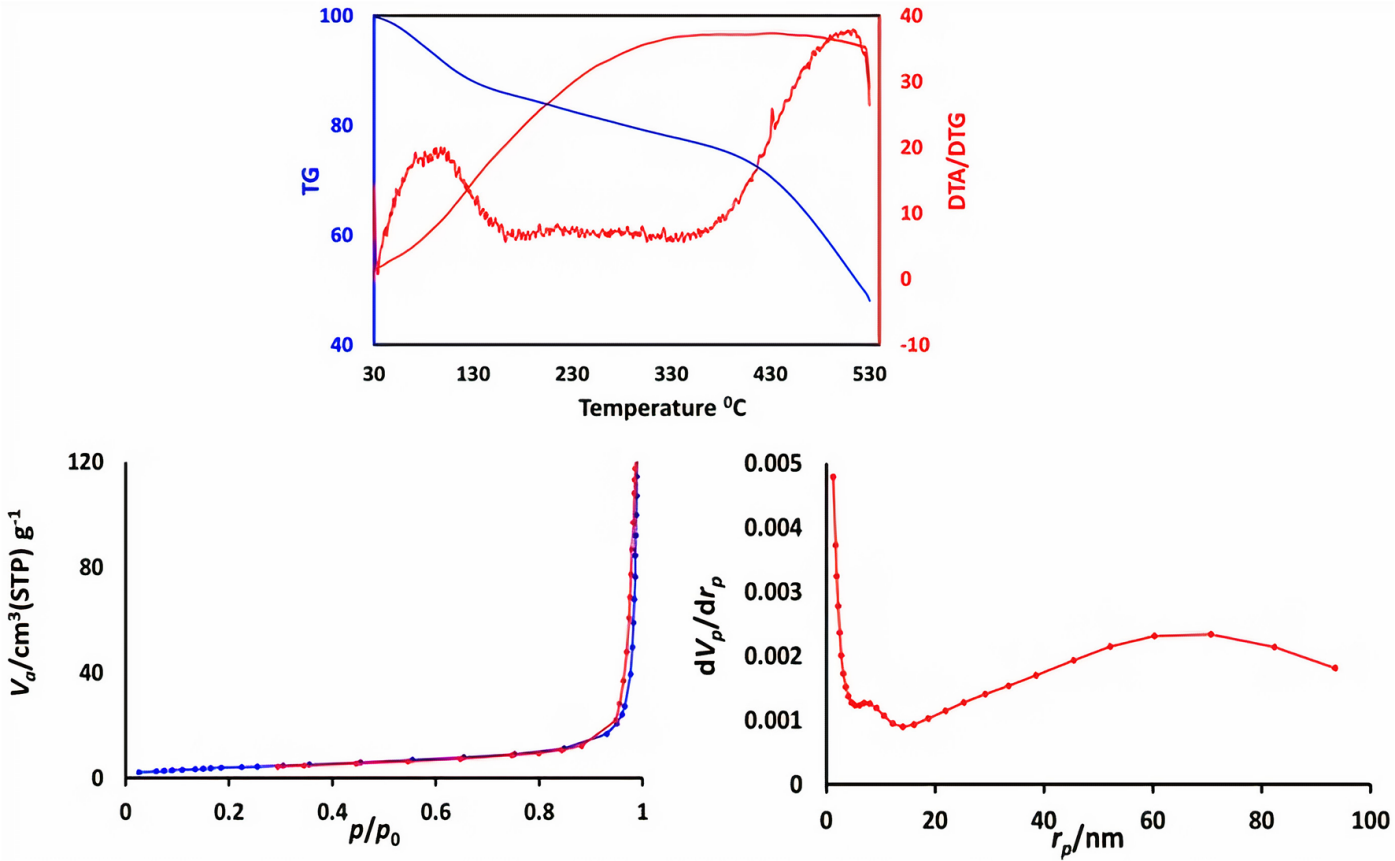
Up: TG, DTG and DTA analysis of [Zr-UiO-66-PDC-SO_3_H]Cl. This figure was prepared by Microsoft Excel (OFFICE 2013). Down: N_2_ adsorption/desorption isotherm and pore size distribution (BJH) of [Zr-UiO-66-PDC-SO_3_H]Cl. This figure was prepared by Microsoft Excel (OFFICE 2013).

In order investigation, the structural and thermal stability of [Zr-UiO-66-PDC-SO_3_H]Cl was also determined using the technique of the thermal gravimetric (TG), derivative thermal gravimetric (DTG), as well as differential thermal analysis (DTA) (Fig. 6). Initial stage weight loss is between room temperature up to 100 °C, associated with organic solvents and H_2_O which have been applied in the course of preparation of [Zr-UiO-66-PDC-SO_3_H]Cl. In continued, twice steps of weight loss (includes about 30% weight loss) has occurred at about 300 °C which is linked to breaking the band of N-S of the structure of the catalyst. Therefore, according to literature survey^13^, the structure of [Zr-UiO-66-PDC-SO_3_H]Cl is stable, even after adding sulfonic acidic functional groups.

Features of the prepared functionalized MOFs such as Surface area, pore volumes and pore size distribution were obtained using *N*_*2*_ adsorption–desorption isotherm (Fig. 6). The calculated surface areas using the BET equation, total pore volume and average pore size are 15.24 m^2^ g^−1^, 0.1914 cm^3^ g^−1^ and 50.22 nm respectively.

**Figure 7:**
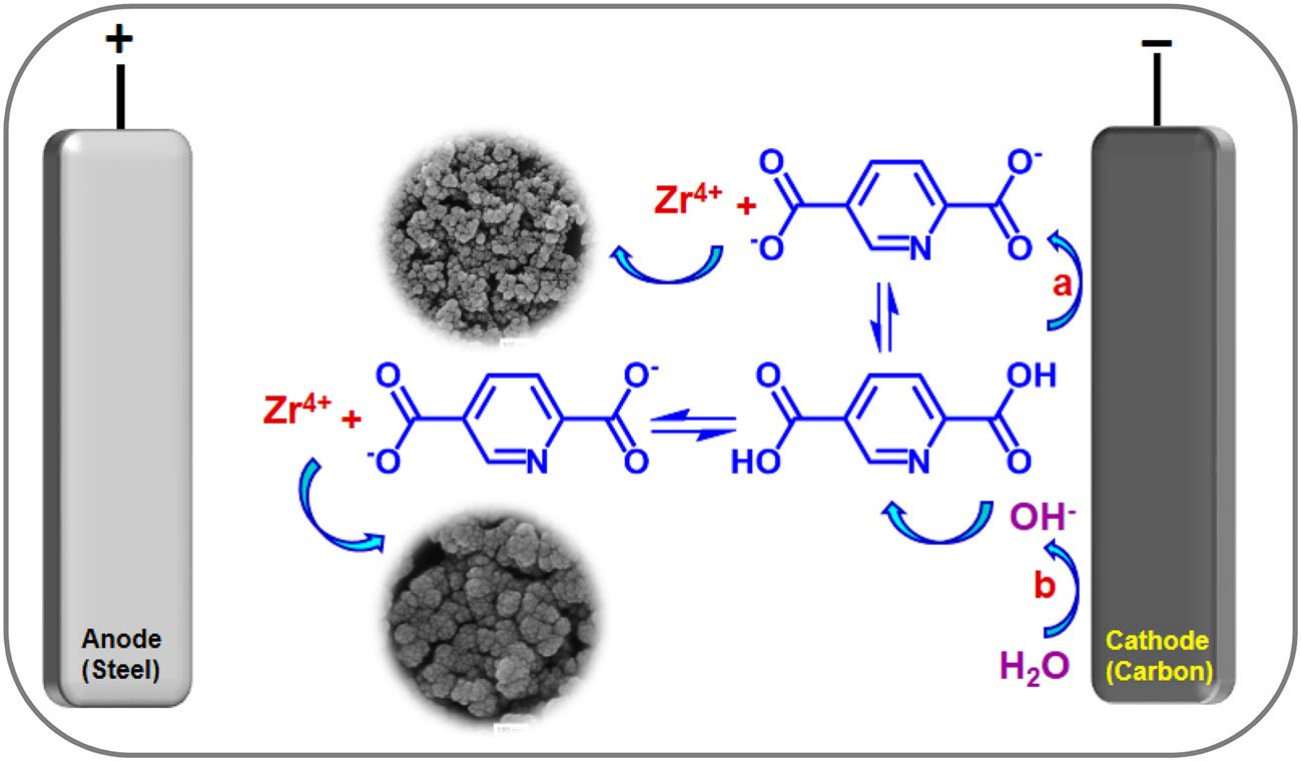
Electrochemical synthesis of (UiO-66-PDC) by constant current electrolysis at *I* = 30 mA cm^−2^ and t = 1800s.

We also employed a cathodic (reductive) electrochemical technique for the preparation of Zr-UiO-66-PDC. As shown in Fig. 7. Our procedure involves immersing a carbon electrode in a solution containing pyridine-2,5-dicarboxylic acid (H_2_PDC) as a ligand, zirconium tetrachloride as a cation source and potassium nitrate as a supporting electrolyte. In-situ electrogeneration of hydroxide ions generated by electroreduction of water (as a co-solvent), NO_3_^−^ (as a counter ion) and/or direct deprotonation of ligand at 30.0 mA cm^−2^ for 1800s is an essential requirement in this method^39–42,44^. An increase in the local pH at the cathode surface causes activation of the ligands (deprotonation), and consequently formation of Zr-UiO-66-PDC through the coordination of activated ligands with zirconium cations (Fig. 7)^55,56^. The nucleation rate and growth of Zr-UiO-66-PDC are only controlled by the cathodic reaction without the need for any ex-situ base/probes additive, at room temperature, atmospheric pressure, and short time.

**Figure 8:**
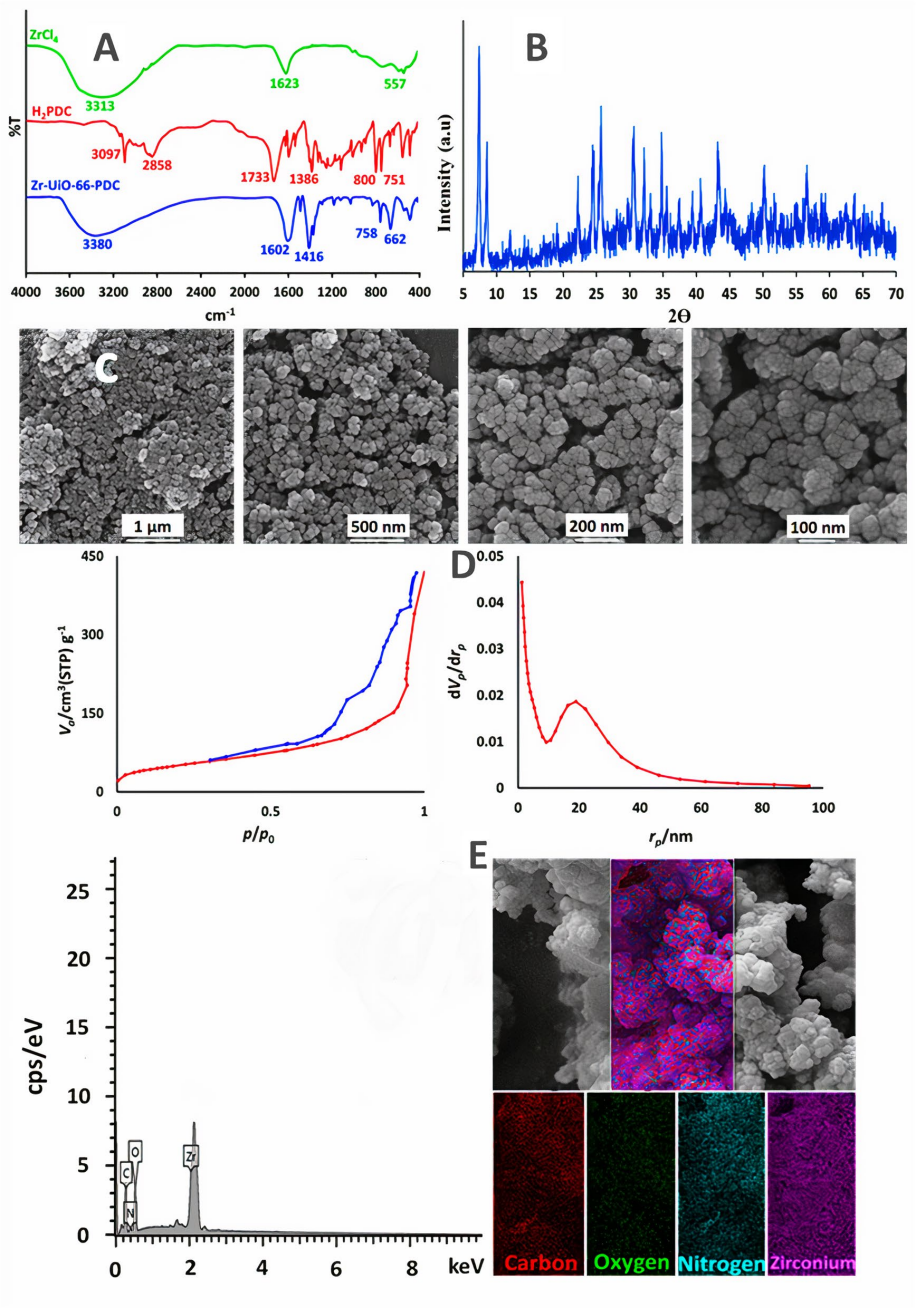
(**A**) FT-IR spectra of pyridine-2,5-dicarboxylic acid (H_2_PDC), ZrCl_4_ and Zr-UiO-66-PDC. (**B**) Powder X-ray diffraction (PXRD) pattern of electrosynthesized Zr-UiO-66-PDC. (**C**) Large- and close-view FE-SEM images of Zr-UiO-66-PDC by constant current electrolysis at *I* = 30 mA cm^−2^ and t = 1800s. (**D**) N_2_ adsorption/desorption isotherm. BET and BJH of Zr-UiO-66-PDC by the constant current electrolysis at *I* = 30 mA cm^−2^ and t = 1800s. (**E**) Energy-dispersive X-ray spectroscopy (EDX) and elemental mapping analysis of electrosynthesized Zr-UiO-66-PDC. The structures of the compounds were drawn using ChemOffice 12.0 (CambridgeSoft).

Characterization of the prepared Zr-UiO-66-PDC was examined by the FT-IR, PXRD, FE-SEM, BET, EDX and mapping analysis. To confirm the functionality and bonding groups of the electrosynthesized UiO-66-PDC MOF, FT-IR analysis was performed. The obtained pattern is consistent with the reported pattern of chemical procedure^13^ (Fig. 8A). Also, the recorded PXRD pattern proved the purity and high crystallinity of the electrosynthesized Zr-UiO-66-PDC MOF that is consistent with the reported pattern^13^ and the Zr based MOFs synthesized by electrochemical method^55,56^ (Fig. 8B). FE-SEM images of electrosynthesized (UiO-66-PDC) under constant current conditions shows the uniform cauliflower-shaped nanoparticles with an average diameter size of around 25.0 nm (Fig. 8C). This result is consistent with the Zr based MOFs synthesized by electrochemical method^55,56^. The N_2_ adsorption/desorption isotherm of Zr-UiO-66-PDC is shown in Fig. 8D. A “type IV” isotherm with a hysteresis loop (between p/p_0_ = 0.4 and 1) which is characteristic of mesoporous materials is observed.

Furthermore, the pore size distribution obtained by the Barrett-Joyner-Halenda (BJH) method shows two peaks of 1.2 and 18.9 nm but the average pore size is 13.4 nm. Besides, the specific surface area measured from the N_2_ isotherms is 1081.6 m^2^ g^−1^ that is higher than the obtained amount of above mentioned hydrothermal and microwave methods and is consistent with the Zr based MOFs prepared electrochemically^55,56^. Finally, the Zr-UiO-66-PDC structure was characterized by energy-dispersive X-ray spectroscopy (EDX) and mapping analysis (Fig. 8E). The obtained EDX pattern was confirmed the simultaneous existence of Zr, C, O, and N elements for the configuration of the Zr-UiO-66-PDC structure. Furthermore, mapping analysis was indicated the homogeneous and well-dispersed distribution of the above-mentioned elements in the Zr-UiO-66-PDC structure (Fig. 8E).

### Chemical and electrochemical synthesis of dicyanomethylene pyridine derivatives

Lately, a wide range of dicyanomethylene pyridines was prepared via one-pot multi-component condensation reactions in the presence of a catalytic amount described [Zr-UiO-66-PDC-SO_3_H]Cl as a mesoporous catalyst. The condensation of 4-chloro benzaldehyde, 2-aminoprop-1-ene-1,1,3-tricarbonitrile and malononitrile was selected as a model for optimization of the reaction conditions. As shown, the best condition reaction for the synthesis of 3,5-diaminobiphenyl-2,4,6-tricarbonitrile was achieved in the presence of 10.0 mg [Zr-UiO-66-PDC-SO_3_H]Cl in refluxing water (Table 1 entry 10). Different amount of catalyst, temperature and solvent were not improved in the yield and time (Table 1 entries 1–17 except 10). The optimization of reaction conditions along with the isolated yields of products are summarized in Table 1.

**Table 1:** Effect of different amounts of catalyst, temperature and solvent (5.0 mL) in the synthesis dicyanomethylene pyridine.


Entry	Solvent (5 mL)	Cat. (mg)	Temp. (˚C)	Time (min)	Isolated yield (%)
1	DMF	10	100	120	38
2	EtOH	10	Reflux	120	60
3	CH_2_Cl_2_	10	Reflux	120	63
4	CHCl_3_	10	Reflux	120	20
5	EtOAc	10	Reflux	120	40
6	CH_3_CN	10	Reflux	120	58
7	PEG	10	Reflux	120	35
8	*n*-Hexane	10	Reflux	120	65
9	H_2_O	10	Reflux	120	52
10	–	10	100	20	87
11	–	5	100	30	75
12	–	15	100	30	87
13	–	20	100	30	85
14	–	10	25	120	15
15	–	10	50	120	68
16	–	–	75	120	10
17	–	10	100	120	70

After optimization reaction condition, the scope and limitations of [Zr-UiO-66-PDC-SO_3_H]Cl as a novel catalyst was investigated in the preparation of dicyanomethylene pyridine via a condensation reaction of widespread analogue of aldehyde (mono, bis and tris substituted C=O) which are bearing electron-donating and electron-withdrawing groups, malononitrile and 2-aminoprop-1-ene-1,1,3-tricarbonitrile.

**Figure 9:**
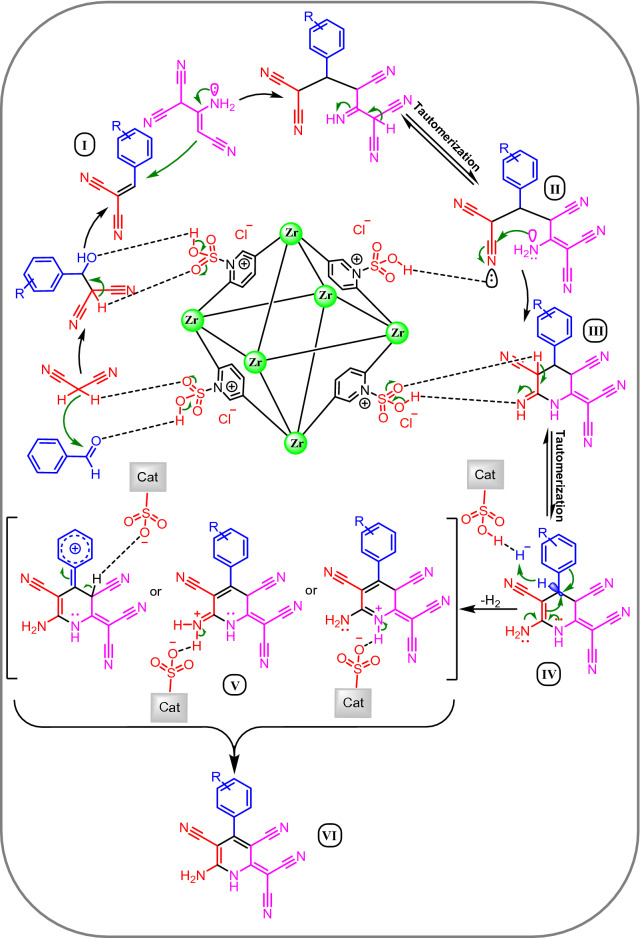
A rational proposed mechanism for the synthesis of dicyanomethylene pyridine using [Zr-UiO-66-PDC-SO_3_H]Cl.

As shown in Table 2, the results indicated that this strategy is appropriate for the synthesis of dicyanomethylene pyridine (Table 2). The proposed mechanism for the synthesis of dicyanomethylene pyridine derivatives using [Zr-UiO-66-PDC-SO_3_H]Cl was summarized in Fig. 9. The SO_3_H group of [Zr-UiO-66-PDC-SO_3_H]Cl is activating the carbonyl group of aldehyde. In the first step, malononitrile is reacting with the carbonyl group of aldehyde to afford Knoevenagel’s adduct I by removing one molecule of H_2_O. Then, 2-aminoprop-1-ene-1,1,3-tricarbonitrile attacks to I to give intermediate II, which is reacting to give III via an intramolecular cyclocondensation. Finally, the intermediate III and IV was converted to the desired product VI through intermediate V via a cooperative vinylogous ABO and releasing one molecule of hydrogen (H_2_) (Fig. 9)^31^.

**Table 2:**
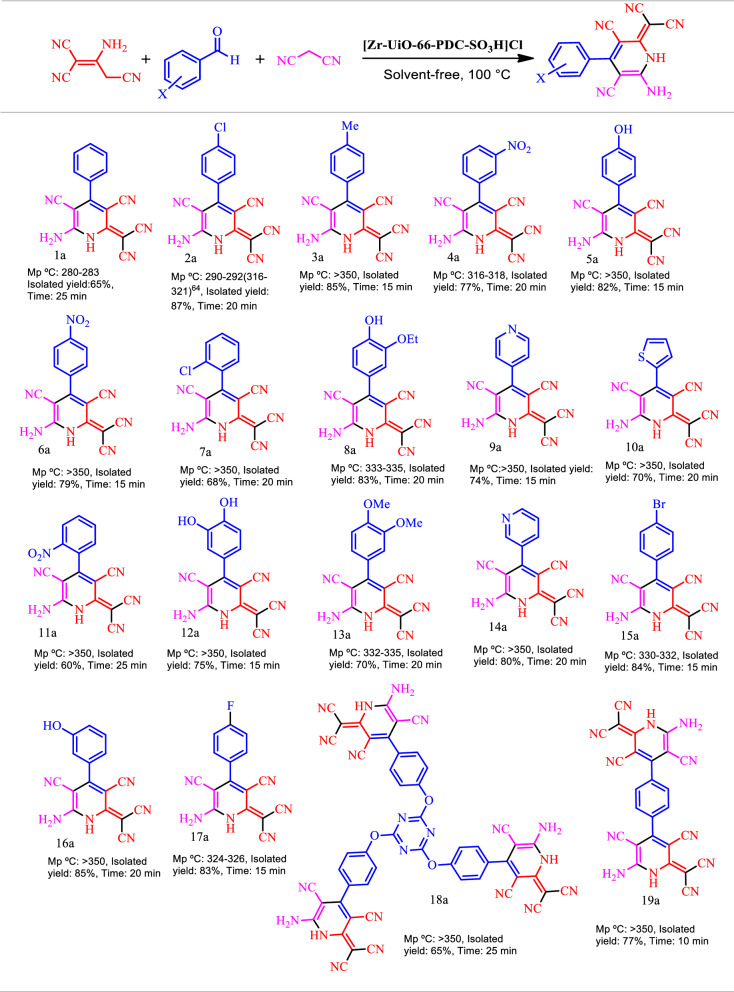
Effect of different amounts of catalyst, temperature and solvent (5.0 mL) in the synthesis dicyanomethylene pyridine.

The obtained results from model reaction under argon and nitrogen atmospheres verified abovementioned our suggestion for the latter step. Literature survey shows that there is no rational and clear stepwise mechanism for synthesis of the presented molecules^64,65^. Herein, we wish to present a comprehensive mechanism for a mentioned reaction so that its final step might progress through a cooperative vinylogous ABO in the absence of oxygen molecules. In the intermediates III and IV, sharing the electron density from the Endo and Exo nitrogens lone pairs to the vacant antibonding σ* orbital of SP^3^ C–H bond through vinylic C=C double bonds support the unusual hydride transfer for releasing the molecular hydrogen (H_2_). Recently, we have been named this phenomenon a new term entitled a cooperative vinylogous ABO and its development for various catalytic systems is our main research interest^30–38^. The described ABO mechanism is in good agreement with the "vinylogous anomeric effect" concept which at first, had been introduced by Katritzky^66^.

**Figure 10:**
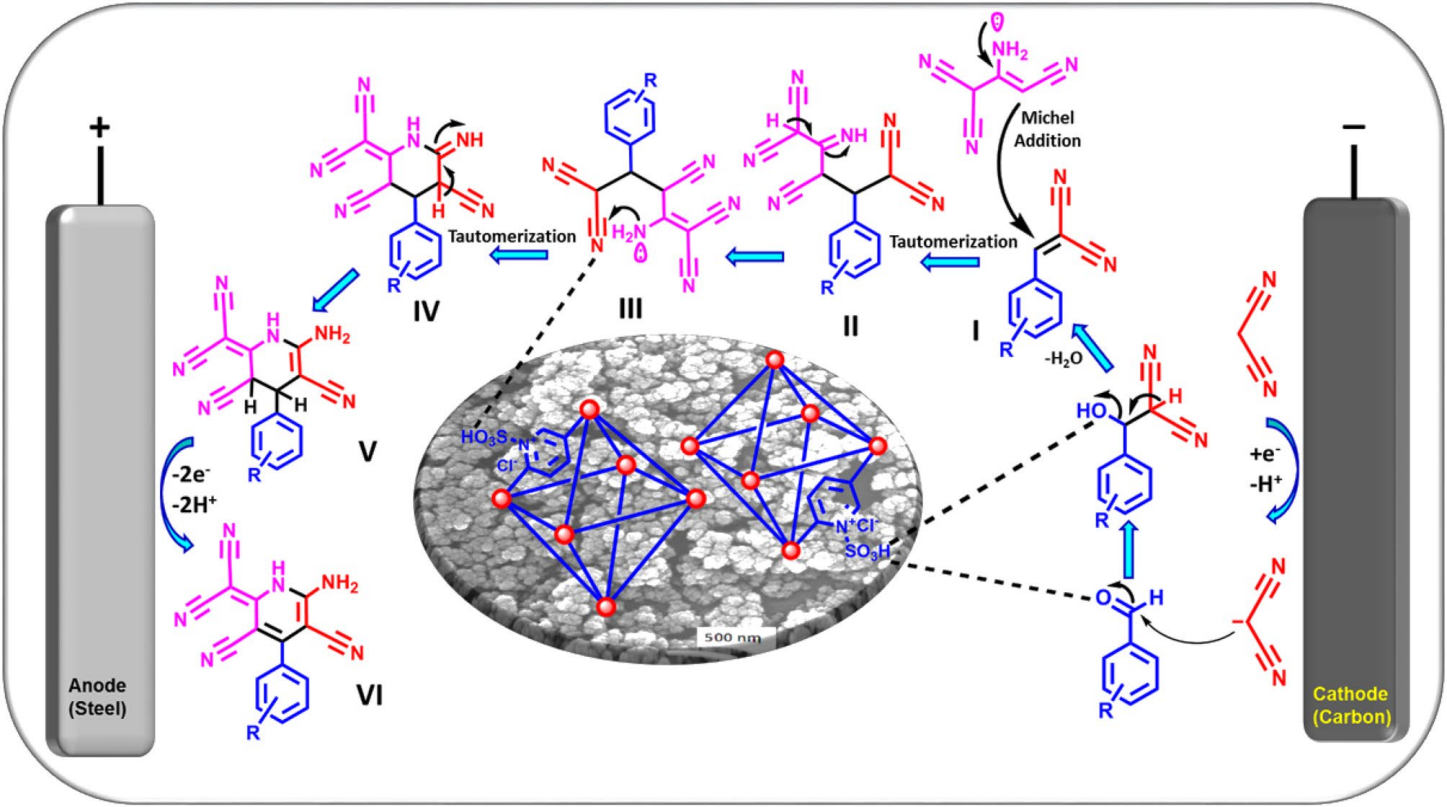
Convergent paired electrochemical synthesis of dicyanomethylene pyridine compounds.

At the second step of this work, [Zr-UiO-66-PDC-SO_3_H]Cl was employed for the preparation of dihydropyridine compounds via convergent paired electrosynthesis as a green and sustainable technique. To shed light on this fact, as shown in Fig. 10, an undivided home-made cell comprising two electrodes under constant current electrolysis was employed for this purpose.

The convergent paired electrosynthesis was started by applying constant current electrolysis (CCE) (1.0 mA cm^−2^) in ethanol and room temperature as green conditions. Upon the starting electrolysis, malononitrile can be activated to the methylene malononitrile on the cathodic electrode without the need for the ex-situ base additive. On the other hands, activation of the carbonyl group of aldehyde can be done by the SO_3_H functional group of employed catalysis, simultaneously. So, the suitable condition for preparing the knoevenagel’s adduct (I) will be provided through attacking the methylene malononitrile on the activated aldehyde and removing one H_2_O molecule. At the second step, 2-aminoprop-1-ene-1,1,3-tricarbonitrile can be attacked to the in-situ prepared knoevenagel’s adduct (I) via the Michael addition reaction.

After tautomerization step (II), the produced intermediate (III) undergoes an intramolecular cyclocondensation by effective activation of nitrile functional group via SO_3_H functional group of employed catalysis.

Finally, in order to the implementation and completing of the convergent paired electrosynthesis the produced intermediate (V) after tautomerization step (IV), can be oxidized on the anodic electrode by releasing two electron/two proton, in order to harvest the final product (VI). It should be noted, the progress and yield of a reaction in the absence of the [Zr-UiO-66-PDC-SO_3_H]Cl catalyst were slow and low.

It is noteworthy to mention that, the higher applied currents lead to the occurrence of side reactions like polymerization of malononitrile and the lower applied currents lead to prolonged reaction time or low yields due to the inactivation of malononitrile. Table 3 indicate the optimization of applied current density at the electrolysis condition.

**Table 3:** Optimization of conditions for the synthesis of dicyanomethylene pyridine.


Substrate	Current density (mA cm^−2^)	Isolated yield (%)
4-Chlorobenzaldehyde	0.5	35
4-Chlorobenzaldehyde	1.0	85
4-Chlorobenzaldehyde	1.5	25

Table 4 indicates the results of the model reactions based on the electronic properties of substitute groups on the benzaldehyde. The data presented in Table 4 shows the applied procedure have a satisfying performance for electrosynthesis of related dihydropyridine derivatives in a one-pot reaction with a 63–85% overall yield. It is noteworthy to mention that, the higher product yield for aldehydes with electron-withdrawing groups, 4-Chlorobenzaldehyde, is comparable and even greater than that of their simple (benzaldehyde) and/or electron-donating (4-methylbenzaldehyde) group homologues. These results may be caused by more efficient deprotonation of related aldehydes bearing electron-withdrawing groups lead to an intermediate under the proposed mechanism. So, the obtained trend may be repeated for the other homologue aldehydes based on the electron characteristics of substituted groups with acceptable tolerance.

**Table 4:**
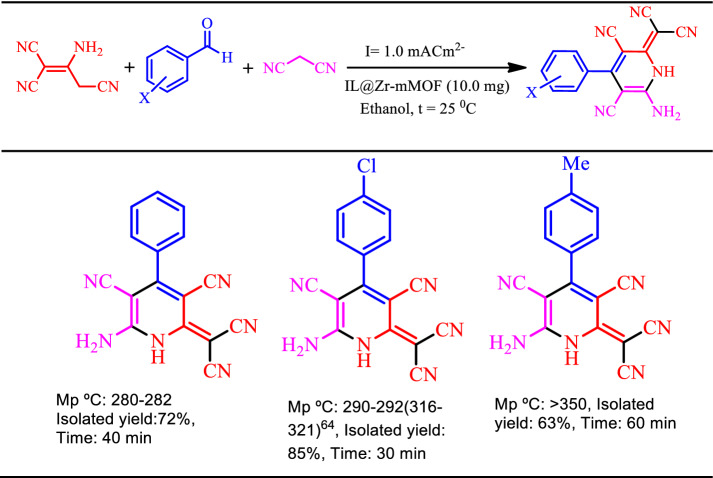
Electrochemical synthesis of dicyanomethylene pyridine derivatives in the presence of [Zr-UiO-66-PDC-SO_3_H]Cl as a catalyst. At *I* = 1.0 mA cm^−2^.

To evaluate the performance of [Zr-UiO-66-PDC-SO_3_H]Cl as an efficient catalyst for the synthesis of dicyanomethylene pyridine, we have tested different acid catalysts (organic and inorganic) by reaction of 4-chloro benzaldehyde (1.0 mmol, 0.14 g), 2-aminoprop-1-ene-1,1,3-tricarbonitrile (1.0 mmol, 0.132 g), and malononitrile (1.1 mmol, 0.072 g) in Table 5. As a result in Table 5, [Zr-UiO-66-PDC-SO_3_H]Cl is the best catalyst for the synthesis of dicyanomethylene pyridine.

**Table 5:** Evaluation of various catalysts for the synthesis of dicyanomethylene pyridine in comparison with [Zr-UiO-66-PDC-SO_3_H]Cl.


Entry	Catalyst	(mol%)	Time (min)	Yield^a^ (%)
1	SSA	10.0 mg	120	Trace
2	H_2_SO_4_	10	120	20
3	Fe_3_O_4_	10.0 mg	120	18
4	*p*-TSA	10	120	-
5	[PVI-SO_3_H]Cl	10.0 mg	120	43
6	MIL-100(Cr)/NHEtN(CH_2_PO_3_H_2_)_2_	10.0 mg	120	56
7	Trichloroisocyanuric acid	10	120	-
8	Al(HSO_4_)_3_	10	120	-
9	Mg(NO_3_)_2_.6H_2_O	10	120	28
10	NaHSO_4_	10	120	-
11	NH_4_NO_3_	10	120	-
12	FeCl_3_	10	120	38
13	H_3_[p(Mo_3_O_10_)_4_]·XH_2_O	10	120	-
14	Zn(NO_3_)_2_·6H_2_O	10	120	25
15	KOH	10	120	Trace
16	This work (Method A)	10	20	87

^a^Isolated yield.

**Figure 11:**
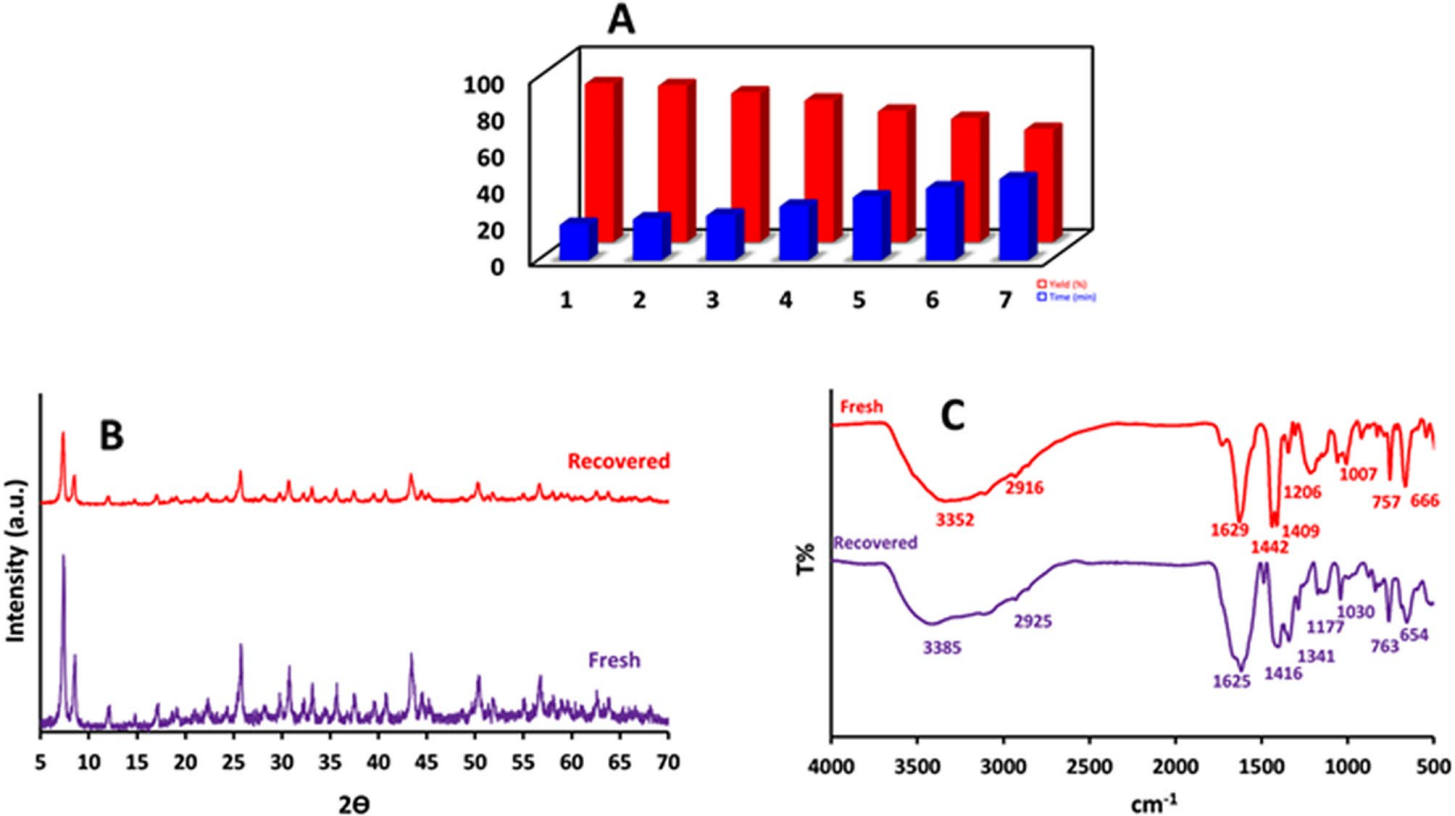
(**A**) Recyclability of [Zr-UiO-66-PDC-SO_3_H]Cl at the synthesis (2-methyl-1*H*-indol-3-yl)-pyrazolo[3,4-*b*]pyridine derivatives. (**B**,**C**) The characterization of reused catalysts after six runs using PXRD and FT-IR spectra.

The results of catalytic activity and reusability of [Zr-UiO-66-PDC-SO_3_H]Cl are shown in Fig. 11. [Zr-UiO-66-PDC-SO_3_H]Cl can be separated by centrifugation and reused without significantly reducing its catalytic reactivity. For this purpose, the recyclability of the catalyst was studied on the reaction of 4-chlorobenzaldehyde (1.0 mmol, 0.14 g), 2-aminoprop-1-ene-1,1,3-tricarbonitrile (1.0 mmol, 0.132 g), and malononitrile (1.1 mmol, 0.072 g) as a model reaction under the above mentioned optimized reaction conditions. The results show that [Zr-UiO-66-PDC-SO_3_H]Cl can be reused up to six times without noticeable changes in its catalytic activity. To confirm the above results, [Zr-UiO-66-PDC-SO_3_H]Cl was also analyzed by XRD and FT-IR spectra after its use in the reaction. These spectra were same as those of the fresh catalyst (Fig. 11).

## Conclusions

In this study, we have introduced a novel Zr-metal–organic frameworks [Zr-UiO-66-PDC-SO_3_H]Cl as a mesoporous catalyst. This catalyst was tested for the preparation of various novel dicyanomethylene pyridine via a cooperative vinylogous anomeric based oxidation mechanism. The topology of the [Zr-UiO-66-PDC-SO_3_H]Cl was also characterized by SEM and TEM images. As well as, the thermal and solvent stability of the catalyst is high after the reaction of SO_3_HCl with [Zr-UiO-66-PDC]. Furthermore, the major advantages of the presented work are mild and green conditions, high yields, short reaction times, facile workup and reusability of the described [Zr-UiO-66-PDC-SO_3_H]Cl. Also, this paper provides a green and promising electrochemical procedure for the preparation of mesoporous (UiO-66-PDC). From the standpoint of environmental issues, synthesis of these compounds can be performed without the need for any ex-situ chemical agent such as a base or probase. On the other hands, the use of electricity eliminates the need for high temperature and pressure, which is the most outstanding features of this study. Furthermore, the convergent paired electrosynthesis of dicyanomethylene pyridine derivatives with the prepared catalyst was performed as an environmentally friendly technique under green conditions, at room temperature and pressure. We think that the present work is a promising insight for inter and multidisciplinary research, rational design, syntheses and applications of task-specific MOFs and bioactive molecules.

## Materials and methods

Pyridine-2,5-dicarboxylic acid (H_2_PDC) (Merck, 95%), Zirconium tetrachloride (ZrCl_4_) (Sigma Aldrich, 98%), Potassium Nitrate (KNO_3_) (Sigma-Aldrich, 99%), formic acid (HCOOH) (Merck, 37%), Ethanol (C_2_H_5_OH) (Merck, 99%), Lithium Perchlorate (LiClO_4_) (Merck, 99%) and other materials (Merck) were reagent-grade materials and used as received without further purification. All solutions were prepared at room temperature. The known products were identified by comparison of their melting points and spectral data with those reported in the literature. To scrutinize the progress of the reaction, silica gel SIL G/UV 254 plates were used. From the model of the BRUKER Ultrashield FT-NMR spectrometer (δ in ppm) were recorded ^1^H NMR (600 or 400 MHz) and ^13^C NMR (151 or 101 MHz). Recorded on a Büchi B-545 apparatus in open capillary tubes were melting points. The PerkinElmer PE-1600-FTIR device was recorded for infrared spectra of compounds. SEM was performed using a scanning electron microscope for field publishing made by TE-SCAN. Thermal gravimetry (TG), differential thermal gravimetric (DTG) and differential thermal (DTA) were analyzed by a Perkin Elmer (Model: Pyris 1). BET and BJH were analyzed by BELSORP-mini ii high precision Surface area and pore size.

### Electrosynthesis setup

Electrosynthesis of catalyst and dicyanomethylene pyridine compounds were performed in a homemade undivided two-electrode cell. The cell consists of a cap glass bottle containing a precursor solution, carbon plate as working electrode (100 mm × 20 mm × 5 mm) and the U-shape stainless steel sheet as the auxiliary electrode. All of the electrochemical synthesis experiments were done at room temperature and pressure. Electrosynthesis of [Zr-UiO-66-PDC-SO_3_H]Cl and dicyanomethylene pyridine derivatives were accomplished by applying a suitable current density for a specified period.

### Chemical procedure for the preparation of Zr-UiO-66-PDC

**Figure 12:**
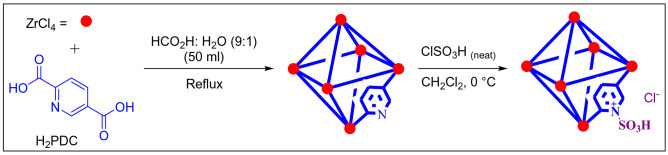
Chemical synthesis of [Zr-UiO-66-PDC-SO_3_H]Cl as a functionalized MOF catalyst.

In a 100 mL round-bottomed flask, a mixture of pyridine-2,5-dicarboxylic acid (H_2_PDC) (0.366 g, 2.2 mmol) and ZrCl_4_ (0.512 g, 2.2 mmol) with formic acid and H_2_O (9:1) 50 mL as solvent were stirred at 120 °C for 3 h under reflux conditions^13^. After this time, the suspension was filtered by centrifugation (2000 rpm, 20 min). Then, to the sediment was added H_2_O which was separated by centrifugation (2000 rpm, 20 min, 3 runs). Finally, a white precipitate was dried under a powerful vacuum at 90 °C to give Zr-UiO-66-PDC (Fig. 12).

### General procedure for the preparation of [Zr-UiO-66-PDC-SO_3_H]Cl

In a 50 mL round-bottomed flask, a mixture of Zr-UiO-66-PDC (1.0 mmol, 0.282 g) and chlorosulfonic acid (1.0 mmol, 0.067 mL) in dry CH_2_Cl_2_ (20.0 mL) at 0 °C were stirred for 2 h. Then, the sediment appeared which was filtered by centrifugation (1000 rpm, 5 min, 2 times) and dried under vacuum to obtain white precipitated as [Zr-UiO-66-PDC-SO_3_H]Cl (Fig. 12).

### Chemical procedure for the preparation of dicyanomethylene pyridine derivatives using [Zr-UiO-66-PDC-SO_3_H]Cl as an efficient catalyst

In a 15 mL round-bottomed flask, a mixture of aldehyde (1.0 mmol, 0.398 g), 2-aminoprop-1-ene-1,1,3-tricarbonitrile (1.0 mmol, 0.132 g ) and malononitrile (1.1 mmol, 0.073 g) and [Zr-UiO-66-PDC-SO_3_H]Cl (10.0 mg) as catalyst was stirred under solvent-free conditions at 100 °C. After completion of the reaction which was followed by TLC (*n-*hexane: ethyl acetate; 7:3), the reaction mixture was cool down to room temperature. Then, the mixture was added 10 mL ethanol and the catalyst was subsequently removed by centrifugation (1000 rpm). Finally, the product was recrystallized with EtOH (Fig. 2).

### Electrochemical procedure for the preparation of Zr-UiO-66-PDC

In a typical procedure, (0.512 g, 2.2 mmol) of zirconium tetrachloride as a cation source, (0.366 g, 2.2 mmol) of pyridine-2,5-dicarboxylic acid (H_2_PDC) as ligand and (0.127 g, 0.1 mmol) potassium nitrate (KNO_3_) as a supporting electrolyte were dissolved in the 50.0 mL aqueous solution of formic acid (H_2_O/formic acid; 1/9). The solution was stirred at room temperature for 30 min before the electrolysis (30 mA cm^−2^ for 1800s). After electrolysis, the solution was centrifuged at 5000 rpm for 5 min, and the precipitate was washed twice with distilled water and ethanol. The final MOF was then aged overnight at 100 °C.

### Electrochemical procedure for convergent paired electrosynthesis of dicyanomethylene pyridines

The convergent paired electrosynthesis of dicyanomethylene pyridines derivatives was carried out in 50 mL of ethanol containing aldehyde derivatives (1.0 mmol), malononitrile (0.066 g, 1.0 mmol), 2-aminoprop-1-ene-1,1,3-tricarbonitrile (0.132 g, 1.0 mmol) and [Zr-UiO-66-PDC-SO_3_H]Cl (10.0 mg) as catalyst under stirring at 1.0 mA cm^−2^. The progress of the reaction was followed by thin-layer chromatography (*n*-hexane; ethylacetate). Also, the final product was separated by large scale thin-layer chromatography on the silica gel plate.

### Characteristic of the products

#### 6-Amino-2-(dicyanomethylene)-4-phenyl-1,2-dihydropyridine-3,5-dicarbonitrile (1a)

Yellow solid; Mp: 280–283 °C; IR (KBr): υ (cm^−1^) = 3374, 3306, 3215, 2218, 2191, 1642. ^1^H NMR (400 MHz, DMSO-*d*_6_) δ 7.80 (s, 3H), 7.60 – 7.53 (m, 3H), 7.48 (d, *J* = 1.3 Hz, 2H). ^13^C NMR (101 MHz, DMSO-*d*_6_) δ 160.5, 160.4, 157.8, 135.3, 129.7, 128.4, 128.3, 116.2, 115.9, 85.4, 80.7, 43.6 (See SI).

#### 6-Amino-4-(4-chlorophenyl)-2-(dicyanomethylene)-1,2-dihydropyridine-3,5-dicarbonitrile (2a)

Yellow solid; Mp: 290–262 °C; IR (KBr): υ (cm^−1^) = 3363, 3302, 3211, 2228, 2217, 2191, 1645. ^1^H NMR (600 MHz, DMSO-*d*_*6*_) δ 7.59 (d, J = 8.4 Hz, 2H), 7.46 (d, J = 8.4 Hz, 2H), 6.52 (s, 3H). ^13^C NMR (151 MHz, DMSO-*d*_6_) δ 161.6, 159.5, 158.5, 134.9, 134.9, 130.8, 129.1, 116.8, 116.5, 85.6, 81.0, 44.2 (See SI).

#### 6-Amino-2-(dicyanomethylene)-4-(p-tolyl)-1,2-dihydropyridine-3,5-dicarbonitrile (3a)

Yellow solid; Mp: > 350 °C; IR (KBr): υ (cm^−1^) = 3483, 3371, 3202, 2213, 2200, 2171, 1620. ^1^H NMR (400 MHz, DMSO-*d*_6_) δ 7.29 (s, 4H), 6.87 (s, 2H), 2.38 (s, 3H). ^13^C NMR (101 MHz, DMSO-*d*_6_) δ 162.7, 159.7, 158.7, 139.0, 132.8, 128.9, 128.3, 116.9, 116.6, 85.0, 80.3, 20.9 (See SI).

#### 6-Amino-2-(dicyanomethylene)-4-(3-nitrophenyl)-1,2-dihydropyridine-3,5-dicarbonitrile (4a)

Yellow solid; Mp: 316–318 °C; IR (KBr): υ (cm^−1^) = 3438, 3339, 3213, 2197, 2181, 1657, 1556, 1352. ^1^H NMR (600 MHz, DMSO-*d*_6_) δ 8.38 – 8.35 (m, 1H), 8.33 – 8.30 (m, 1H), 7.93 (d, *J* = 7.7 Hz, 1H), 7.83 (t, *J* = 8.0 Hz, 1H), 7.05 (s, 2H). ^13^C NMR (151 MHz, DMSO-*d*_6_) δ 163.0, 159.0, 157.9, 148.0, 137.8, 135.9, 130.8, 124.8, 123.9, 117.1, 116.9, 85.3, 80.7, 44.3 (See SI).

#### 6-Amino-2-(dicyanomethylene)-4-(4-hydroxyphenyl)-1,2-dihydropyridine-3,5-dicarbonitrile (5a)

Yellow solid; Mp: > 350 °C; IR (KBr): υ (cm^−1^) = 3342, 3227, 2183, 1652, 1553. ^1^H NMR (600 MHz, DMSO-*d*_6_) δ 9.85 (s, 1H), 7.23 (d, *J* = 8.5 Hz, 2H), 6.89 – 6.78 (m, 4H). ^13^C NMR (151 MHz, DMSO-*d*_6_) δ 163.4, 160.1, 159.3, 159.1, 130.6, 126.6, 117.7, 117.3, 115.5, 85.6, 80.7, 43.6 (See SI).

#### 6-Amino-2-(dicyanomethylene)-4-(4-nitrophenyl)-1,2-dihydropyridine-3,5-dicarbonitrile (6a)

Yellow solid; Mp: > 350 °C; IR (KBr): υ (cm^−1^) = 3336, 2196, 1655, 1556, 1508, 1360. ^1^H NMR (600 MHz, DMSO-*d*_6_) δ 8.35 (d, *J* = 8.7 Hz, 2H), 7.74 (d, *J* = 8.7 Hz, 2H), 7.05 (s, 2H). ^13^C NMR (151 MHz, DMSO-*d*_6_) δ 162.9, 159.0, 158.3, 148.5, 142.9, 130.7, 124.1, 116.9, 116.7, 85.0, 80.4, 44.3 (See SI).

#### 6-Amino-4-(2-chlorophenyl)-2-(dicyanomethylene)-1,2-dihydropyridine-3,5-dicarbonitrile (7a)

Yellow solid; Mp: > 350 °C; IR (KBr): υ (cm^−1^) = 3438, 3342, 3231, 2195, 2164, 1636, 1557. ^1^H NMR (600 MHz, DMSO-*d*_6_) δ 7.61 (dd, *J* = 7.9, 1.3 Hz, 1H), 7.50 (td, *J* = 7.7, 1.9 Hz, 1H), 7.47 (td, *J* = 7.5, 1.4 Hz, 1H), 7.40 (dd, *J* = 7.4, 1.8 Hz, 1H), 7.00 (s, 2H). ^13^C NMR (151 MHz, DMSO-*d*_6_) δ 162.7, 158.9, 157.9, 135.5, 131.6, 131.4, 130.7, 130.0, 128.0, 116.6, 116.3, 86.0, 81.1, 44.0 (See SI).

#### 6-Amino-2-(dicyanomethylene)-4-(3-ethoxy-4-hydroxyphenyl)-1,2-dihydropyridine-3,5-dicarbonitrile (8a)

Yellow solid; Mp: 333–335 °C; IR (KBr): υ (cm^−1^) = 3431, 3342, 3236, 2198, 2165, 1649, 1554. ^1^H NMR (600 MHz, DMSO-*d*_6_) δ 9.38 (s, 1H), 6.97 – 6.94 (m, 1H), 6.87 (d, *J* = 8.1 Hz, 1H), 6.85 – 6.78 (m, 3H), 4.06 (q, *J* = 7.0 Hz, 2H), 1.36 – 1.33 (m, 3H). ^13^C NMR (151 MHz, DMSO-*d*_6_) δ 163.3, 160.1, 159.3, 148.6, 146.5, 126.8, 122.1, 117.8, 117.4, 115.7, 114.6, 85.6, 80.8, 64.3, 43.6, 15.1 (See SI).

#### 6-Amino-2-(dicyanomethylene)-1,2-dihydro-[4,4'-bipyridine]-3,5-dicarbonitrile (9a)

Yellow solid; Mp: > 350 °C; IR (KBr): υ (cm^−1^) = 3443, 3345, 3224, 2211, 2195, 2175, 1650, 1569. ^1^H NMR (600 MHz, DMSO-*d*_6_) δ 8.72 (d, *J* = 5.7 Hz, 2H), 7.46 (d, *J* = 4.4 Hz, 2H), 7.04 (s, 2H). ^13^C NMR (151 MHz, DMSO-*d*_6_) δ 162.9, 159.0, 157.7, 150.3, 144.2, 123.6, 116.9, 116.6, 84.7, 80.1, 44.3 (See SI).

#### 6-Amino-2-(dicyanomethylene)-4-(thiophen-2-yl)-1,2-dihydropyridine-3,5-dicarbonitrile (10a)

Yellow solid; Mp: > 350 °C; IR (KBr): υ (cm^−1^) = 3481, 3345, 3211, 2213, 2189, 2163, 1624, 1555. ^1^H NMR (600 MHz, DMSO-*d*_6_) δ 7.82 (d, *J* = 4.9 Hz, 1H), 7.37 (d, *J* = 3.5 Hz, 1H), 7.23 – 7.18 (m, 1H), 6.97 (s, 2H). ^13^C NMR (151 MHz, DMSO-*d*_6_) δ 163.4, 159.3, 152.2, 135.2, 130.4, 129.6, 127.9, 117.3, 116.9, 85.8, 81.0, 44.2 (See SI).

#### 6-Amino-2-(dicyanomethylene)-4-(2-nitrophenyl)-1,2-dihydropyridine-3,5-dicarbonitrile (11a)

Yellow solid; Mp: > 350 °C; IR (KBr): υ (cm^−1^) = 3437, 3341, 3230, 2218, 2195, 1637, 1560, 1517, 1351. ^1^H NMR (600 MHz, DMSO-*d*_6_) δ 8.27 (d, *J* = 8.2 Hz, 1H), 7.92 (t, *J* = 7.5 Hz, 1H), 7.80 (t, *J* = 7.9 Hz, 1H), 7.60 (d, *J* = 7.5 Hz, 1H), 7.05 (s, 2H). ^13^C NMR (151 MHz, DMSO-*d*_6_) δ 170.8, 162.6, 158.8, 158.1, 147.3, 135.2, 131.8, 131.6, 131.3, 125.4, 116.6, 116.4, 84.9, 80.2, 60.2, 44.1, 21.2, 14.6 (See SI).

#### 6-Amino-2-(dicyanomethylene)-4-(3,4-dihydroxyphenyl)-1,2-dihydropyridine-3,5-dicarbonitrile (12a)

Yellow solid; Mp: > 350 °C; IR (KBr): υ (cm^−1^) = 3457, 3334, 3225, 2197, 2168, 1650, 1560. ^1^H NMR (600 MHz, DMSO-*d*_6_) δ 9.33 (s, 1H), 9.26 (s, 1H), 6.80 (d, *J* = 8.0 Hz, 3H), 6.77 (s, 1H), 6.67 (d, *J* = 8.1 Hz, 1H) (See SI).

#### 6-Amino-2-(dicyanomethyl)-4-(3,4-dimethoxyphenyl)-1,2-dihydropyridine-3,5-dicarbonitrile (13a)

Yellow solid; Mp: 332–335 °C; IR (KBr): υ (cm^−1^) = 3504, 3489, 3374, 2216, 2194, 2172, 1613, 1551. ^1^H NMR (400 MHz, DMSO-*d*_6_) δ 7.07 (d, *J* = 8.3 Hz, 1H), 7.03 (d, *J* = 2.1 Hz, 1H), 6.97 (dd, *J* = 8.2, 2.1 Hz, 1H), 6.88 (s, 2H), 3.82 (s, 3H), 3.79 (s, 3H). ^13^C NMR (101 MHz, DMSO-*d*_6_) δ 162.7, 159.3, 158.8, 149.6, 148.0, 127.7, 121.3, 117.1, 116.7, 112.2, 111.2, 85.1, 80.3, 55.5, 55.4, 43.1, 40.0 (See SI).

#### 6'-Amino-2'-(dicyanomethylene)-1',2'-dihydro-[3,4'-bipyridine]-3',5'-dicarbonitrile (14a)

Brown solid; Mp: > 350 °C; IR (KBr): υ (cm^−1^) = 3389, 3317, 3161, 2209, 2189, 2156, 1654, 1577, 1514. ^1^H NMR (400 MHz, DMSO-*d*_6_) δ 8.79 – 8.47 (m, 2H), 7.91 (dt, *J* = 7.9, 2.0 Hz, 1H), 7.55 (dd, *J* = 7.9, 4.9 Hz, 1H), 7.03 (s, 2H). ^13^C NMR (101 MHz, DMSO-*d*_6_) δ 162.52, 158.57, 156.38, 150.38, 148.47, 136.39, 131.89, 123.40, 116.65, 116.43, 85.08, 80.40, 40.03 (See SI).

#### 6-Amino-4-(4-bromophenyl)-2-(dicyanomethylene)-1,2-dihydropyridine-3,5-dicarbonitrile (15a)

White solid; Mp: 330–332 °C; IR (KBr): υ (cm^−1^) = 3451, 3339, 3224, 2214, 2194, 2170, 1674, 1572, 1511. ^1^H NMR (400 MHz, DMSO-*d*_6_) δ 7.77 – 7.64 (m, 2H), 7.46 – 7.30 (m, 2H), 6.97 (s, 2H). ^13^C NMR (101 MHz, DMSO-*d*_6_) δ 162.5, 158.5, 158.5, 135.0, 131.4, 130.6, 123.0, 116.7, 116.4, 84.8, 80.1, 40.0 (See SI).

#### 6-Amino-2-(dicyanomethylene)-4-(3-hydroxyphenyl)-1,2-dihydropyridine-3,5-dicarbonitrile (16a)

Yellow solid; Mp: > 350 °C; IR (KBr): υ (cm^−1^) = 3476, 3330, 3218, 2202, 2171, 1625, 1548. ^1^H NMR (600 MHz, DMSO-*d*_6_) δ 9.72 (s, 1H), 7.28 (t, *J* = 7.8 Hz, 1H), 6.94 – 6.84 (m, 3H), 6.77 (d, *J* = 7.5 Hz, 1H), 6.73 (s, 1H) (See SI).

#### 6-Amino-2-(dicyanomethylene)-4-(4-fluorophenyl)-1,2-dihydropyridine-3,5-dicarbonitrile (17a)

Yellow solid; Mp: 324–326 °C; IR (KBr): υ (cm^−1^) = 3449, 3374, 3309, 3216, 2212, 2194, 2171, 1646. ^1^H NMR (600 MHz, DMSO-*d*_6_) δ 7.51 – 7.45 (m, 2H), 7.34 (t, *J* = 8.6 Hz, 2H), 6.97 (s, 2H) (See SI).

#### 4,4',4''-(((1,3,5-Triazine-2,4,6-triyl)tris(oxy))tris(benzene-4,1-diyl))tris(6-amino-2-(dicyanomethylene)-1,2-dihydropyridine-3,5-dicarbonitrile) (18a)

Yellow solid; Mp: > 350 °C; IR (KBr): υ (cm^−1^) = 3504, 3374, 3224, 2215, 2194, 2166, 1613, 1551. ^1^H NMR (400 MHz, DMSO-*d*_6_) δ 7.53 (td, *J* = 8.5, 4.3 Hz, 1H), 7.49 – 7.41 (m, 2H), 7.38 (d, *J* = 8.6 Hz, 1H), 6.95 (s, 1H), 6.91 (s, 1H). ^13^C NMR (101 MHz, DMSO-*d*_6_) δ 173.3, 163.2, 159.2, 159.1, 153.2, 152.5, 133.9, 132.5, 130.6, 130.1, 122.0, 121.7, 120.6, 117.3, 117.0, 85.5, 80.8, 40.5, 40.3, 40.1 (See SI).

#### 4,4'-(1,4-Phenylene)bis(6-amino-2-(dicyanomethylene)-1,2-dihydropyridine-3,5-dicarbonitrile) (19a)

Yellow solid; Mp: > 350 °C; IR (KBr): υ (cm^−1^) = 3395, 3334, 3228, 2192, 2158, 1650, 1549, 1429. ^1^H NMR (400 MHz, DMSO-*d*_6_) δ 7.55 (s, 1H), 6.94 (s, 1H). ^13^C NMR (101 MHz, DMSO-*d*_6_) δ 159.2, 144.3, 139.6, 135.2, 135.0, 134.8, 132.4, 131.5, 129.1, 128.9, 126.5, 126.4, 121.7, 121.2, 120.1, 119.6, 118.4, 116.8, 111.1, 111.0, 110.8, 102.8, 42.3 (See SI).

## Supplementary Information

Supplementary Information.
