# Supplementary material for: Synthesis and application of [Zr-UiO-66-PDC-SO3H]Cl MOFs to the preparation of dicyanomethylene pyridines via chemical and electrochemical methods
Source: Sci Rep. 2021 Aug 19;11:16817. doi: 10.1038/s41598-021-96001-7 (PMC8377142; doi:10.1038/s41598-021-96001-7)
Supplement: Supplementary file 1 — Supplementary Information. [file 41598_2021_96001_MOESM1_ESM.pdf]

Supporting Information

Synthesis and Application of [Zr-UiO-66-PDC-SO_3H]Cl MOFs to the Preparation of Dicyanomethylene Pyridines via Chemical and Electrochemical Methods

Amir Mohammad Naseri [a], Mahmoud Zarei*[a], Saber Alizadeh*[b], Saeed Babaee [a], Mohammad Ali Zolfigol*[a], Davood Nematollahi*[b], Jalal Arjomandi[c], Hu Shi[d]

[a] Department of Organic Chemistry, Faculty of Chemistry, Bu-Ali Sina University, Hamedan 6517838683, Tel: +988138282807, Fax: +988138380709 Iran.

E-Mail: mahmoud8103@yahoo.com , zolfi@basu.ac.ir & mzolfigol@yahoo.com

[b] Department of Analytical Chemistry, Faculty of Chemistry, Bu-Ali Sina University, Hamedan 6517838683, Iran.

E-Mail: s.alizade66@yahoo.com & s.alizadeh93@basu.ac.ir, nemat@basu.ac.ir, nematollahid@gmail.com

[c] Department of Physical Chemistry, Faculty of Chemistry, Bu-Ali Sina University, Hamedan 6517838683, Iran.

[d] School of Chemistry and Chemical Engineering, Institute of Molecular Science, Shanxi University, Taiyuan 030006, China.

E-Mail: hshi@sxu.edu.cn.

6-Amino-2-(dicyanomethylene)-4-phenyl-1,2-dihydropyridine-3,5-dicarbonitrile (a) 6

6-Amino-4-(4-chlorophenyl)-2-(dicyanomethylene)-1,2-dihydropyridine-3,5-dicarbonitrile (b)................ 6

6-Amino-2-(dicyanomethylene)-4-(p-tolyl)-1,2-dihydropyridine-3,5-dicarbonitrile (c)............................. 6

6-Amino-2-(dicyanomethylene)-4-(3-nitrophenyl)-1,2-dihydropyridine-3,5-dicarbonitrile (d) 7

6-Amino-2-(dicyanomethylene)-4-(4-hydroxyphenyl)-1,2-dihydropyridine-3,5-dicarbonitrile (e)............ 7

6-Amino-2-(dicyanomethylene)-4-(4-nitrophenyl)-1,2-dihydropyridine-3,5-dicarbonitrile (f) 7

6-Amino-4-(2-chlorophenyl)-2-(dicyanomethylene)-1,2-dihydropyridine-3,5-dicarbonitrile (g)................ 7

6-Amino-2-(dicyanomethylene)-4-(3-ethoxy-4-hydroxyphenyl)-1,2-dihydropyridine-3,5-dicarbonitrile (h) 8

6-Amino-2-(dicyanomethylene)-1,2-dihydro-[4,4'-bipyridine]-3,5-dicarbonitrile (i) 8

6-Amino-2-(dicyanomethylene)-4-(thiophen-2-yl)-1,2-dihydropyridine-3,5-dicarbonitrile (j) 8

6-Amino-2-(dicyanomethylene)-4-(2-nitrophenyl)-1,2-dihydropyridine-3,5-dicarbonitrile (k) 8

6-Amino-2-(dicyanomethylene)-4-(3,4-dihydroxyphenyl)-1,2-dihydropyridine-3,5-dicarbonitrile (l)........ 9

6-Amino-2-(dicyanomethyl)-4-(3,4-dimethoxyphenyl)-1,2-dihydropyridine-3,5-dicarbonitrile (m) 9

6'-Amino-2'-(dicyanomethylene)-1',2'-dihydro-[3,4'-bipyridine]-3',5'-dicarbonitrile (n) 9

6-Amino-4-(4-bromophenyl)-2-(dicyanomethylene)-1,2-dihydropyridine-3,5-dicarbonitrile (o)............. 10

6-Amino-2-(dicyanomethylene)-4-(3-hydroxyphenyl)-1,2-dihydropyridine-3,5-dicarbonitrile (p) 10

6-Amino-2-(dicyanomethylene)-4-(4-fluorophenyl)-1,2-dihydropyridine-3,5-dicarbonitrile (q) 10

4,4',4''-(((1,3,5-Triazine-2,4,6-triyl)tris(oxy))tris(benzene-4,1-diyl))tris(6-amino-2-(dicyanomethylene)-1,2-dihydropyridine-3,5-dicarbonitrile) (r) 11

4,4'-(1,4-Phenylene)bis(6-amino-2-(dicyanomethylene)-1,2-dihydropyridine-3,5-dicarbonitrile) (s) 11

FT-IR spectrum of 6-amino-2-(dicyanomethylene)-4-phenyl-1,2-dihydropyridine-3,5-dicarbonitrile (a) 12

^{1}H NMR spectrum of 6-Amino-2-(dicyanomethylene)-4-phenyl-1,2-dihydropyridine-3,5-dicarbonitrile(a) 12

6-Amino-2-(dicyanomethylene)-4-phenyl-1,2-dihydropyridine-3,5-dicarbonitrile(a) 13

FT-IR spectrum of 6-amino-4-(4-chlorophenyl)-2-(dicyanomethylene)-1,2-dihydropyridine-3,5-dicarbonitrile (b) 13

^{1}H NMR spectrum of 6-amino-4-(4-chlorophenyl)-2-(dicyanomethylene)-1,2-dihydropyridine-3,5-dicarbonitrile (b) 14

^{13}C NMR spectrum of 6-amino-4-(4-chlorophenyl)-2-(dicyanomethylene)-1,2-dihydropyridine-3,5-dicarbonitrile (b) 14

FT-IR spectrum of 6-amino-2-(dicyanomethylene)-4-(p-tolyl)-1,2-dihydropyridine-3,5-dicarbonitrile (c) 15

^{1}H NMR spectrum of 6-Amino-2-(dicyanomethylene)-4-(p-tolyl)-1,2-dihydropyridine-3,5-dicarbonitrile (c) 15

^{13}C NMR spectrum of 6-Amino-2-(dicyanomethylene)-4-(p-tolyl)-1,2-dihydropyridine-3,5-dicarbonitrile (c) 16

FT-IR spectrum of 6-amino-2-(dicyanomethylene)-4-(3-nitrophenyl)-1,2-dihydropyridine-3,5-dicarbonitrile (d) 16

^{1}H NMR spectrum of 6-amino-2-(dicyanomethylene)-4-(3-nitrophenyl)-1,2-dihydropyridine-3,5-dicarbonitrile (d) 17

^{13}C NMR spectrum of 6-amino-2-(dicyanomethylene)-4-(3-nitrophenyl)-1,2-dihydropyridine-3,5-dicarbonitrile (d) 17

FT-IR spectrum of 6-amino-2-(dicyanomethylene)-4-(4-hydroxyphenyl)-1,2-dihydropyridine-3,5-dicarbonitrile (e) 18

^{1}H NMR spectrum of 6-amino-2-(dicyanomethylene)-4-(4-hydroxyphenyl)-1,2-dihydropyridine-3,5-dicarbonitrile (e) 18

^{13}C NMR spectrum of 6-amino-2-(dicyanomethylene)-4-(4-hydroxyphenyl)-1,2-dihydropyridine-3,5-dicarbonitrile (e) 19

FT-IR spectrum of 6-amino-2-(dicyanomethylene)-4-(4-nitrophenyl)-1,2-dihydropyridine-3,5-dicarbonitrile (f) 20

^{1}H NMR spectrum of 6-amino-2-(dicyanomethylene)-4-(4-nitrophenyl)-1,2-dihydropyridine-3,5-dicarbonitrile (f) 20

^{13}C NMR spectrum of 6-amino-2-(dicyanomethylene)-4-(4-nitrophenyl)-1,2-dihydropyridine-3,5-dicarbonitrile (f) 21

FT-IR spectrum of 6-amino-4-(2-chlorophenyl)-2-(dicyanomethylene)-1,2-dihydropyridine-3,5-dicarbonitrile (g) 22

^{1}H NMR spectrum of 6-amino-4-(2-chlorophenyl)-2-(dicyanomethylene)-1,2-dihydropyridine-3,5-dicarbonitrile (g) 22

^{13}C NMR spectrum of 6-amino-4-(2-chlorophenyl)-2-(dicyanomethylene)-1,2-dihydropyridine-3,5-dicarbonitrile (g) 23

FT-IR spectrum of 6-amino-2-(dicyanomethylene)-4-(3-ethoxy-4-hydroxyphenyl)-1,2-dihydropyridine-3,5-dicarbonitrile (h) 23

^{1}H NMR spectrum of 6-amino-2-(dicyanomethylene)-4-(3-ethoxy-4-hydroxyphenyl)-1,2-dihydropyridine-3,5-dicarbonitrile (h) 24

^{13}C NMR spectrum of 6-amino-2-(dicyanomethylene)-4-(3-ethoxy-4-hydroxyphenyl)-1,2-dihydropyridine-3,5-dicarbonitrile (h) 24

FT-IR spectrum of 6-amino-2-(dicyanomethylene)-1,2-dihydro-[4,4'-bipyridine]-3,5-dicarbonitrile (i) 25

^{1}H NMR spectrum of 6-amino-2-(dicyanomethylene)-1,2-dihydro-[4,4'-bipyridine]-3,5-dicarbonitrile (i) 25

^{13}C NMR spectrum of 6-amino-2-(dicyanomethylene)-1,2-dihydro-[4,4'-bipyridine]-3,5-dicarbonitrile (i) 26

FT-IR spectrum of 6-amino-2-(dicyanomethylene)-4-(thiophen-2-yl)-1,2-dihydropyridine-3,5-dicarbonitrile (j) 26

^{1}H NMR spectrum of 6-amino-2-(dicyanomethylene)-4-(thiophen-2-yl)-1,2-dihydropyridine-3,5-dicarbonitrile (j) 27

^{13}C NMR spectrum of 6-amino-2-(dicyanomethylene)-4-(thiophen-2-yl)-1,2-dihydropyridine-3,5-dicarbonitrile (j) 27

FT-IR spectrum of 6-amino-2-(dicyanomethylene)-4-(2-nitrophenyl)-1,2-dihydropyridine-3,5-dicarbonitrile (k) 28

^{1}H NMR spectrum of 6-amino-2-(dicyanomethylene)-4-(2-nitrophenyl)-1,2-dihydropyridine-3,5-dicarbonitrile (k) 28

^{13}C NMR spectrum of 6-amino-2-(dicyanomethylene)-4-(2-nitrophenyl)-1,2-dihydropyridine-3,5-dicarbonitrile (k) 29

FT-IR spectrum of 6-amino-2-(dicyanomethylene)-4-(3,4-dihydroxyphenyl)-1,2-dihydropyridine-3,5-dicarbonitrile (l) 29

^{1}H NMR spectrum of 6-amino-2-(dicyanomethylene)-4-(3,4-dihydroxyphenyl)-1,2-dihydropyridine-3,5-dicarbonitrile (l) 30

FT-IR spectrum of 6-amino-2-(dicyanomethyl)-4-(3,4-dimethoxyphenyl)-1,2-dihydropyridine-3,5-dicarbonitrile (m) 30

^{1}H NMR spectrum of 6-amino-2-(dicyanomethyl)-4-(3,4-dimethoxyphenyl)-1,2-dihydropyridine-3,5-dicarbonitrile (m) 31

^{13}C NMR spectrum of 6-amino-2-(dicyanomethyl)-4-(3,4-dimethoxyphenyl)-1,2-dihydropyridine-3,5-dicarbonitrile (m) 31

FT-IR spectrum of 6'-amino-2'-(dicyanomethylene)-1',2'-dihydro-[3,4'-bipyridine]-3',5'-dicarbonitrile (n) 32

^{1}H NMR spectrum of 6'-amino-2'-(dicyanomethylene)-1',2'-dihydro-[3,4'-bipyridine]-3',5'-dicarbonitrile (n) 32

^{13}C NMR spectrum of 6'-amino-2'-(dicyanomethylene)-1',2'-dihydro-[3,4'-bipyridine]-3',5'-dicarbonitrile (n) 33

FT-IR spectrum of 6-amino-4-(4-bromophenyl)-2-(dicyanomethylene)-1,2-dihydropyridine-3,5-dicarbonitrile (o) 33

^{1}H NMR spectrum of 6-amino-4-(4-bromophenyl)-2-(dicyanomethylene)-1,2-dihydropyridine-3,5-dicarbonitrile (o) 34

^{13}C NMR spectrum of 6-amino-4-(4-bromophenyl)-2-(dicyanomethylene)-1,2-dihydropyridine-3,5-dicarbonitrile (o) 34

FT-IR spectrum of 6-amino-2-(dicyanomethylene)-4-(3-hydroxyphenyl)-1,2-dihydropyridine-3,5-dicarbonitrile (p) 35

^{1}H NMR spectrum of 6-amino-2-(dicyanomethylene)-4-(3-hydroxyphenyl)-1,2-dihydropyridine-3,5-dicarbonitrile (p) 35

FT-IR spectrum of 6-amino-2-(dicyanomethylene)-4-(4-fluorophenyl)-1,2-dihydropyridine-3,5-dicarbonitrile (q) 36

^{1}H NMR spectrum of 6-amino-2-(dicyanomethylene)-4-(4-fluorophenyl)-1,2-dihydropyridine-3,5-dicarbonitrile (q) 36

FT-IR spectrum of 4,4',4''-(((1,3,5-triazine-2,4,6-triyl)tris(oxy))tris(benzene-4,1-diyl))tris(6-amino-2-(dicyanomethylene)-1,2-dihydropyridine-3,5-dicarbonitrile) (r) 37

^{1}H NMR spectrum of 4,4',4''-(((1,3,5-triazine-2,4,6-triyl)tris(oxy))tris(benzene-4,1-diyl))tris(6-amino-2-(dicyanomethylene)-1,2-dihydropyridine-3,5-dicarbonitrile) (r) 37

^{13}C NMR spectrum of 4,4',4''-(((1,3,5-triazine-2,4,6-triyl)tris(oxy))tris(benzene-4,1-diyl))tris(6-amino-2-(dicyanomethylene)-1,2-dihydropyridine-3,5-dicarbonitrile) (r) 38

FT-IR spectrum of 4,4'-(1,4-phenylene)bis(6-amino-2-(dicyanomethylene)-1,2-dihydropyridine-3,5-dicarbonitrile) (s) 38

^{1}H NMR spectrum of 4,4'-(1,4-phenylene)bis(6-amino-2-(dicyanomethylene)-1,2-dihydropyridine-3,5-dicarbonitrile) (s) 39

^{13}C NMR spectrum of 4,4'-(1,4-phenylene)bis(6-amino-2-(dicyanomethylene)-1,2-dihydropyridine-3,5-dicarbonitrile) (s) 39

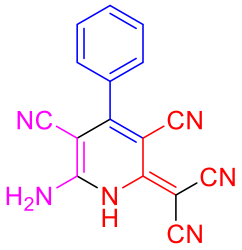

6-Amino-2-(dicyanomethylene)-4-phenyl-1,2-dihydropyridine-3,5-dicarbonitrile (a)

Yellow solid; Yield: 65%(0.185 g); Mp: 280-283 °C; IR (KBr): υ (cm^{-1}) = 3374, 3306, 3215, 2218, 2191, 1642. ^{1}H NMR (400 MHz, DMSO-d_6) δ 7.80 (s, 3H), 7.60 – 7.53 (m, 3H), 7.48 (d, J = 1.3 Hz, 2H). ^{13}C NMR (101 MHz, DMSO-d_6) δ 160.5, 160.4, 157.8, 135.3, 129.7, 128.4, 128.3, 116.2, 115.9, 85.4, 80.7, 43.6.

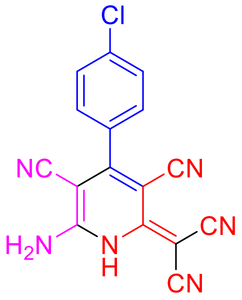

6-Amino-4-(4-chlorophenyl)-2-(dicyanomethylene)-1,2-dihydropyridine-3,5-dicarbonitrile (b)

Yellow solid; Yield: 87%(0.277 g); Mp: 290-262 °C; IR (KBr): υ (cm^{-1}) = 3363, 3302, 3211, 2228, 2217, 2191, 1645. ^{1}H NMR (600 MHz, DMSO-d_6) δ 7.59 (d, J = 8.4 Hz, 2H), 7.46 (d, J = 8.4 Hz, 2H), 6.52 (s, 3H).^{13}C NMR (151 MHz, DMSO-d_6) δ 161.6, 159.5, 158.5, 134.9, 134.9, 130.8, 129.1, 116.8, 116.5, 85.6, 81.0, 44.2.

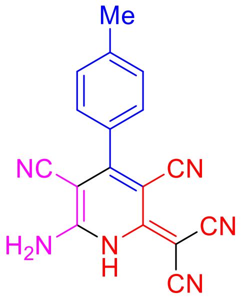

6-Amino-2-(dicyanomethylene)-4-(p-tolyl)-1,2-dihydropyridine-3,5-dicarbonitrile (c)

Yellow solid; Yield: 85%(0.253 g); Mp: >350 °C; IR (KBr): υ (cm^{-1}) = 3483, 3371, 3202, 2213, 2200, 2171, 1620. ^{1}H NMR (400 MHz, DMSO-d_6) δ 7.29 (s, 4H), 6.87 (s, 2H), 2.38 (s, 3H).. ^{13}C NMR (101 MHz, DMSO-d_6) δ 162.7, 159.7, 158.7, 139.0, 132.8, 128.9, 128.3, 116.9, 116.6, 85.0, 80.3, 20.9.

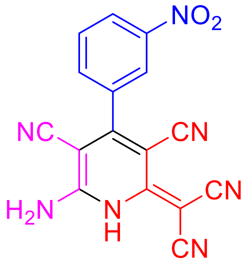

6-Amino-2-(dicyanomethylene)-4-(3-nitrophenyl)-1,2-dihydropyridine-3,5-dicarbonitrile (d)

Yellow solid; Yield: 77%(0.254 g); Mp: 316-318 °C; IR (KBr): υ (cm^{-1}) = 3438, 3339, 3213, 2197, 2181, 1657, 1556, 1352. ^{1}H NMR (600 MHz, DMSO-d_6) δ 8.38 – 8.35 (m, 1H), 8.33 – 8.30 (m, 1H), 7.93 (d, J = 7.7 Hz, 1H), 7.83 (t, J = 8.0 Hz, 1H), 7.05 (s, 2H). ^{13}C NMR (151 MHz, DMSO-d_6) δ 163.0, 159.0, 157.9, 148.0, 137.8, 135.9, 130.8, 124.8, 123.9, 117.1, 116.9, 85.3, 80.7, 44.3.

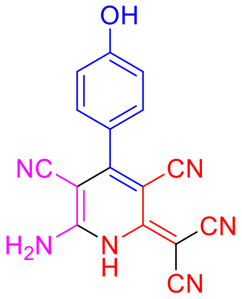

6-Amino-2-(dicyanomethylene)-4-(4-hydroxyphenyl)-1,2-dihydropyridine-3,5-dicarbonitrile (e)

Yellow solid; Yield: 82%(0.246 g); Mp: >350 °C; IR (KBr): υ (cm^{-1}) = 3342, 3227, 2183, 1652, 1553. ^{1}H NMR (600 MHz, DMSO-d_6) δ 9.85 (s, 1H), 7.23 (d, J = 8.5 Hz, 2H), 6.89 – 6.78 (m, 4H). ^{13}C NMR (151 MHz, DMSO-d_6) δ 163.4, 160.1, 159.3, 159.1, 130.6, 126.6, 117.7, 117.3, 115.5, 85.6, 80.7, 43.6. MS= 301

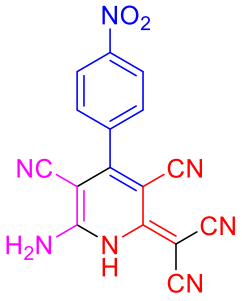

6-Amino-2-(dicyanomethylene)-4-(4-nitrophenyl)-1,2-dihydropyridine-3,5-dicarbonitrile (f)

Yellow solid; Yield: 79%(0.260 g); Mp: >350 °C; IR (KBr): υ (cm^{-1}) = 3336, 2196, 1655, 1556, 1508, 1360. ^{1}H NMR (600 MHz, DMSO-d_6) δ 8.35 (d, J = 8.7 Hz, 2H), 7.74 (d, J = 8.7 Hz, 2H), 7.05 (s, 2H). ^{13}C NMR (151 MHz, DMSO-d_6) δ 162.9, 159.0, 158.3, 148.5, 142.9, 130.7, 124.1, 116.9, 116.7, 85.0, 80.4, 44.3. MS = 330.

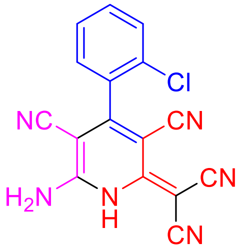

6-Amino-4-(2-chlorophenyl)-2-(dicyanomethylene)-1,2-dihydropyridine-3,5-dicarbonitrile (g)

Yellow solid; Yield: 68%(0.216 g); Mp: >350 °C; IR (KBr): υ (cm^{-1}) = 3438, 3342, 3231, 2195, 2164, 1636, 1557. ^{1}H NMR (600 MHz, DMSO-d_6) δ 7.61 (dd, J = 7.9, 1.3 Hz, 1H), 7.50 (td, J = 7.7, 1.9 Hz, 1H), 7.47 (td, J = 7.5, 1.4 Hz, 1H), 7.40 (dd, J = 7.4, 1.8 Hz, 1H), 7.00 (s, 2H). ^{13}C NMR (151 MHz, DMSO-d_6) δ 162.7, 158.9, 157.9, 135.5, 131.6, 131.4, 130.7, 130.0, 128.0, 116.6, 116.3, 86.0, 81.1, 44.0.

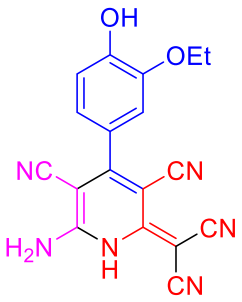

6-Amino-2-(dicyanomethylene)-4-(3-ethoxy-4-hydroxyphenyl)-1,2-dihydropyridine-3,5-dicarbonitrile (h)

Yellow solid; Yield: 83%(0.286 g); Mp: 333-335 °C; IR (KBr): υ (cm^{-1}) = 3431, 3342, 3236, 2198, 2165, 1649, 1554. ^{1}H NMR (600 MHz, DMSO-d_6) δ 9.38 (s, 1H), 6.97 – 6.94 (m, 1H), 6.87 (d, *J* = 8.1 Hz, 1H), 6.85 – 6.78 (m, 3H), 4.06 (q, *J* = 7.0 Hz, 2H), 1.36 – 1.33 (m, 3H). ^{13}C NMR (151 MHz, DMSO-d_6) δ 163.3, 160.1, 159.3, 148.6, 146.5, 126.8, 122.1, 117.8, 117.4, 115.7, 114.6, 85.6, 80.8, 64.3, 43.6, 15.1.

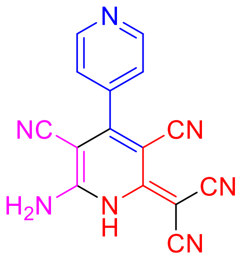

6-Amino-2-(dicyanomethylene)-1,2-dihydro-[4,4'-bipyridine]-3,5-dicarbonitrile (i)

Yellow solid; Yield: 74%(0.211 g); Mp: >350 °C; IR (KBr): υ (cm^{-1}) = 3443, 3345, 3224, 2211, 2195, 2175, 1650, 1569. ^{1}H NMR (600 MHz, DMSO-d_6) δ 8.72 (d, *J* = 5.7 Hz, 2H), 7.46 (d, *J* = 4.4 Hz, 2H), 7.04 (s, 2H). ^{13}C NMR (151 MHz, DMSO-d_6) δ 162.9, 159.0, 157.7, 150.3, 144.2, 123.6, 116.9, 116.6, 84.7, 80.1, 44.3.

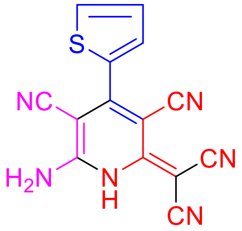

6-Amino-2-(dicyanomethylene)-4-(thiophen-2-yl)-1,2-dihydropyridine-3,5-dicarbonitrile (j)

Yellow solid; Yield: 70%(0.203 g); Mp: >350 °C; IR (KBr): υ (cm^{-1}) = 3481, 3345, 3211, 2213, 2189, 2163, 1624, 1555. ^{1}H NMR (600 MHz, DMSO-d_6) δ 7.82 (d, *J* = 4.9 Hz, 1H), 7.37 (d, *J* = 3.5 Hz, 1H), 7.23 – 7.18 (m, 1H), 6.97 (s, 2H). ^{13}C NMR (151 MHz, DMSO-d_6) δ 163.4, 159.3, 152.2, 135.2, 130.4, 129.6, 127.9, 117.3, 116.9, 85.8, 81.0, 44.2.

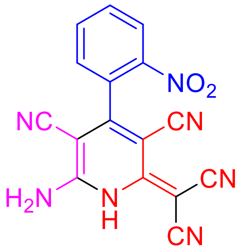

6-Amino-2-(dicyanomethylene)-4-(2-nitrophenyl)-1,2-dihydropyridine-3,5-dicarbonitrile (k)

Yellow solid; Yield: 60%(0.197 g); Mp: >350 °C; IR (KBr): υ (cm^{-1}) = 3437, 3341, 3230, 2218, 2195, 1637, 1560, 1517, 1351. ^{1}H NMR (600 MHz, DMSO-d_6) δ 8.27 (d, *J* = 8.2 Hz, 1H), 7.92 (t, *J* = 7.5 Hz, 1H), 7.80 (t, *J* = 7.9 Hz, 1H), 7.60 (d, *J* = 7.5 Hz,

1H), 7.05 (s, 2H). ^{13}C NMR (151 MHz, DMSO-d_6) δ 170.8, 162.6, 158.8, 158.1, 147.3, 135.2, 131.8, 131.6, 131.3, 125.4, 116.6, 116.4, 84.9, 80.2, 60.2, 44.1, 21.2, 14.6.

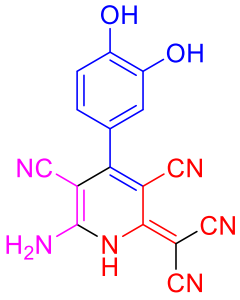

6-Amino-2-(dicyanomethylene)-4-(3,4-dihydroxyphenyl)-1,2-dihydropyridine-3,5-dicarbonitrile (l)

Yellow solid; Yield: 75%(0.237 g); Mp: >350 °C; IR (KBr): υ (cm^{-1}) = 3457, 3334, 3225, 2197, 2168, 1650, 1560. ^1H NMR (600 MHz, DMSO-d_6) δ 9.33 (s, 1H), 9.26 (s, 1H), 6.80 (d, J = 8.0 Hz, 3H), 6.77 (s, 1H), 6.67 (d, J = 8.1 Hz, 1H).

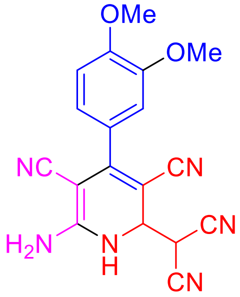

6-Amino-2-(dicyanomethyl)-4-(3,4-dimethoxyphenyl)-1,2-dihydropyridine-3,5-dicarbonitrile (m)

Yellow solid; Yield: 70%(0.242 g); Mp: 332-335 °C; IR (KBr): υ (cm^{-1}) = 3504, 3489, 3374, 2216, 2194, 2172, 1613, 1551. ^1H NMR (400 MHz, DMSO-d_6) δ 7.07 (d, J = 8.3 Hz, 1H), 7.03 (d, J = 2.1 Hz, 1H), 6.97 (dd, J = 8.2, 2.1 Hz, 1H), 6.88 (s, 2H), 3.82 (s, 3H), 3.79 (s, 3H). ^{13}C NMR (101 MHz, DMSO-d_6) δ 162.7, 159.3, 158.8, 149.6, 148.0, 127.7, 121.3, 117.1, 116.7, 112.2, 111.2, 85.1, 80.3, 55.5, 55.4, 43.1, 40.0,

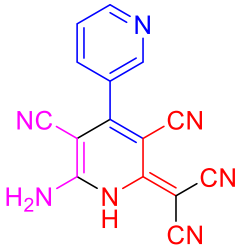

6'-Amino-2'-(dicyanomethylene)-1',2'-dihydro-[3,4'-bipyridine]-3',5'-dicarbonitrile (n)

Brown solid; Yield: 80%(0.228 g); Mp: >350 °C; IR (KBr): υ (cm^{-1}) = 3389, 3317, 3161, 2209, 2189, 2156, 1654, 1577, 1514. ^1H NMR (400 MHz, DMSO-d_6) δ 8.79 – 8.47 (m, 2H), 7.91 (dt, J = 7.9, 2.0 Hz, 1H), 7.55 (dd, J = 7.9, 4.9 Hz, 1H), 7.03 (s, 2H). ^{13}C NMR (101 MHz, DMSO-d_6) δ 162.52, 158.57, 156.38, 150.38, 148.47, 136.39, 131.89, 123.40, 116.65, 116.43, 85.08, 80.40, 40.03.

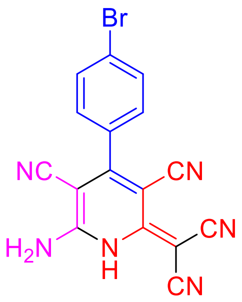

6-Amino-4-(4-bromophenyl)-2-(dicyanomethylene)-1,2-dihydropyridine-3,5-dicarbonitrile (o)

White solid; Yield: 84%(0.305 g); Mp: 330-332 °C; IR (KBr): υ (cm^{-1}) = 3451, 3339, 3224, 2214, 2194, 2170, 1674, 1572, 1511. ^{1}H NMR (400 MHz, DMSO-d_6) δ 7.77 – 7.64 (m, 2H), 7.46 – 7.30 (m, 2H), 6.97 (s, 2H). ^{13}C NMR (101 MHz, DMSO-d_6) δ 162.5, 158.5, 158.5, 135.0, 131.4, 130.6, 123.0, 116.7, 116.4, 84.8, 80.1, 40.0.

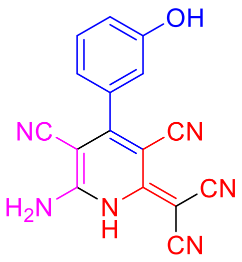

6-Amino-2-(dicyanomethylene)-4-(3-hydroxyphenyl)-1,2-dihydropyridine-3,5-dicarbonitrile (p)

Yellow solid; Yield: 85%(0.255 g); Mp: >350 °C; IR (KBr): υ (cm^{-1}) = 3476, 3330, 3218, 22022171, 1625, 1548. ^{1}H NMR (600 MHz, DMSO-d_6) δ 9.72 (s, 1H), 7.28 (t, J = 7.8 Hz, 1H), 6.94 – 6.84 (m, 3H), 6.77 (d, J = 7.5 Hz, 1H), 6.73 (s, 1H).

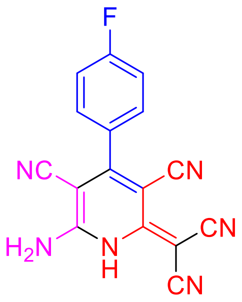

6-Amino-2-(dicyanomethylene)-4-(4-fluorophenyl)-1,2-dihydropyridine-3,5-dicarbonitrile (q)

Yellow solid; Yield: 83%(0.251 g); Mp: 324-326 °C; IR (KBr): υ (cm^{-1}) = 3449, 3374, 3309, 3216, 2212, 2194, 2171, 1646. ^{1}H NMR (600 MHz, DMSO-d_6) δ 7.51 – 7.45 (m, 2H), 7.34 (t, J = 8.6 Hz, 2H), 6.97 (s, 2H).

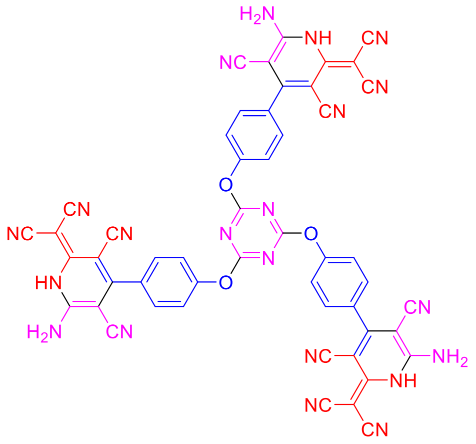

4,4',4''-(((1,3,5-Triazine-2,4,6-triyl)tris(oxy))tris(benzene-4,1-diyl))tris(6-amino-2-(dicyanomethylene)-1,2-dihydropyridine-3,5-dicarbonitrile) (r)

Yellow solid; Yield: 65%(0.634 g); Mp: >350 °C; IR (KBr): υ (cm^{-1}) = 3504, 3374, 3224, 2215, 2194, 2166, 1613, 1551. ^{1}H NMR (400 MHz, DMSO-d_6) δ 7.53 (td, J = 8.5, 4.3 Hz, 1H), 7.49 – 7.41 (m, 2H), 7.38 (d, J = 8.6 Hz, 1H), 6.95 (s, 1H), 6.91 (s, 1H). ^{13}C NMR (101 MHz, DMSO-d_6) δ 173.3, 163.2, 159.2, 159.1, 153.2, 152.5, 133.9, 132.5, 130.6, 130.1, 122.0, 121.7, 120.6, 117.3, 117.0, 85.5, 80.8, 40.5, 40.3, 40.1.

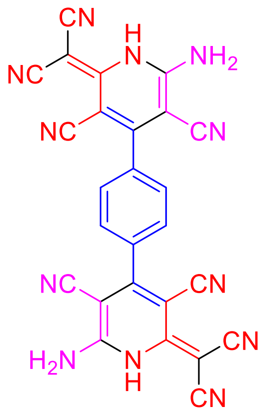

4,4'-(1,4-Phenylene)bis(6-amino-2-(dicyanomethylene)-1,2-dihydropyridine-3,5-dicarbonitrile) (s)

Yellow solid; Yield: 77%(0.377 g); Mp: >350 °C; IR (KBr): υ (cm^{-1}) = 3395, 3334, 3228, 2192, 2158, 1650, 1549, 1429. ^{1}H NMR (400 MHz, DMSO-d_6) δ 7.55 (s, 1H), 6.94 (s, 1H). ^{13}C NMR (101 MHz, DMSO-d_6) δ 159.2, 144.3, 139.6, 135.2, 135.0, 134.8, 132.4, 131.5, 129.1, 128.9, 126.5, 126.4, 121.7, 121.2, 120.1, 119.6, 118.4, 116.8, 111.1, 111.0, 110.8, 102.8, 42.3.

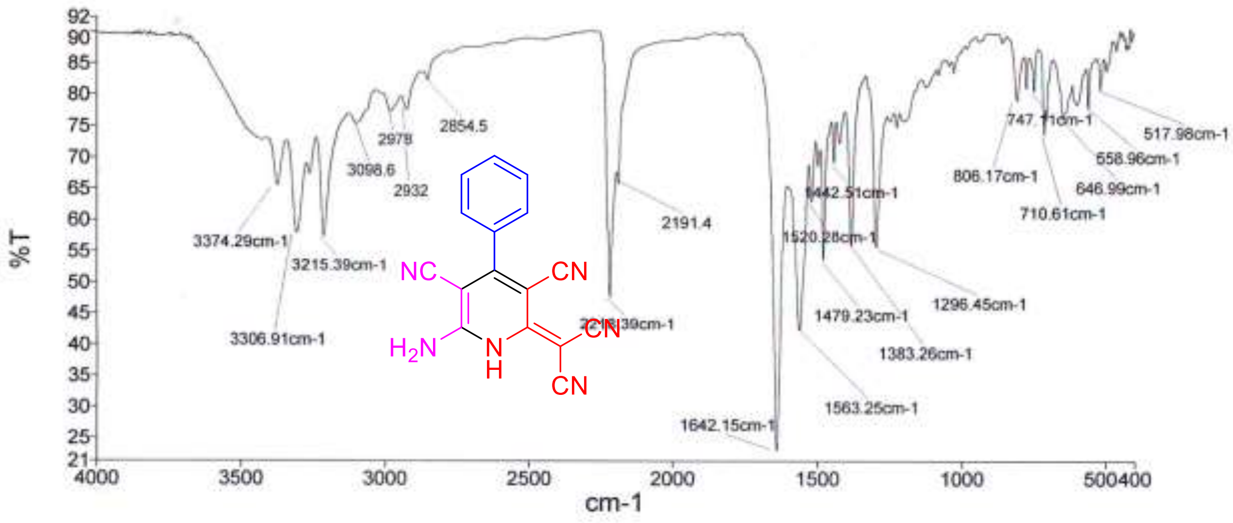

FT-IR spectrum of 6-amino-2-(dicyanomethylene)-4-phenyl-1,2-dihydropyridine-3,5-dicarbonitrile (a)

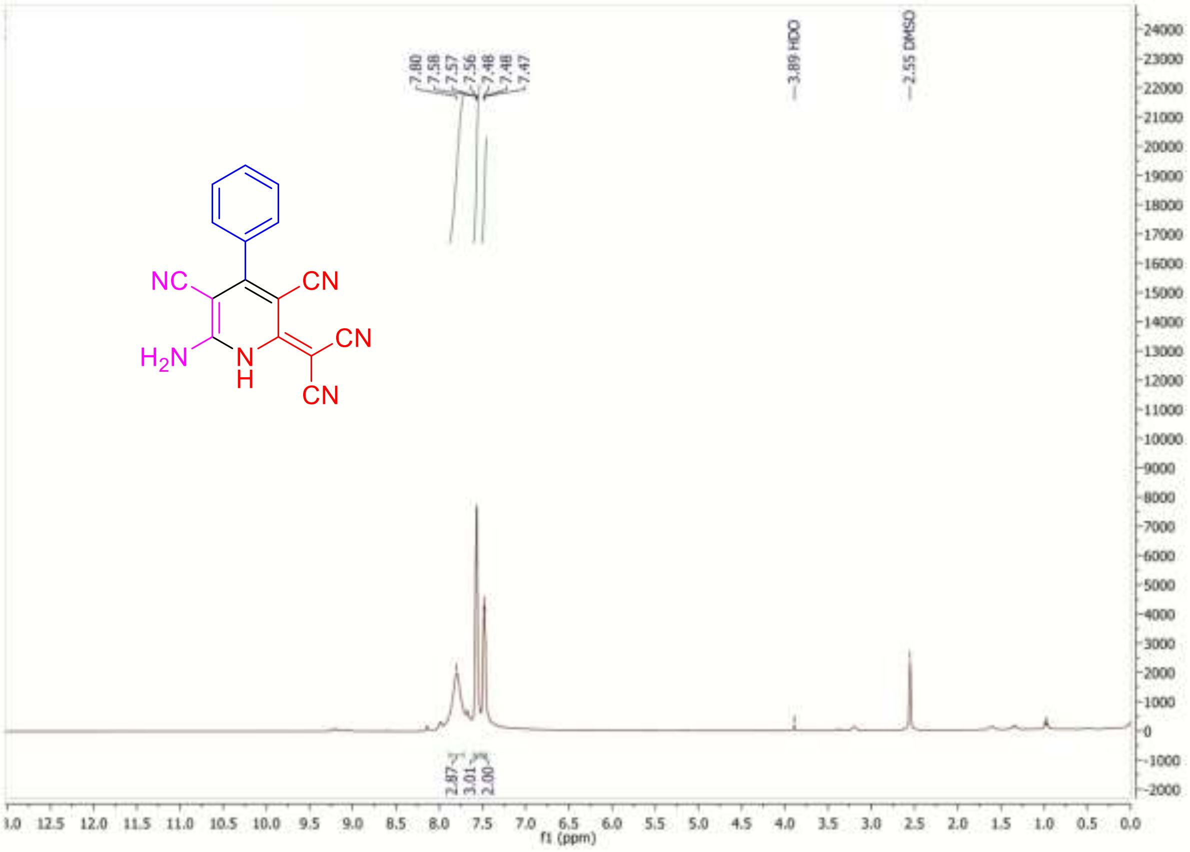

^{1}H NMR spectrum of 6-Amino-2-(dicyanomethylene)-4-phenyl-1,2-dihydropyridine-3,5-dicarbonitrile(a)

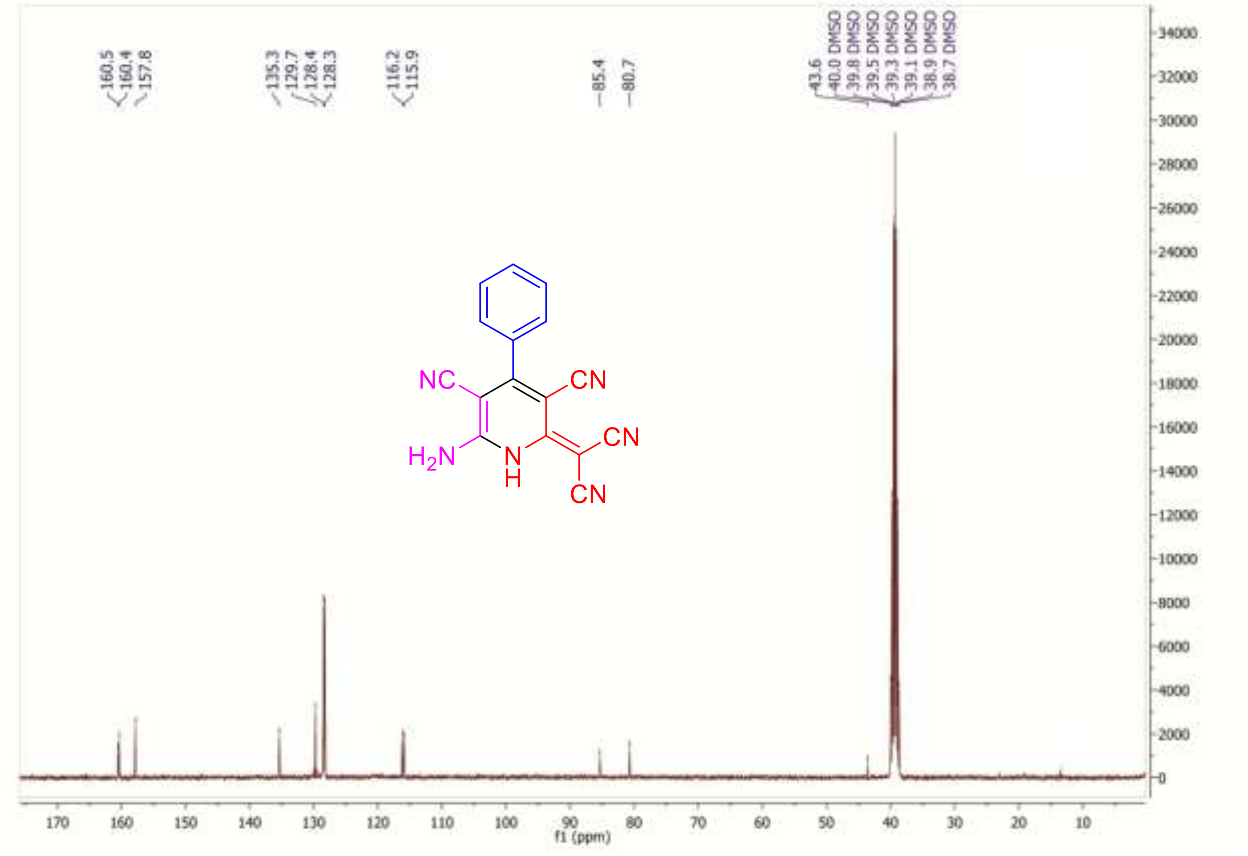

6-Amino-2-(dicyanomethylene)-4-phenyl-1,2-dihydropyridine-3,5-dicarbonitrile(a)

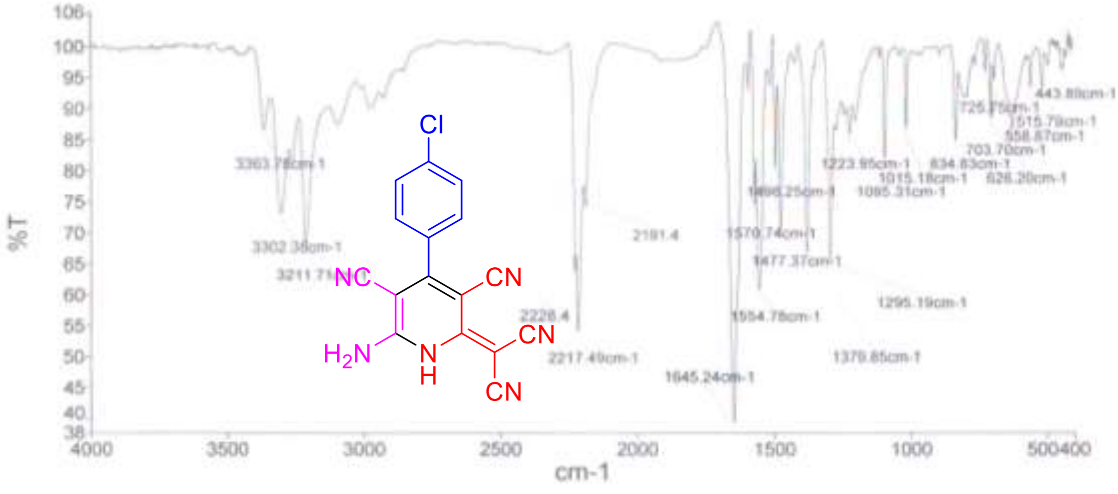

FT-IR spectrum of 6-amino-4-(4-chlorophenyl)-2-(dicyanomethylene)-1,2-dihydropyridine-3,5-dicarbonitrile (b)

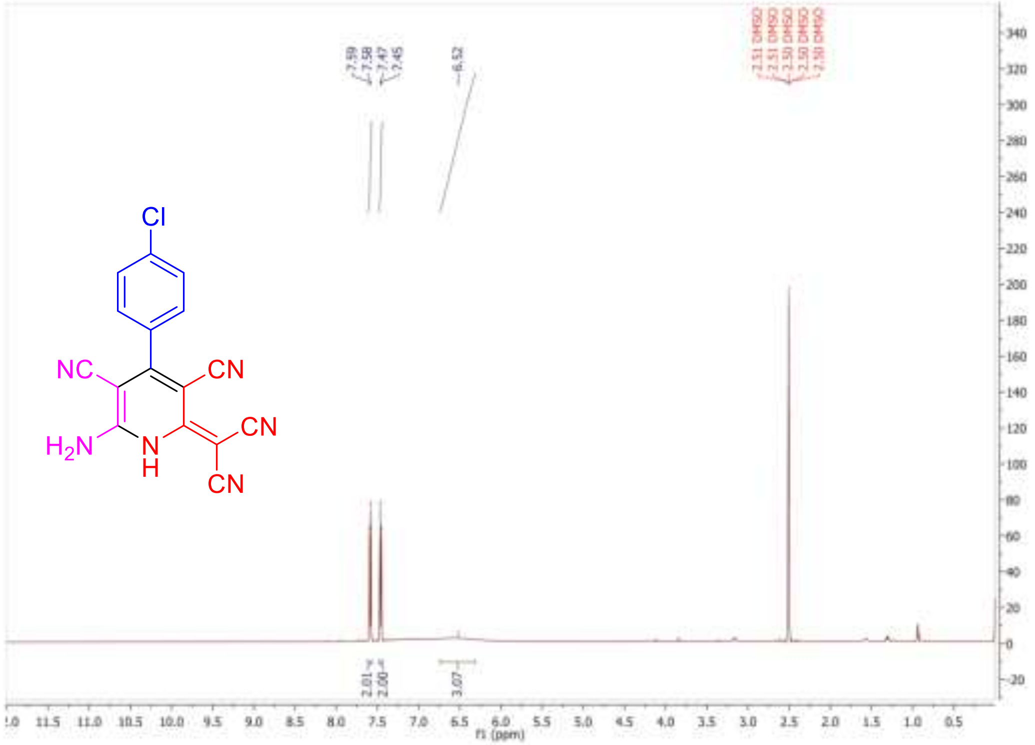

^{1}H NMR spectrum of 6-amino-4-(4-chlorophenyl)-2-(dicyanomethylene)-1,2-dihydropyridine-3,5-dicarbonitrile (b)

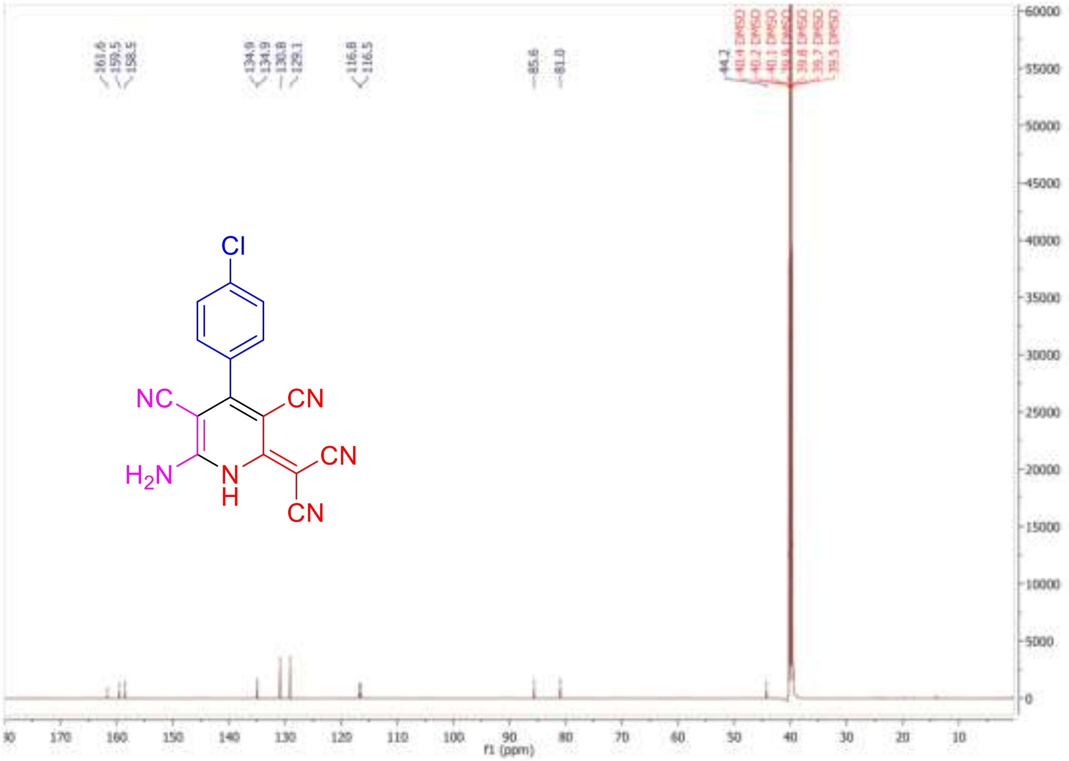

^{13}C NMR spectrum of 6-amino-4-(4-chlorophenyl)-2-(dicyanomethylene)-1,2-dihydropyridine-3,5-dicarbonitrile (b)

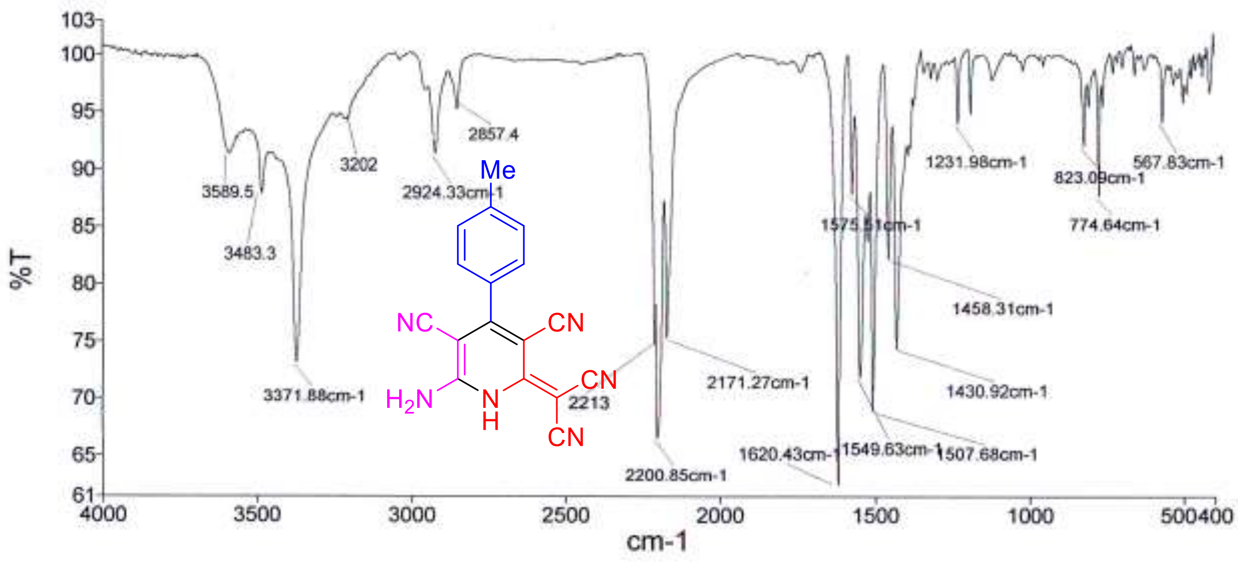

FT-IR spectrum of 6-amino-2-(dicyanomethylene)-4-(p-tolyl)-1,2-dihydropyridine-3,5-dicarbonitrile (c)

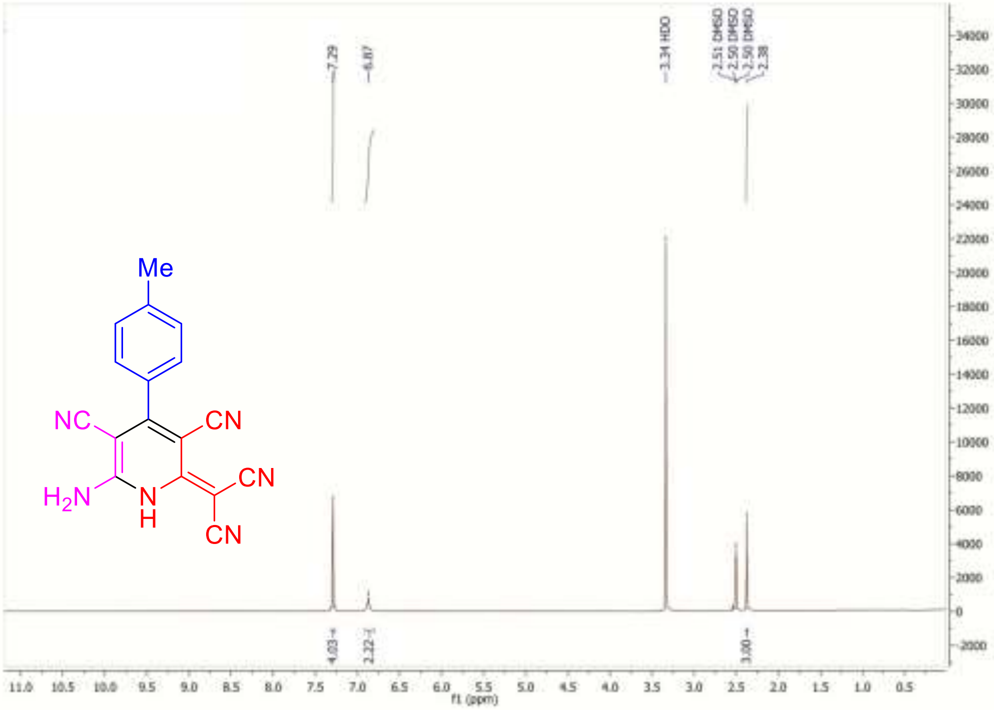

^{1}H NMR spectrum of 6-Amino-2-(dicyanomethylene)-4-(p-tolyl)-1,2-dihydropyridine-3,5-dicarbonitrile (c)

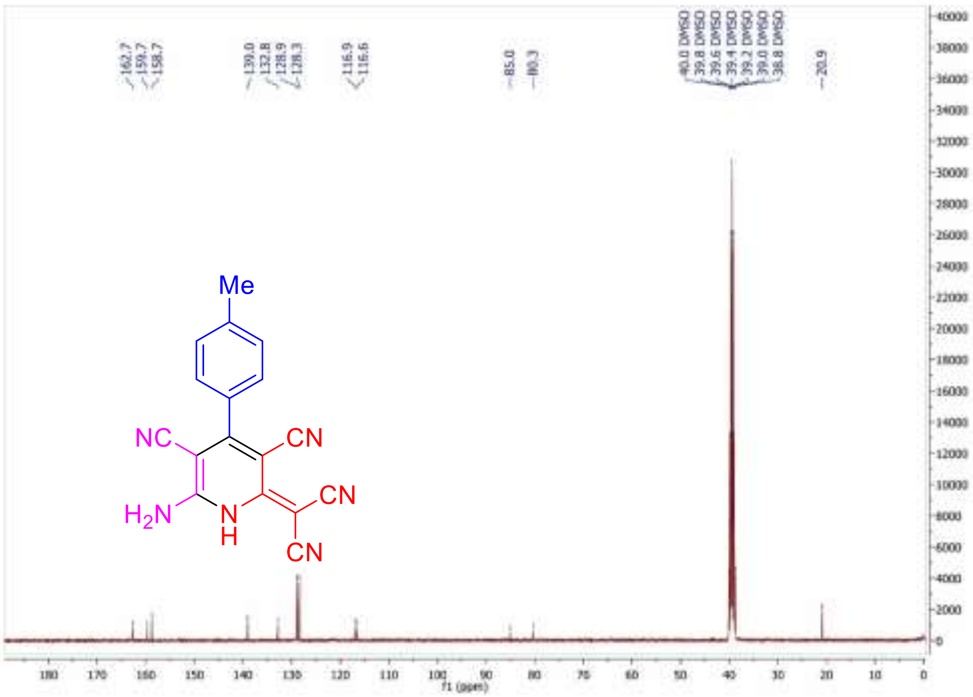

^{13}C NMR spectrum of 6-Amino-2-(dicyanomethylene)-4-(p-tolyl)-1,2-dihydropyridine-3,5-dicarbonitrile (c)

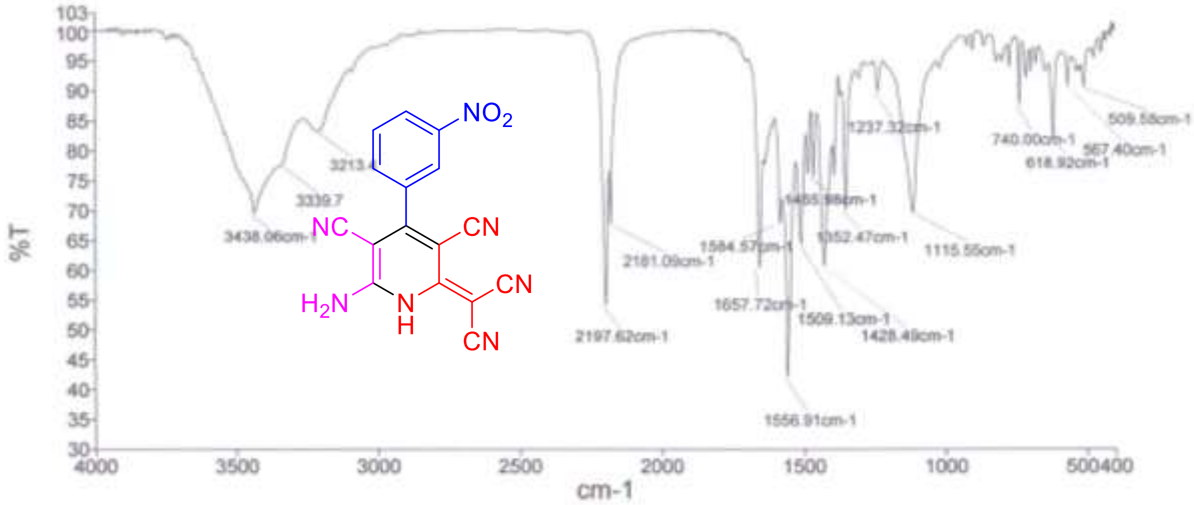

FT-IR spectrum of 6-amino-2-(dicyanomethylene)-4-(3-nitrophenyl)-1,2-dihydropyridine-3,5-dicarbonitrile (d)

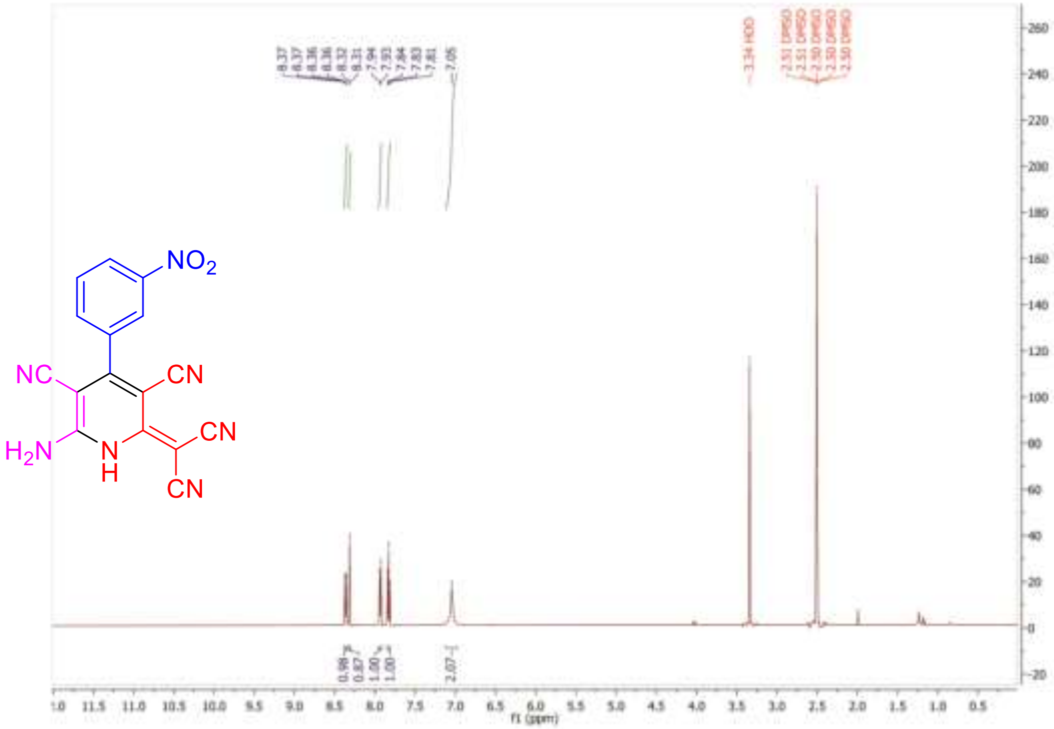

^{1}H NMR spectrum of 6-amino-2-(dicyanomethylene)-4-(3-nitrophenyl)-1,2-dihydropyridine-3,5-dicarbonitrile (d)

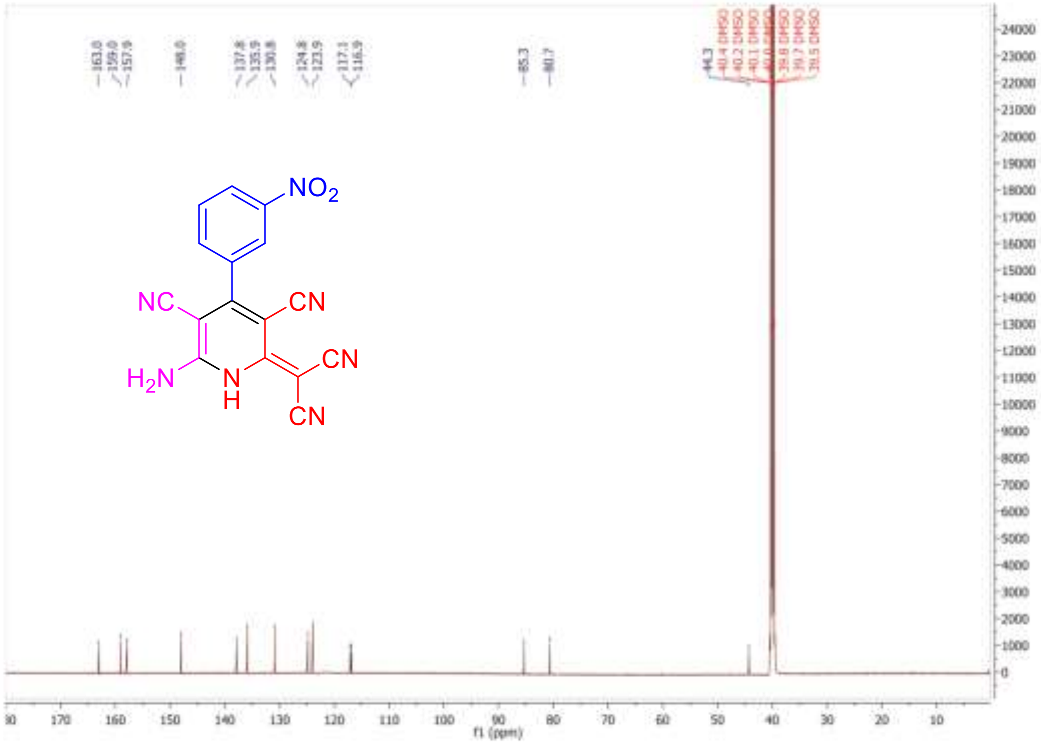

^{13}C NMR spectrum of 6-amino-2-(dicyanomethylene)-4-(3-nitrophenyl)-1,2-dihydropyridine-3,5-dicarbonitrile (d)

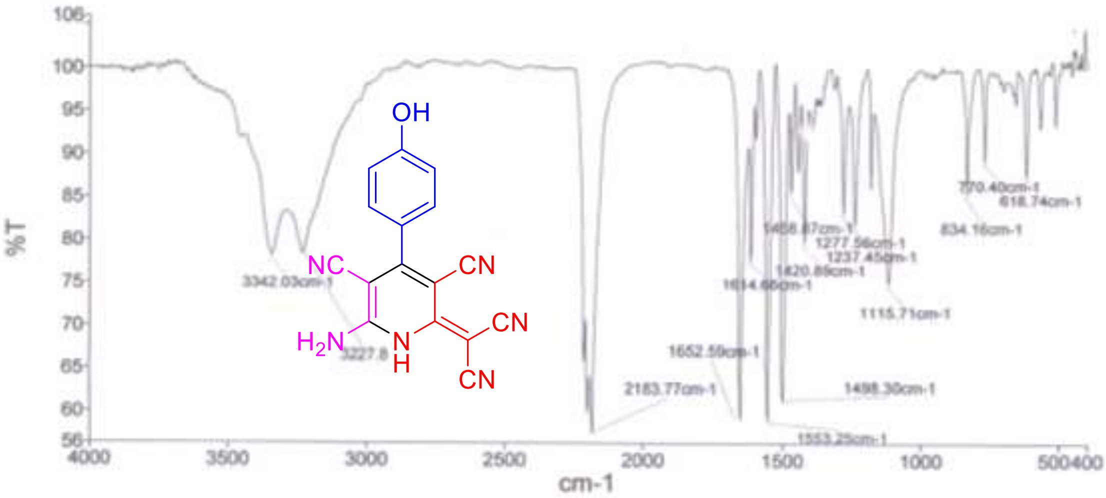

FT-IR spectrum of 6-amino-2-(dicyanomethylene)-4-(4-hydroxyphenyl)-1,2-dihydropyridine-3,5-dicarbonitrile (e)

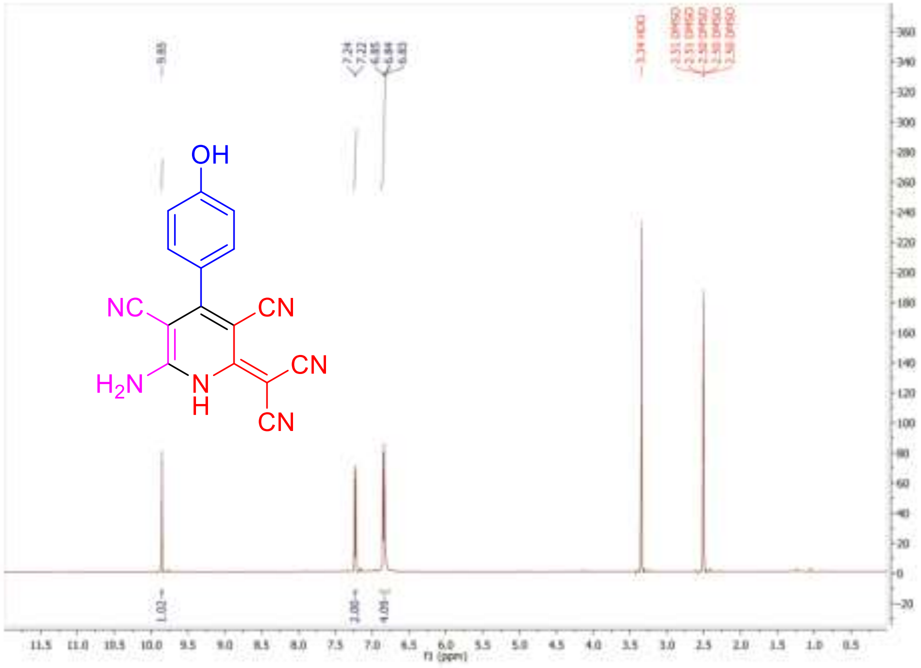

^{1}H NMR spectrum of 6-amino-2-(dicyanomethylene)-4-(4-hydroxyphenyl)-1,2-dihydropyridine-3,5-dicarbonitrile (e)

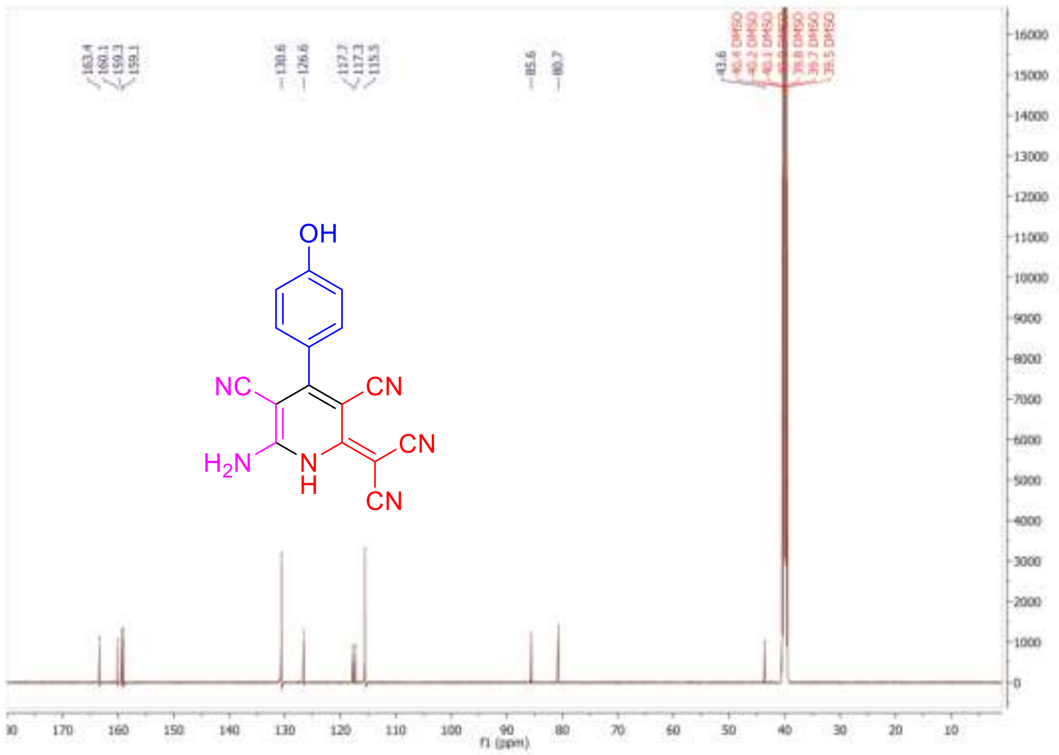

^{13}C NMR spectrum of 6-amino-2-(dicyanomethylene)-4-(4-hydroxyphenyl)-1,2-dihydropyridine-3,5-dicarbonitrile (e)

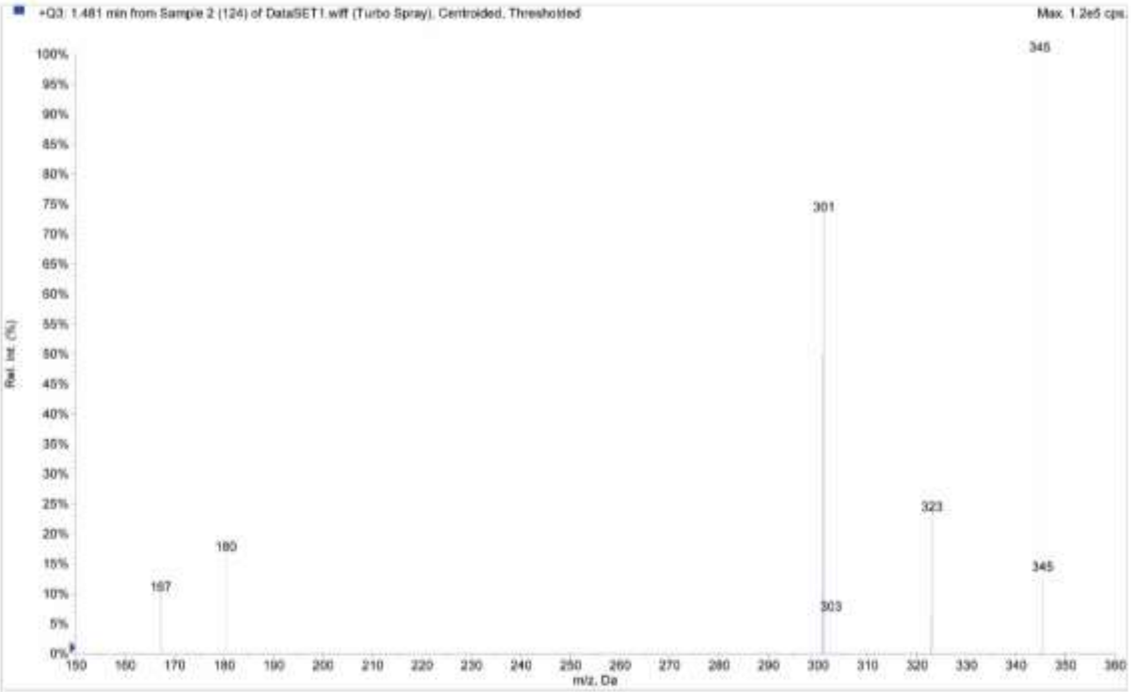

Mass spectrum of 6-amino-2-(dicyanomethylene)-4-(4-hydroxyphenyl)-1,2-dihydropyridine-3,5-dicarbonitrile (e)

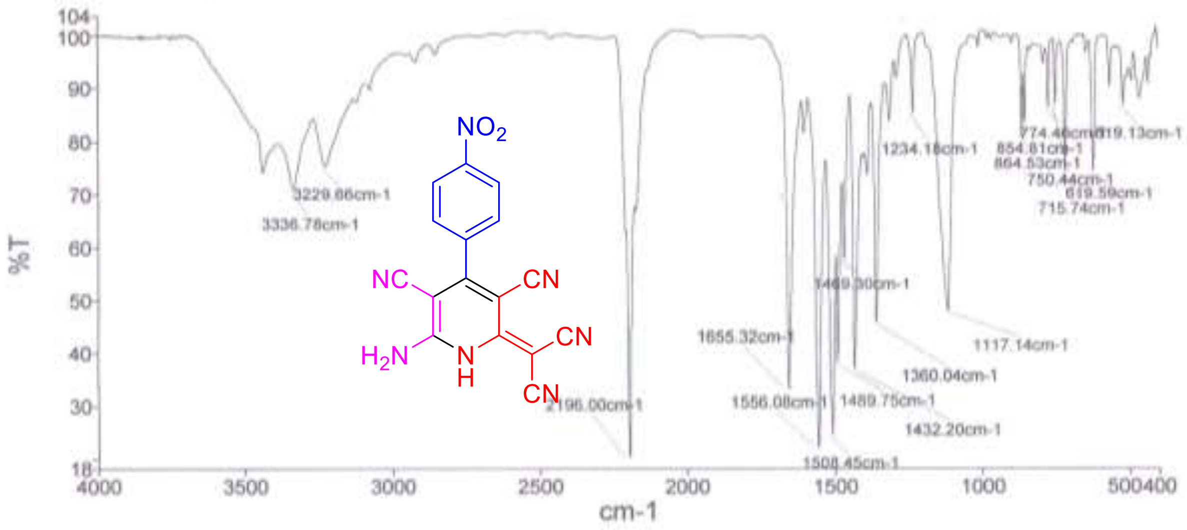

FT-IR spectrum of 6-amino-2-(dicyanomethylene)-4-(4-nitrophenyl)-1,2-dihydropyridine-3,5-dicarbonitrile (f)

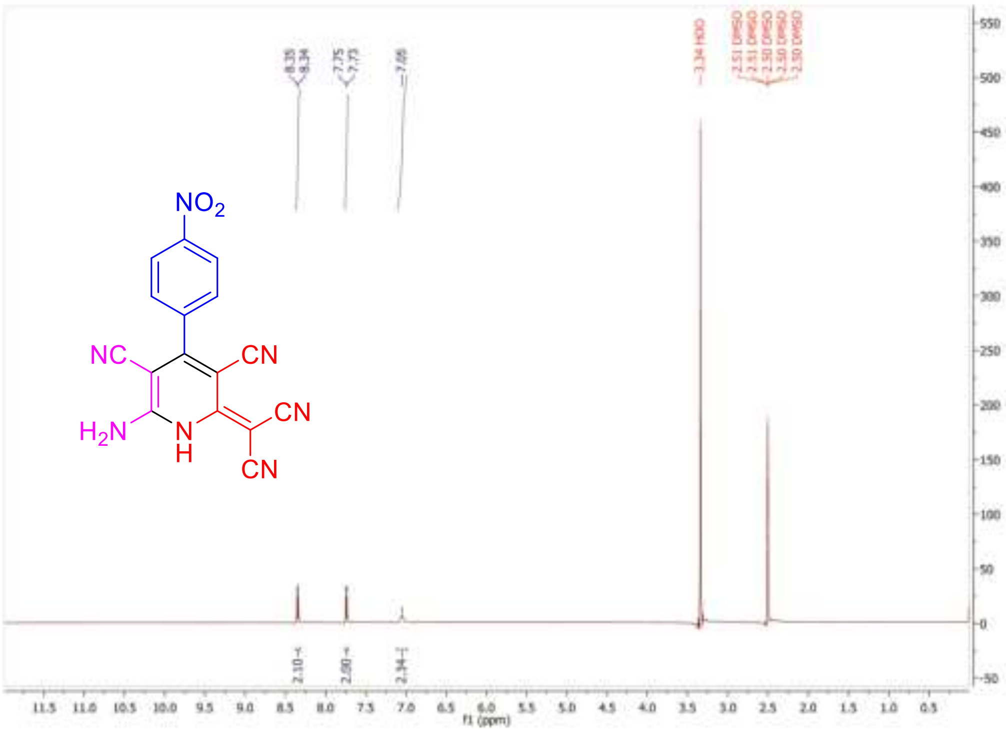

^{1}H NMR spectrum of 6-amino-2-(dicyanomethylene)-4-(4-nitrophenyl)-1,2-dihydropyridine-3,5-dicarbonitrile (f)

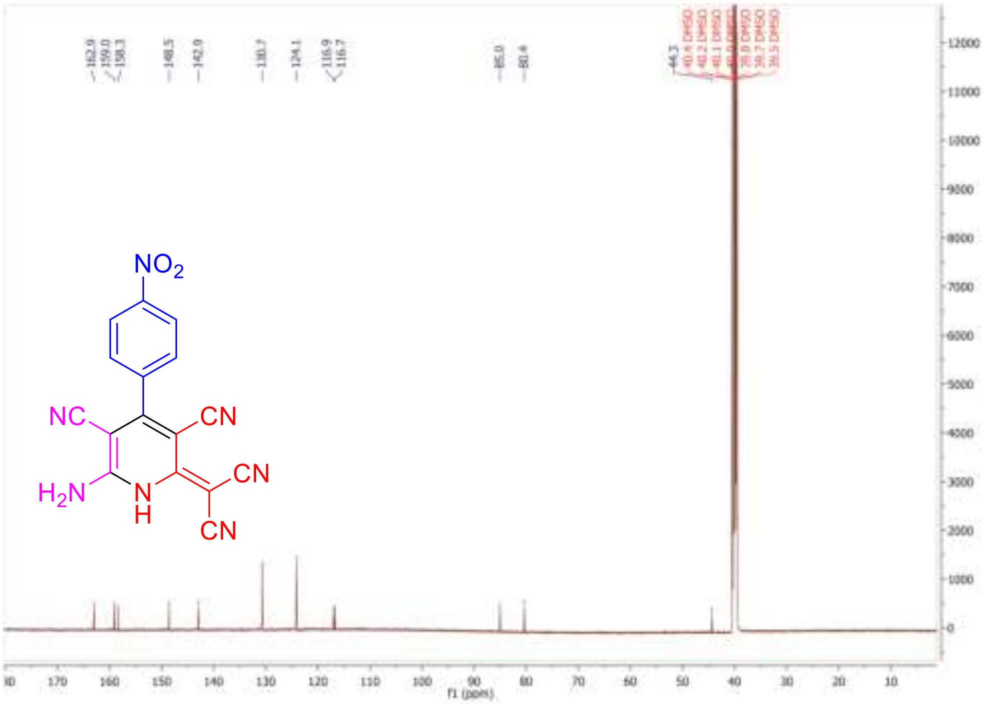

^{13}C NMR spectrum of 6-amino-2-(dicyanomethylene)-4-(4-nitrophenyl)-1,2-dihydropyridine-3,5-dicarbonitrile (f)

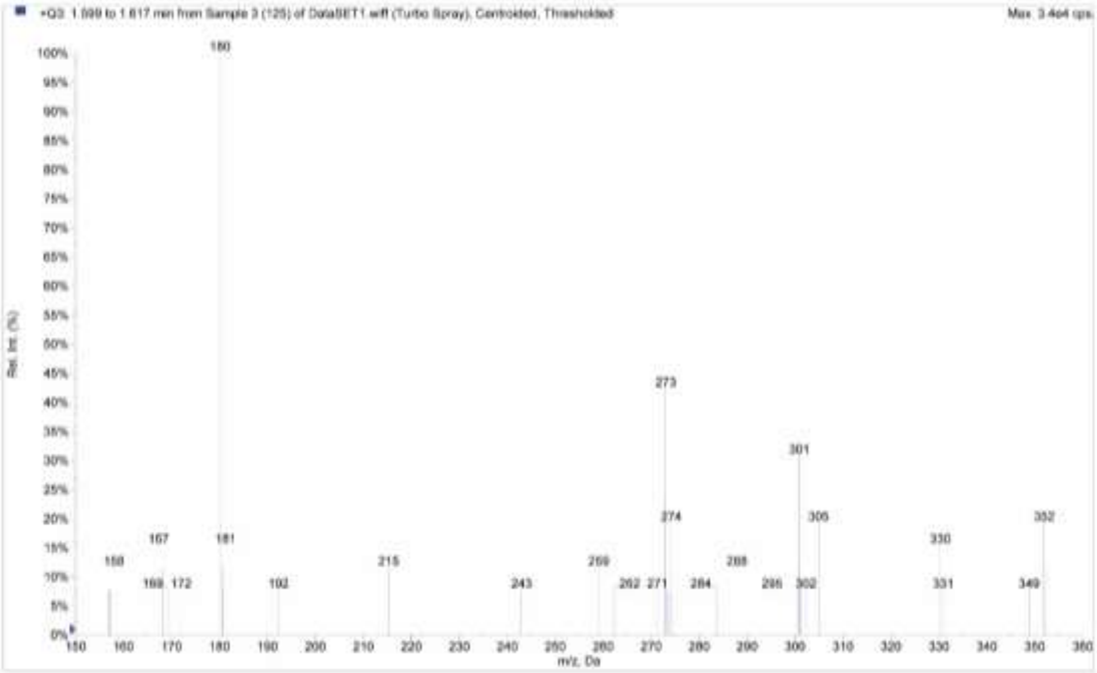

Mass spectrum of 6-amino-2-(dicyanomethylene)-4-(4-nitrophenyl)-1,2-dihydropyridine-3,5-dicarbonitrile (f)

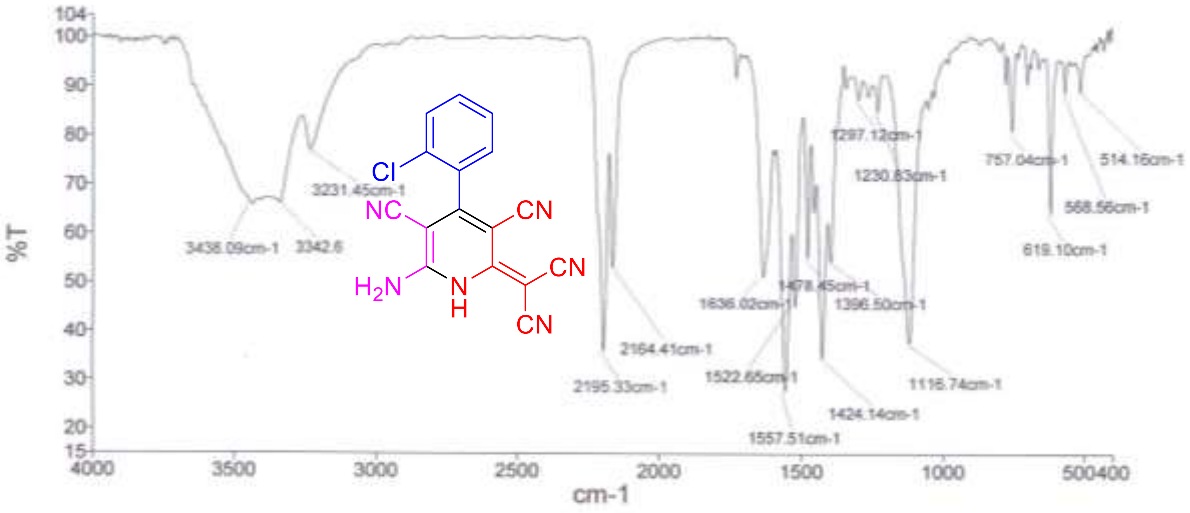

FT-IR spectrum of 6-amino-4-(2-chlorophenyl)-2-(dicyanomethylene)-1,2-dihydropyridine-3,5-dicarbonitrile (g)

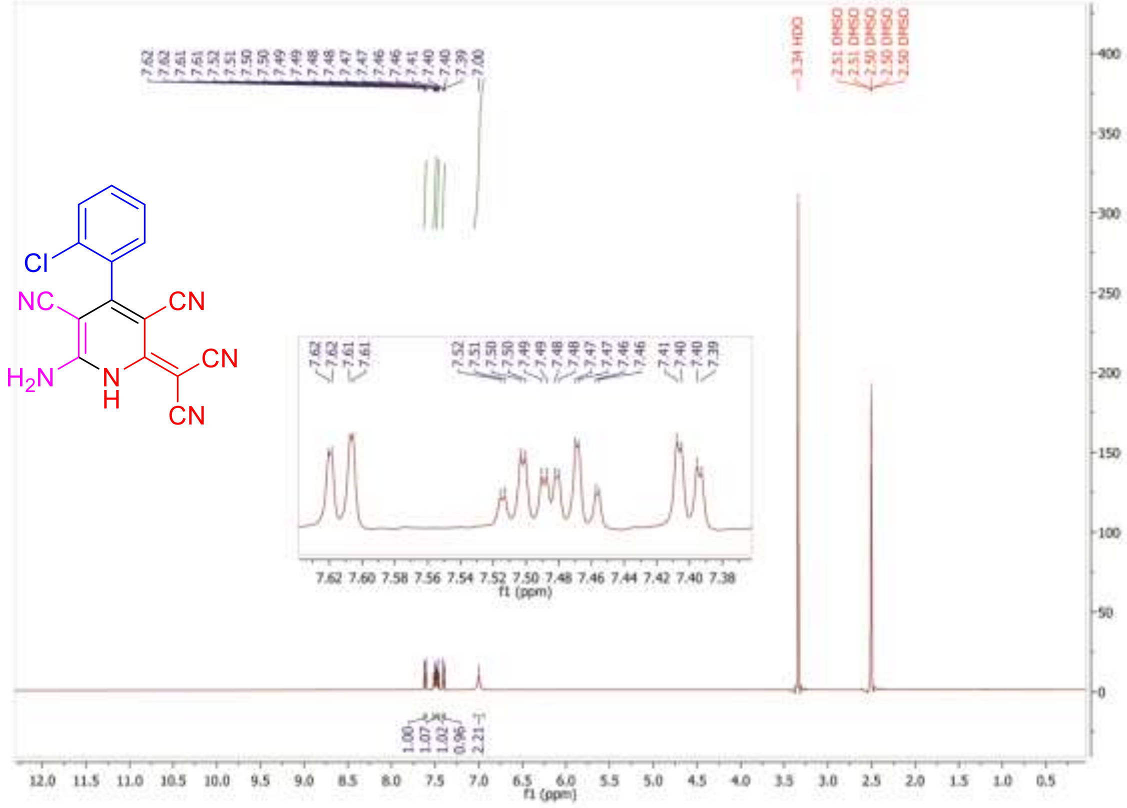

^{1}H NMR spectrum of 6-amino-4-(2-chlorophenyl)-2-(dicyanomethylene)-1,2-dihydropyridine-3,5-dicarbonitrile (g)

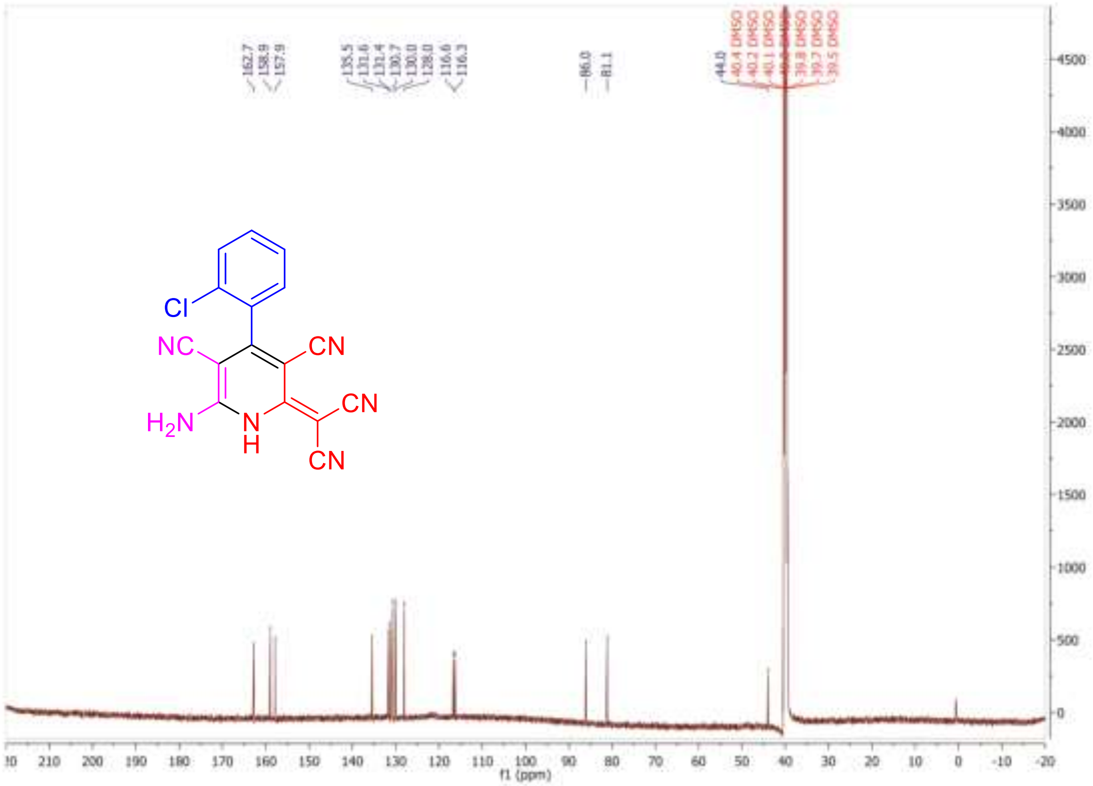

^{13}C NMR spectrum of 6-amino-4-(2-chlorophenyl)-2-(dicyanomethylene)-1,2-dihydropyridine-3,5-dicarbonitrile (g)

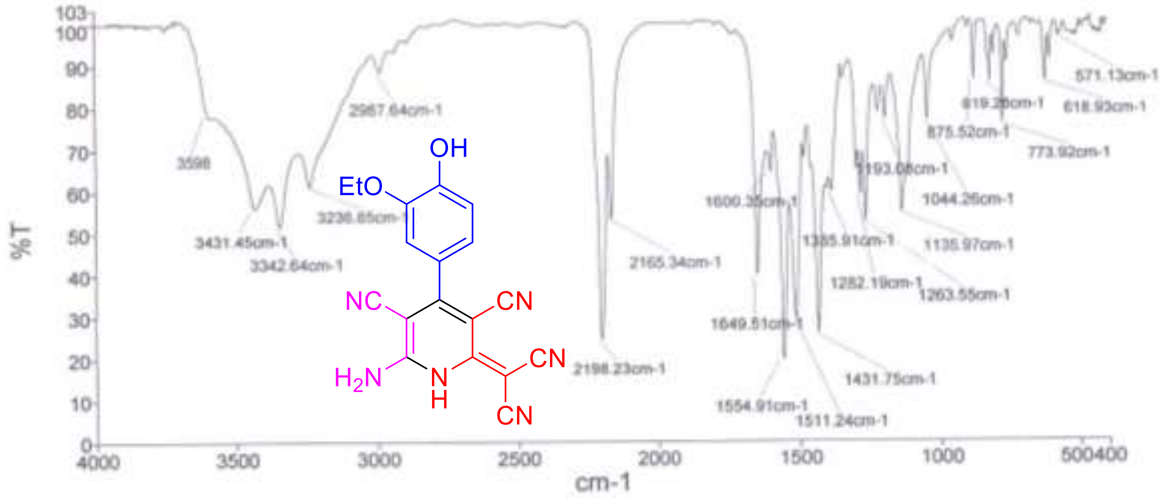

FT-IR spectrum of 6-amino-2-(dicyanomethylene)-4-(3-ethoxy-4-hydroxyphenyl)-1,2-dihydropyridine-3,5-dicarbonitrile (h)

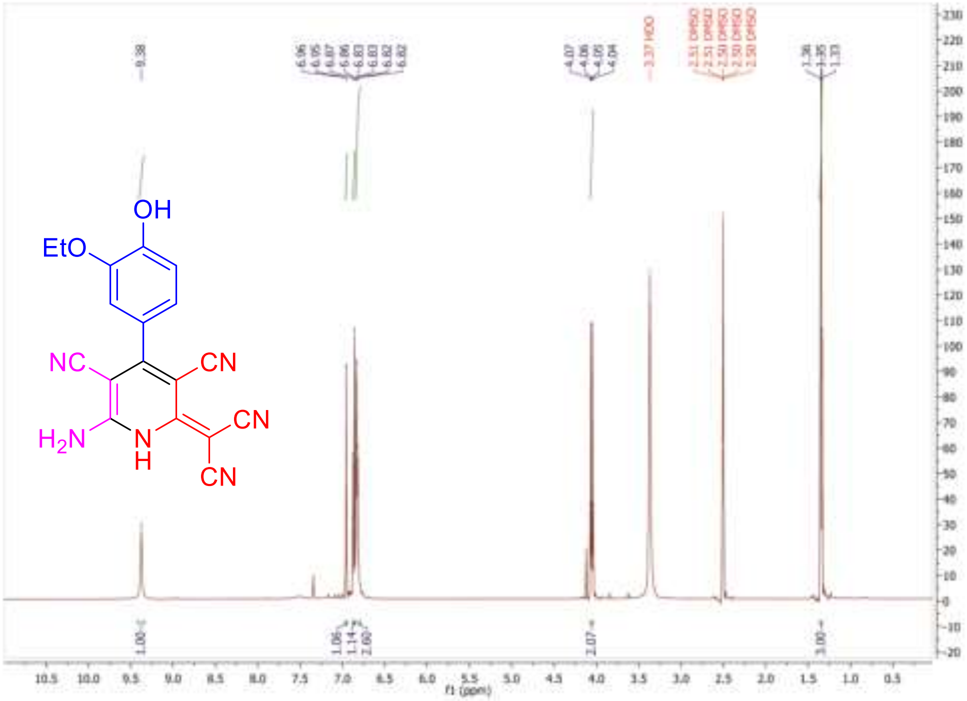

^{1}H NMR spectrum of 6-amino-2-(dicyanomethylene)-4-(3-ethoxy-4-hydroxyphenyl)-1,2-dihydropyridine-3,5-dicarbonitrile (h)

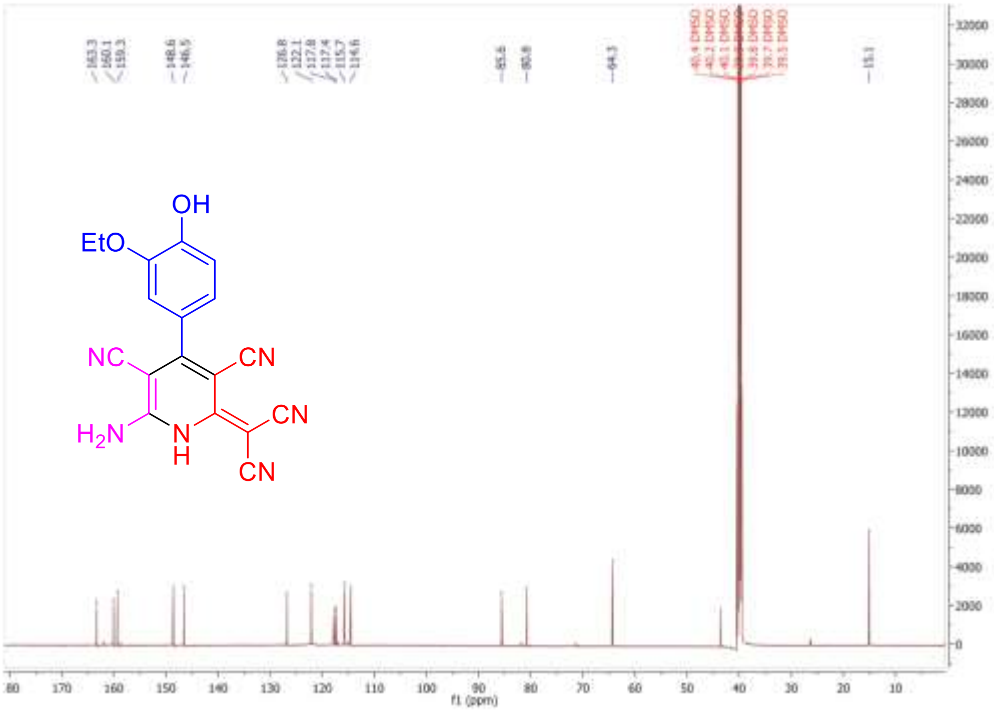

^{13}C NMR spectrum of 6-amino-2-(dicyanomethylene)-4-(3-ethoxy-4-hydroxyphenyl)-1,2-dihydropyridine-3,5-dicarbonitrile (h)

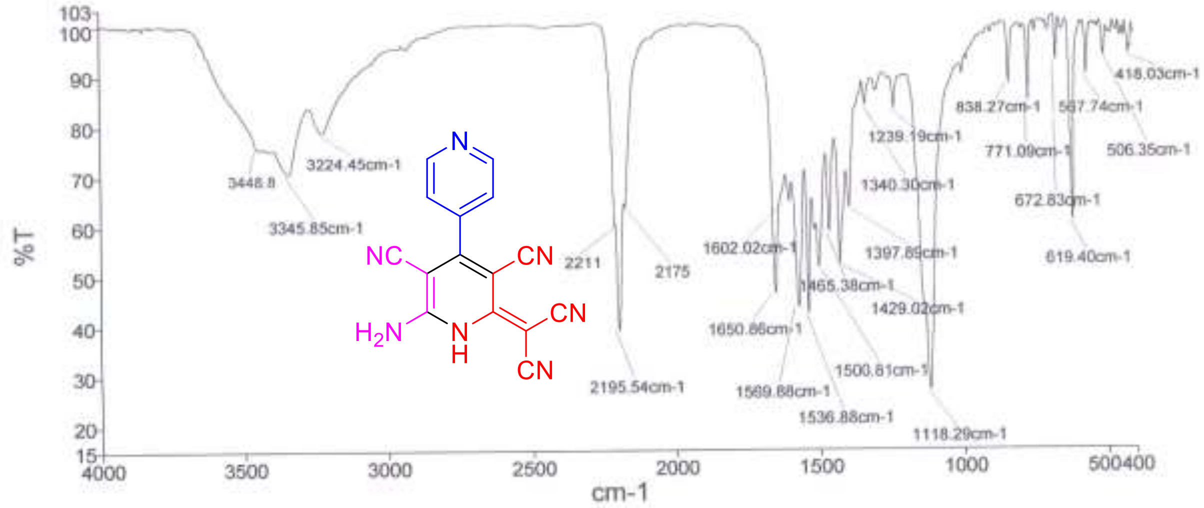

FT-IR spectrum of 6-amino-2-(dicyanomethylene)-1,2-dihydro-[4,4'-bipyridine]-3,5-dicarbonitrile (i)

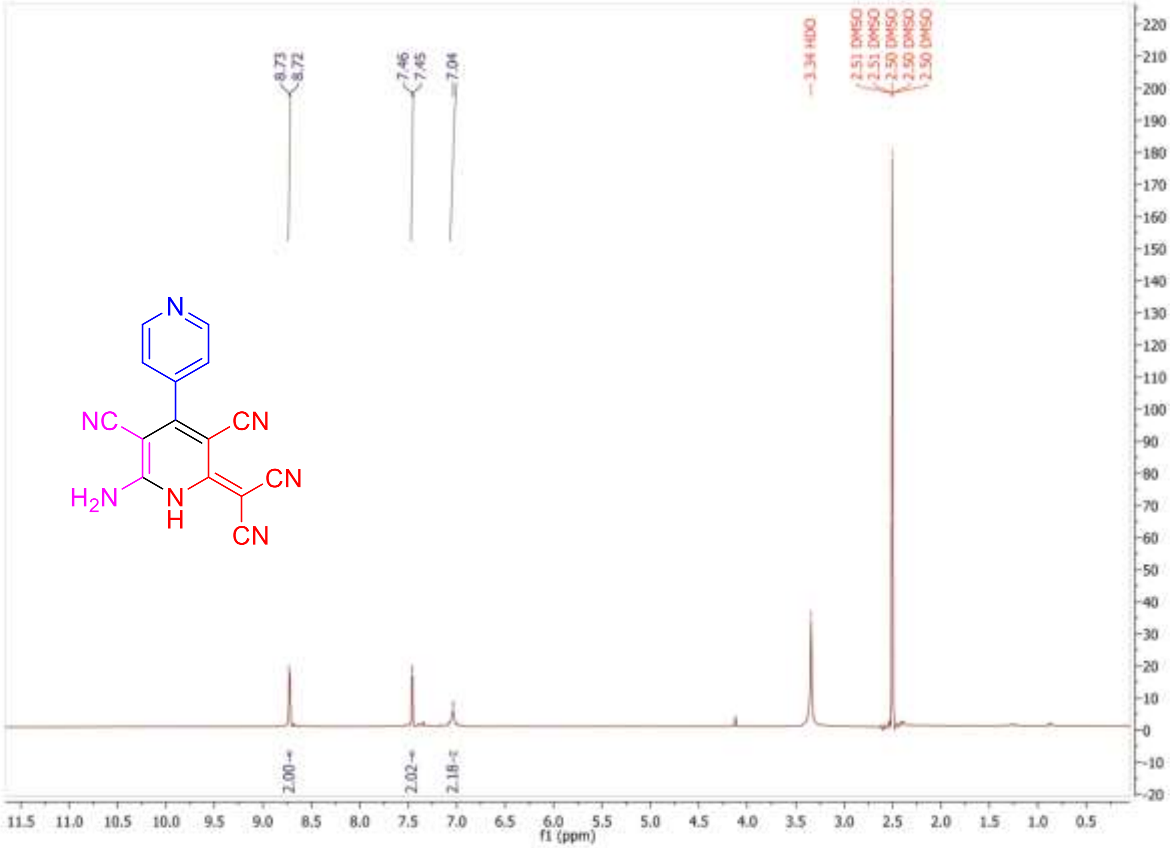

^{1}H NMR spectrum of 6-amino-2-(dicyanomethylene)-1,2-dihydro-[4,4'-bipyridine]-3,5-dicarbonitrile (i)

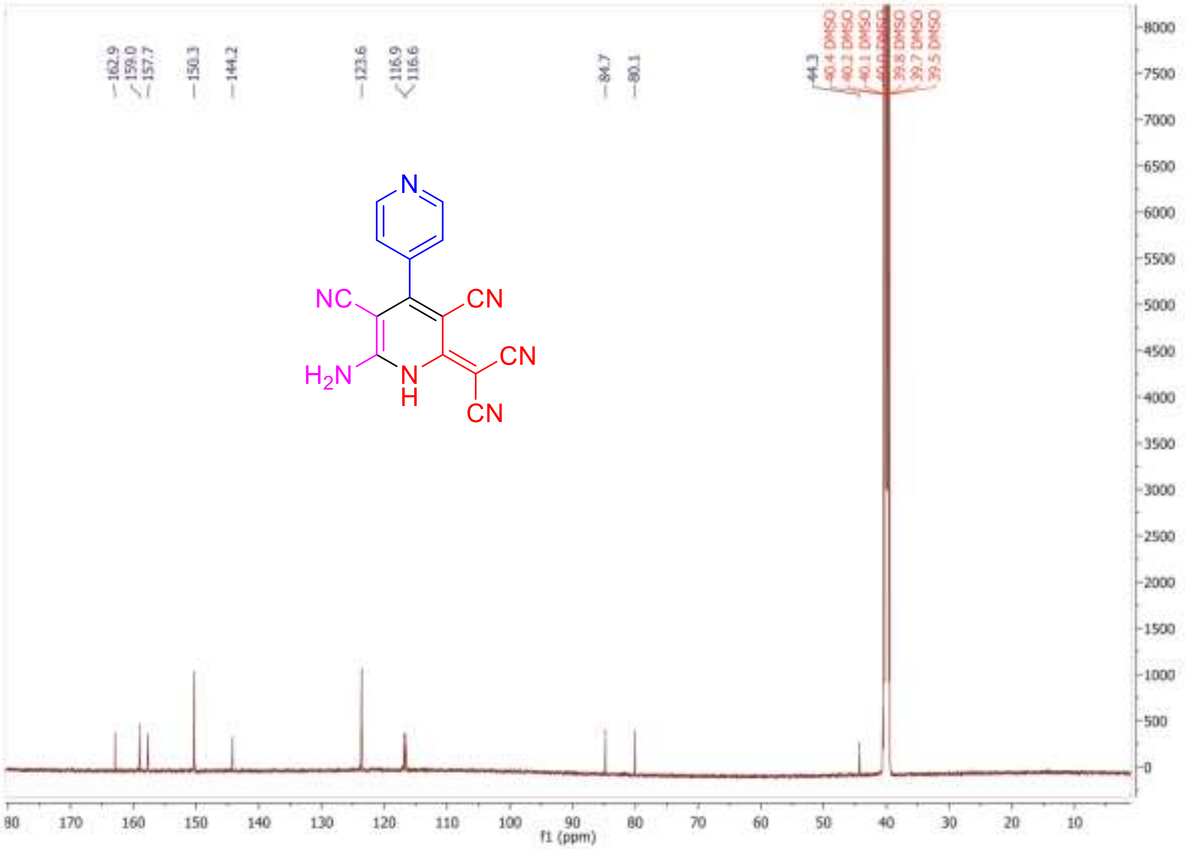

^{13}C NMR spectrum of 6-amino-2-(dicyanomethylene)-1,2-dihydro-[4,4'-bipyridine]-3,5-dicarbonitrile (i)

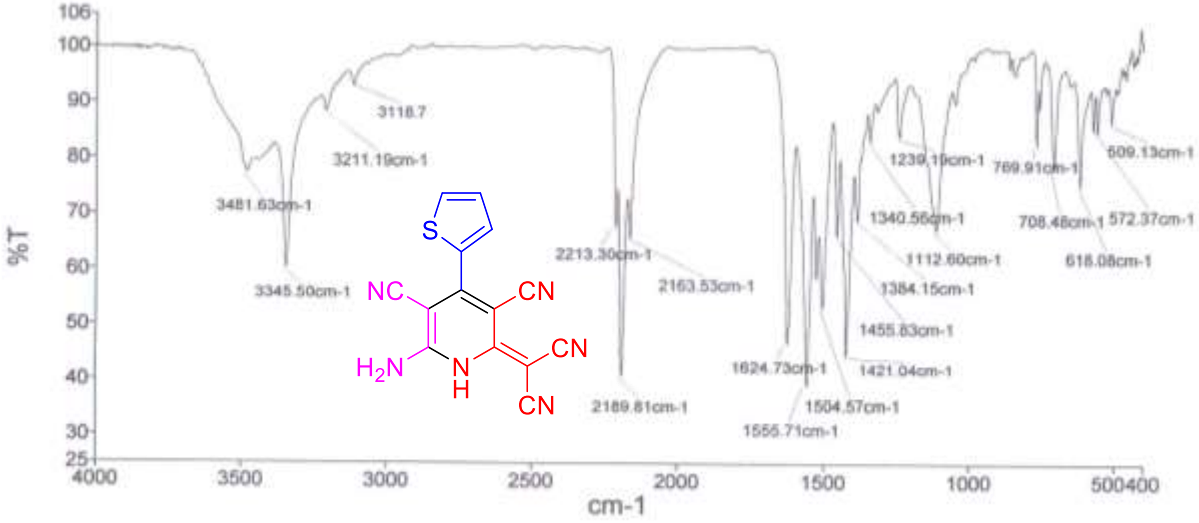

FT-IR spectrum of 6-amino-2-(dicyanomethylene)-4-(thiophen-2-yl)-1,2-dihydropyridine-3,5-dicarbonitrile (j)

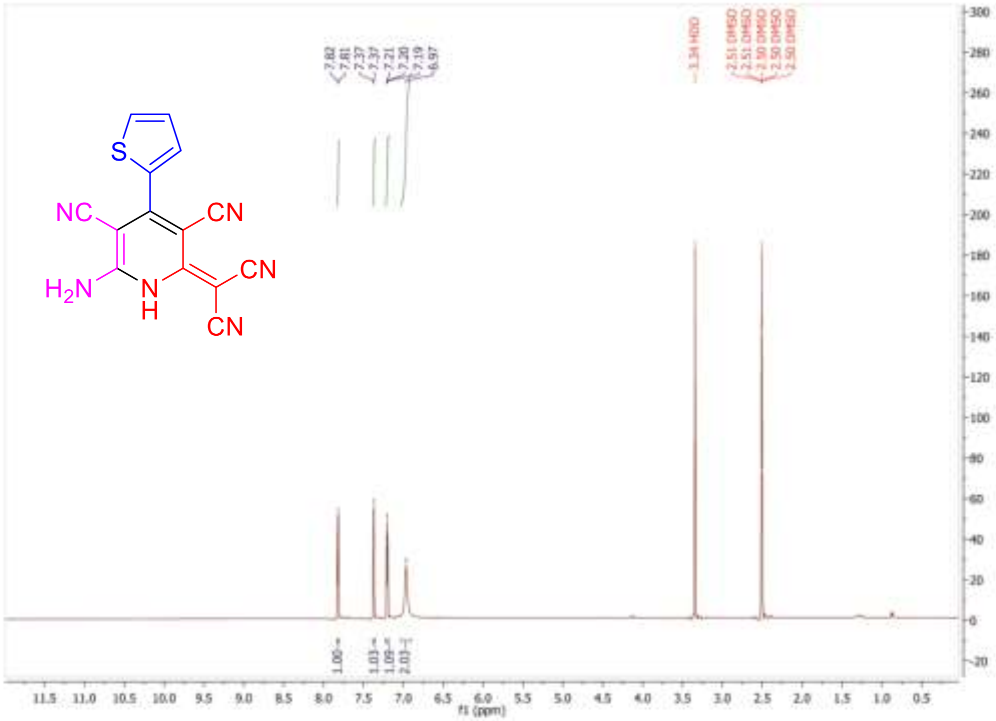

^{1}H NMR spectrum of 6-amino-2-(dicyanomethylene)-4-(thiophen-2-yl)-1,2-dihydropyridine-3,5-dicarbonitrile (j)

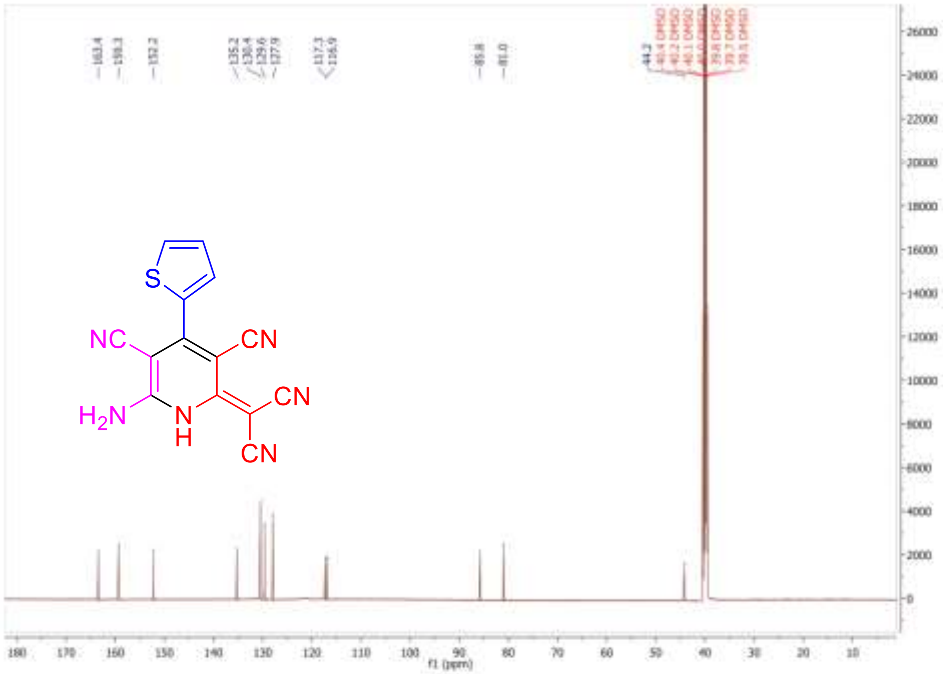

^{13}C NMR spectrum of 6-amino-2-(dicyanomethylene)-4-(thiophen-2-yl)-1,2-dihydropyridine-3,5-dicarbonitrile (j)

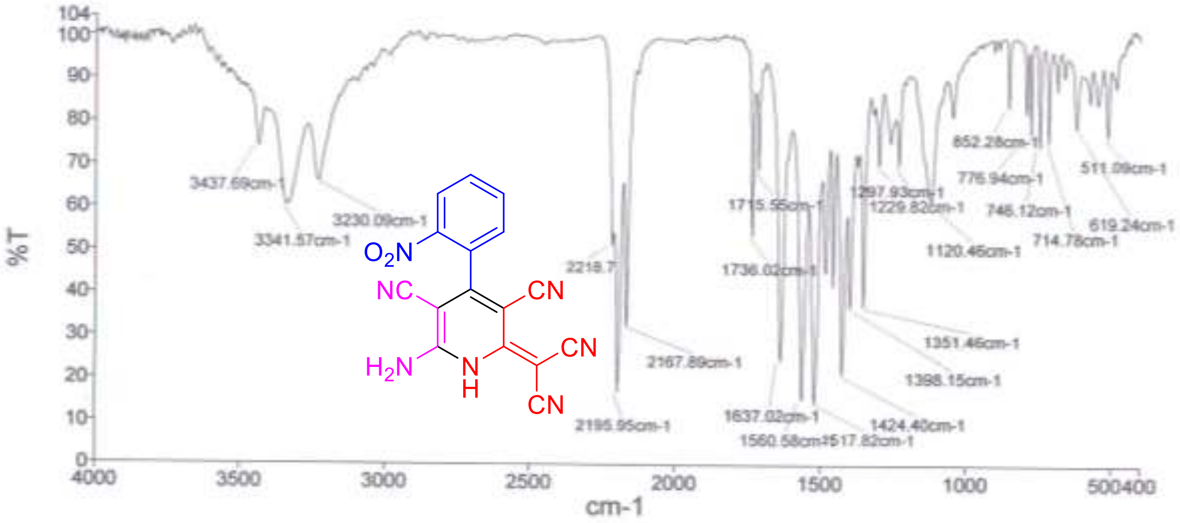

FT-IR spectrum of 6-amino-2-(dicyanomethylene)-4-(2-nitrophenyl)-1,2-dihydropyridine-3,5-dicarbonitrile (k)

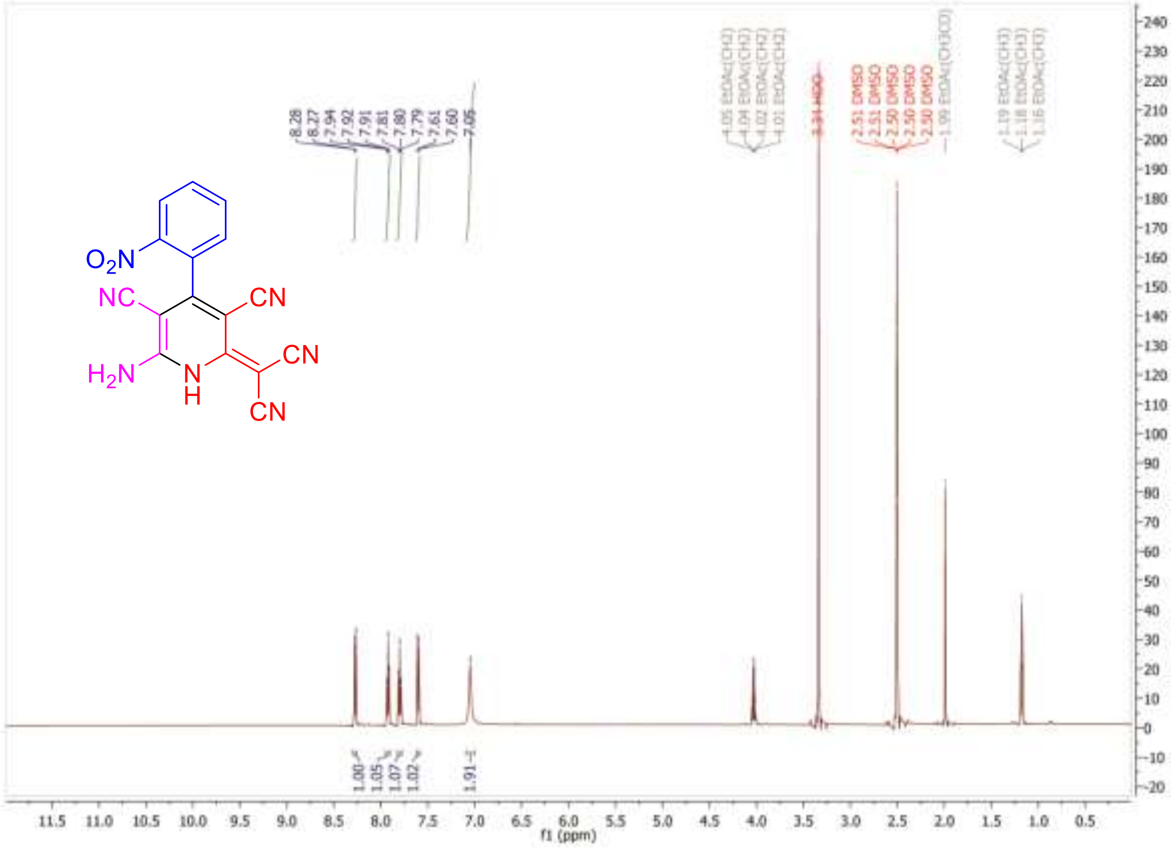

^{1}H NMR spectrum of 6-amino-2-(dicyanomethylene)-4-(2-nitrophenyl)-1,2-dihydropyridine-3,5-dicarbonitrile (k)

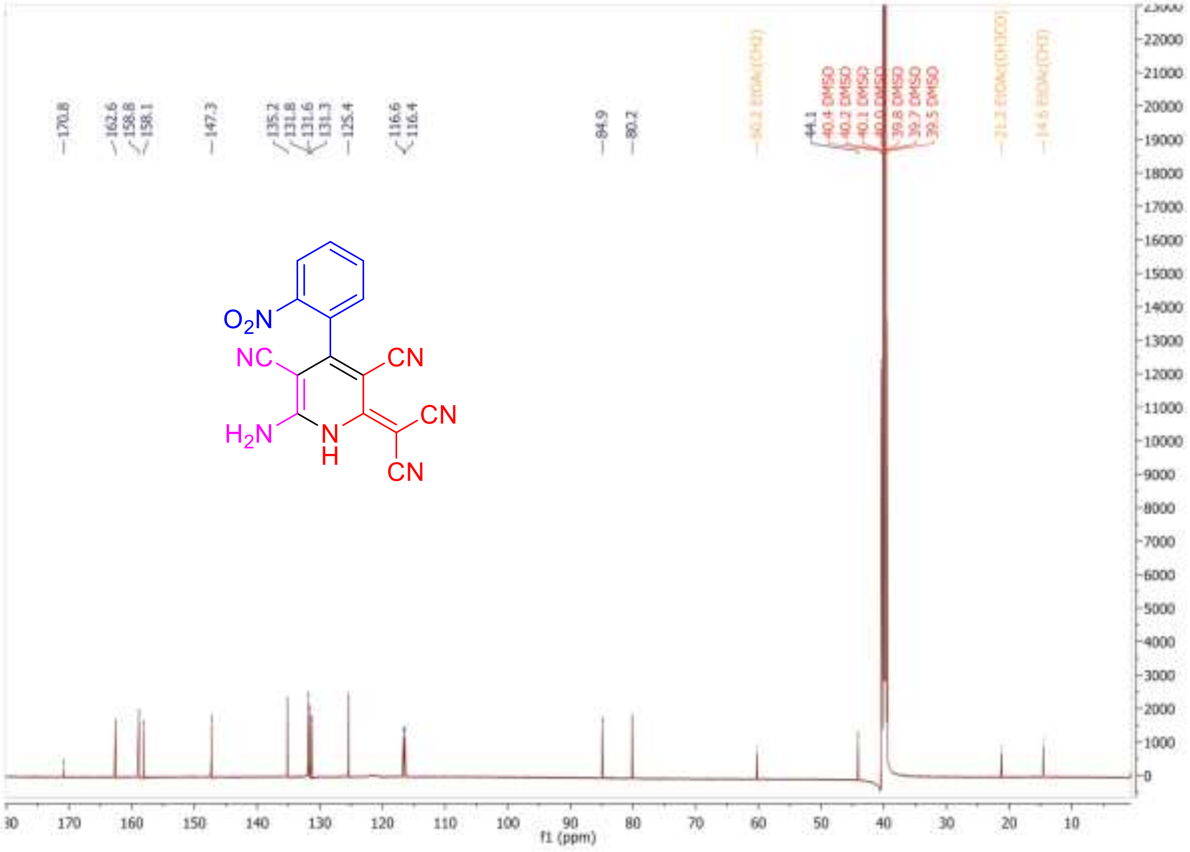

^{13}C NMR spectrum of 6-amino-2-(dicyanomethylene)-4-(2-nitrophenyl)-1,2-dihydropyridine-3,5-dicarbonitrile (k)

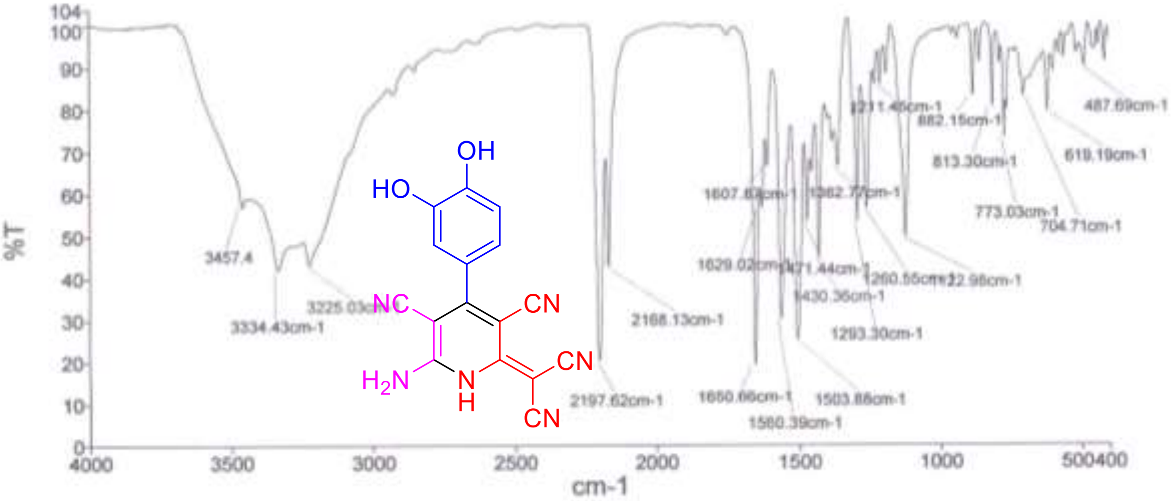

FT-IR spectrum of 6-amino-2-(dicyanomethylene)-4-(3,4-dihydroxyphenyl)-1,2-dihydropyridine-3,5-dicarbonitrile (l)

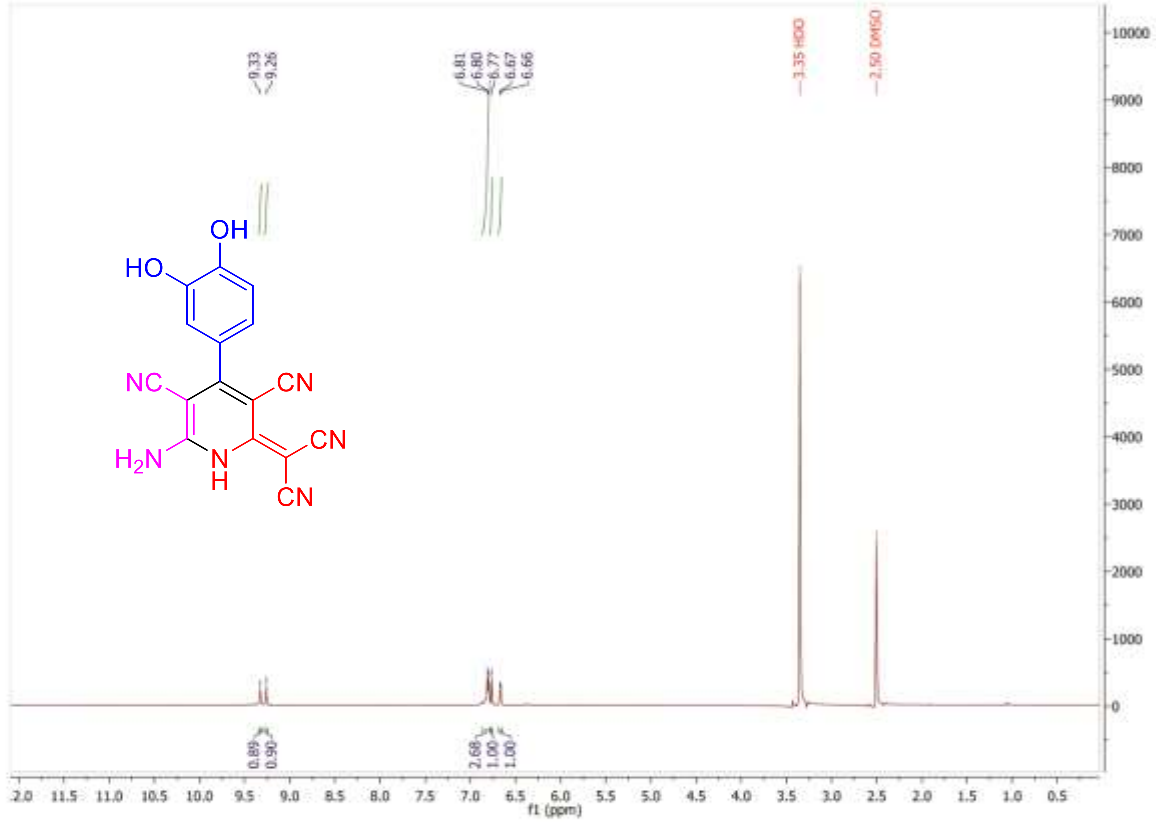

^{1}H NMR spectrum of 6-amino-2-(dicyanomethylene)-4-(3,4-dihydroxyphenyl)-1,2-dihydropyridine-3,5-dicarbonitrile (l)

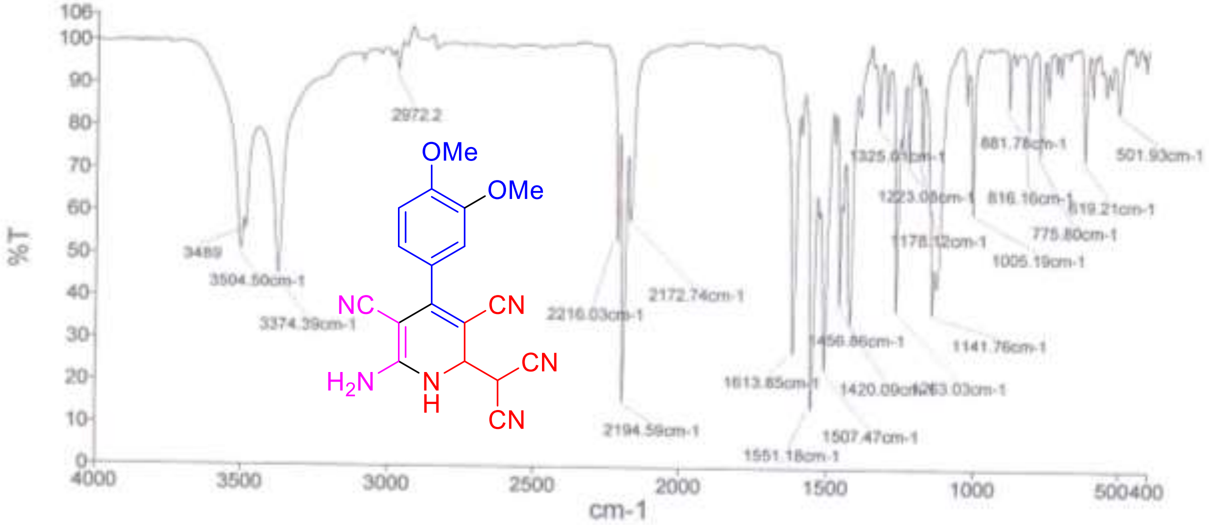

FT-IR spectrum of 6-amino-2-(dicyanomethyl)-4-(3,4-dimethoxyphenyl)-1,2-dihydropyridine-3,5-dicarbonitrile (m)

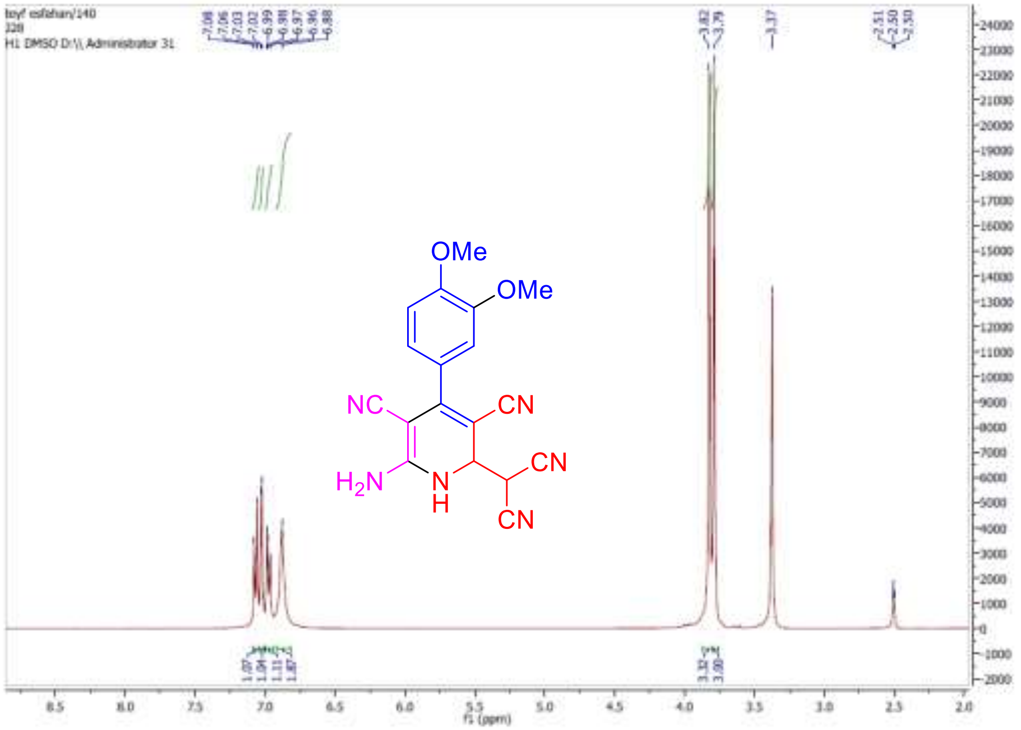

^{1}H NMR spectrum of 6-amino-2-(dicyanomethyl)-4-(3,4-dimethoxyphenyl)-1,2-dihydropyridine-3,5-dicarbonitrile (m)

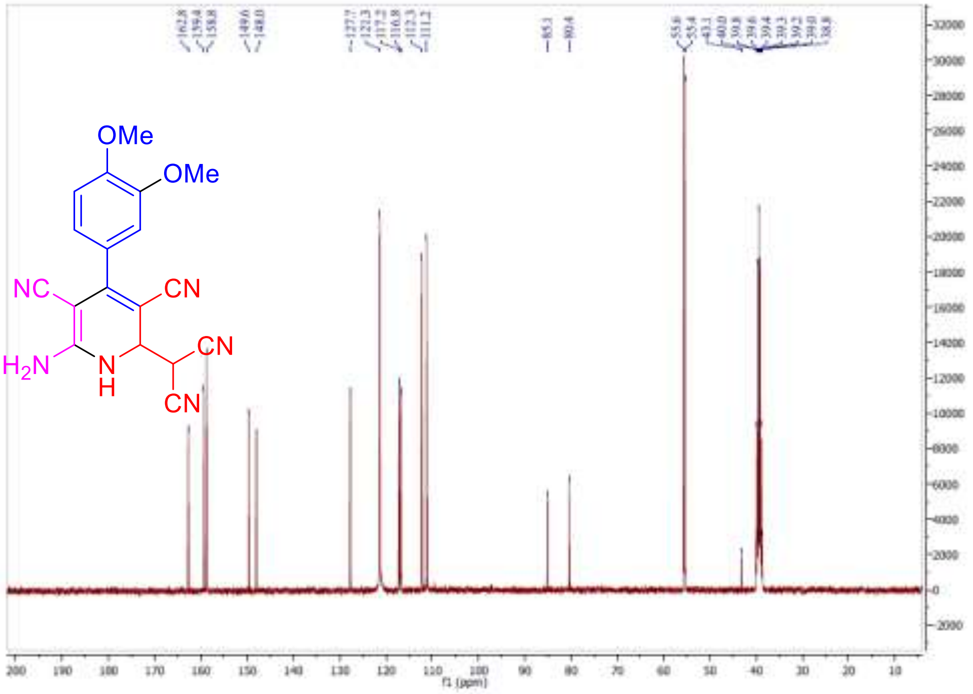

^{13}C NMR spectrum of 6-amino-2-(dicyanomethyl)-4-(3,4-dimethoxyphenyl)-1,2-dihydropyridine-3,5-dicarbonitrile (m)

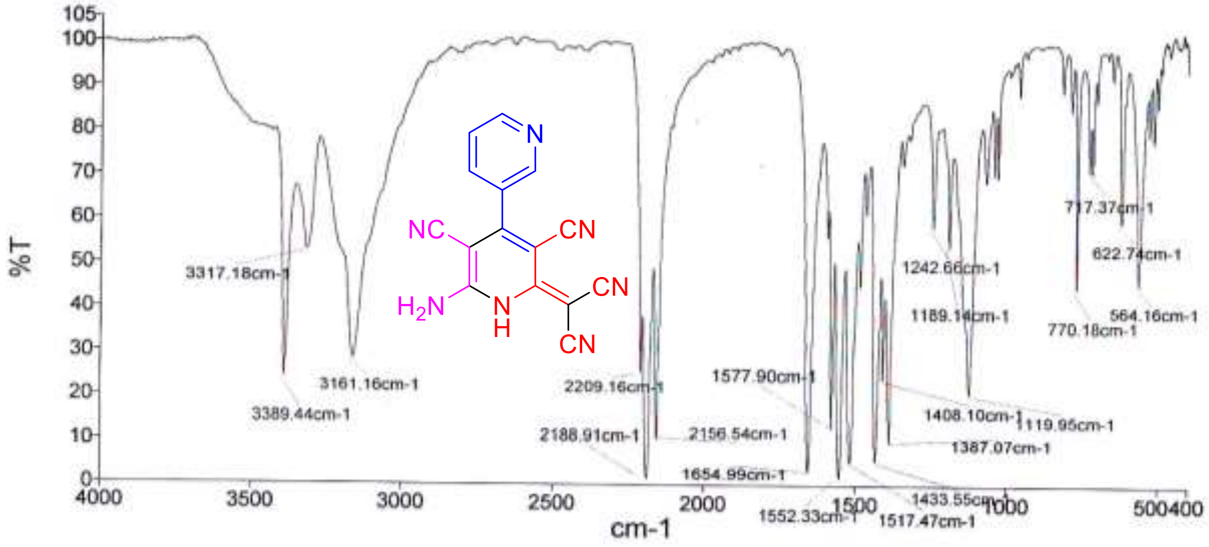

FT-IR spectrum of 6'-amino-2'-(dicyanomethylene)-1',2'-dihydro-[3,4'-bipyridine]-3',5'-dicarbonitrile (n)

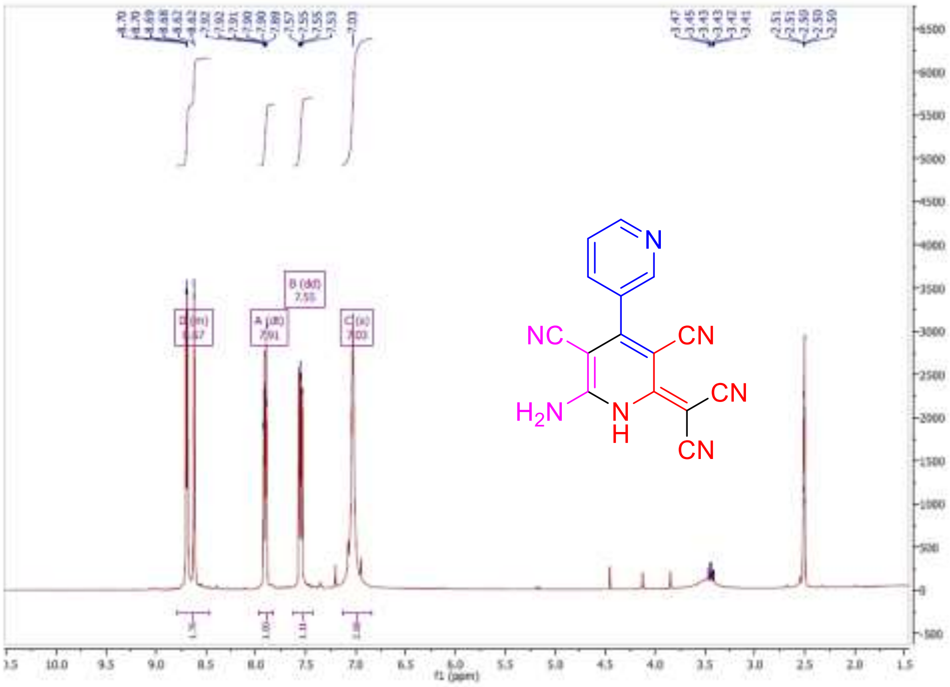

^{1}H NMR spectrum of 6'-amino-2'-(dicyanomethylene)-1',2'-dihydro-[3,4'-bipyridine]-3',5'-dicarbonitrile (n)

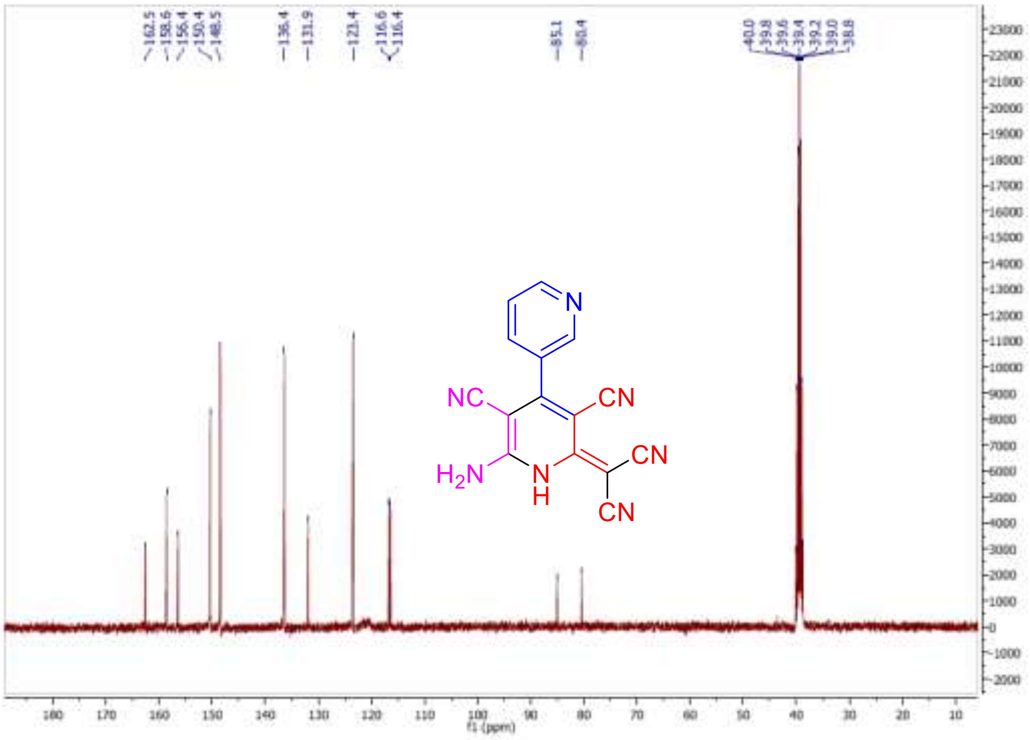

^{13}C NMR spectrum of 6'-amino-2'-(dicyanomethylene)-1',2'-dihydro-[3,4'-bipyridine]-3',5'-dicarbonitrile (n)

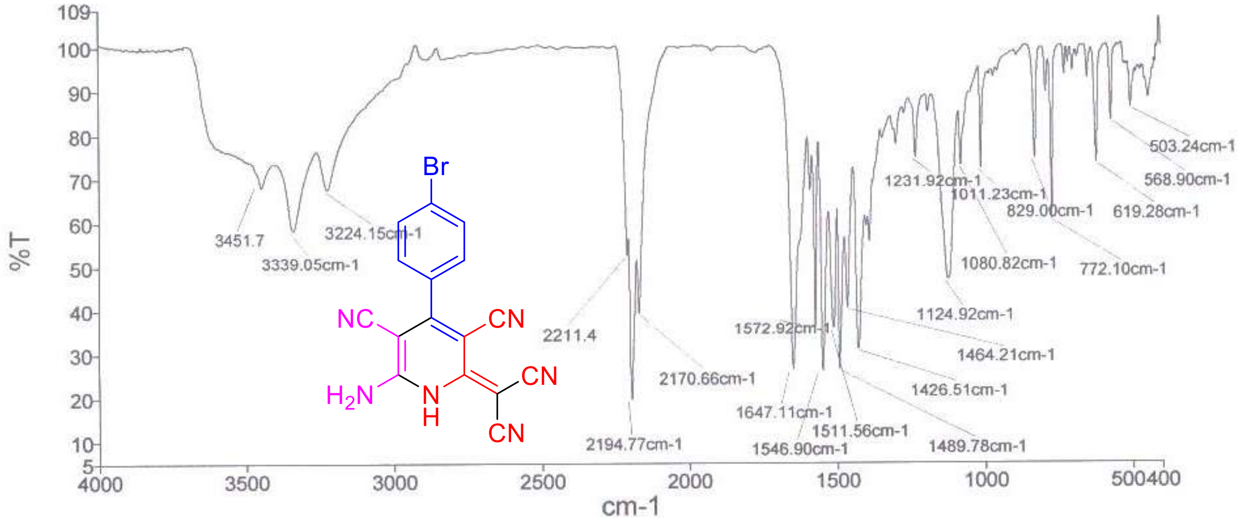

FT-IR spectrum of 6-amino-4-(4-bromophenyl)-2-(dicyanomethylene)-1,2-dihydropyridine-3,5-dicarbonitrile (o)

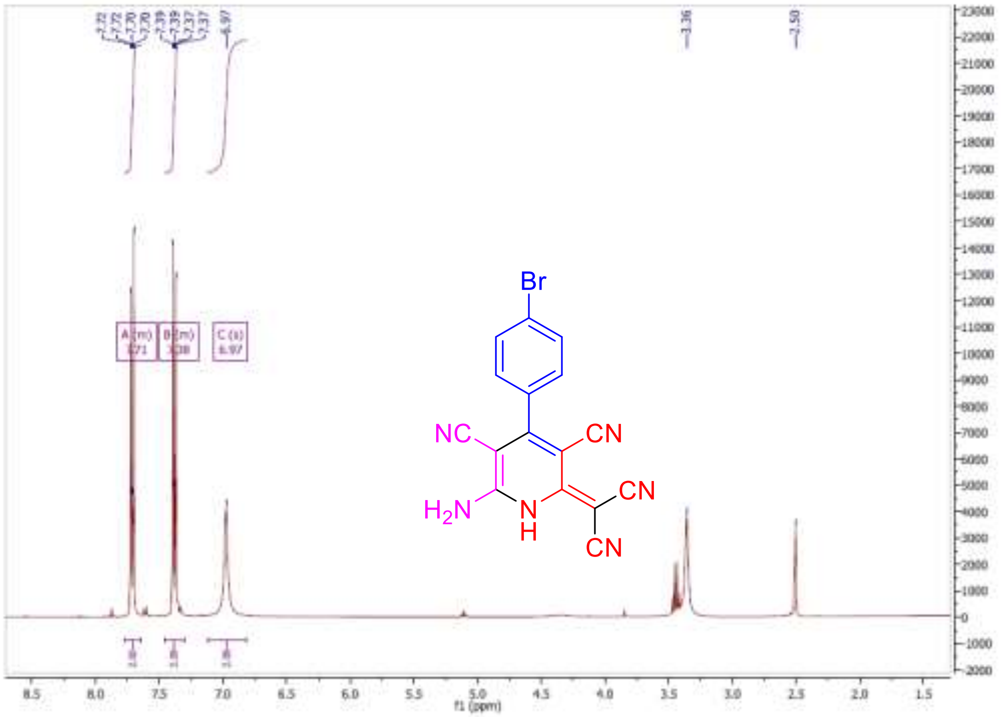

^{1}H NMR spectrum of 6-amino-4-(4-bromophenyl)-2-(dicyanomethylene)-1,2-dihydropyridine-3,5-dicarbonitrile (o)

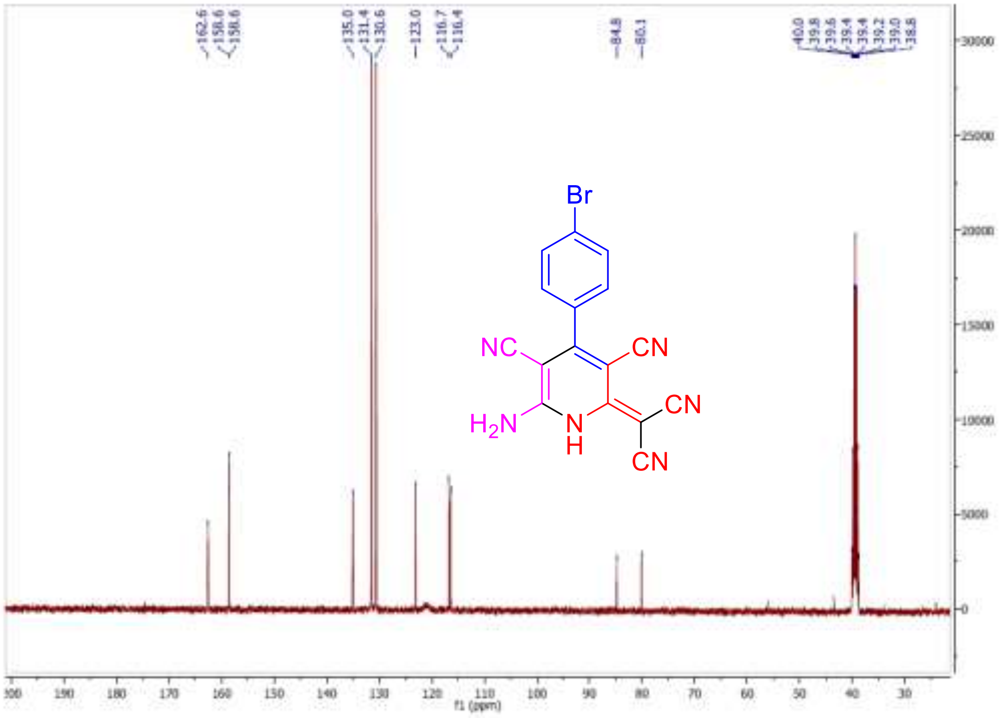

^{13}C NMR spectrum of 6-amino-4-(4-bromophenyl)-2-(dicyanomethylene)-1,2-dihydropyridine-3,5-dicarbonitrile (o)

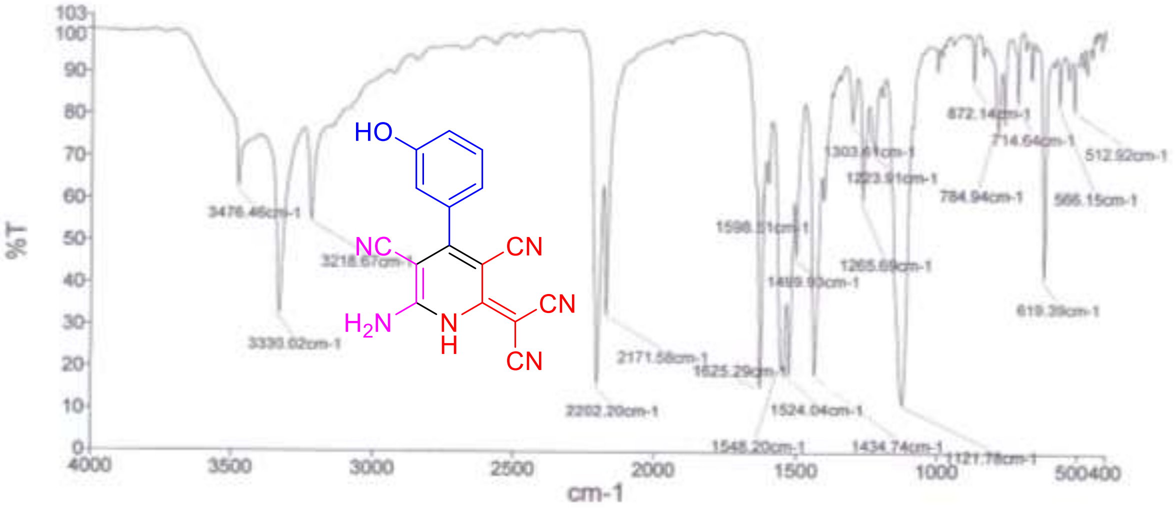

FT-IR spectrum of 6-amino-2-(dicyanomethylene)-4-(3-hydroxyphenyl)-1,2-dihydropyridine-3,5-dicarbonitrile (p)

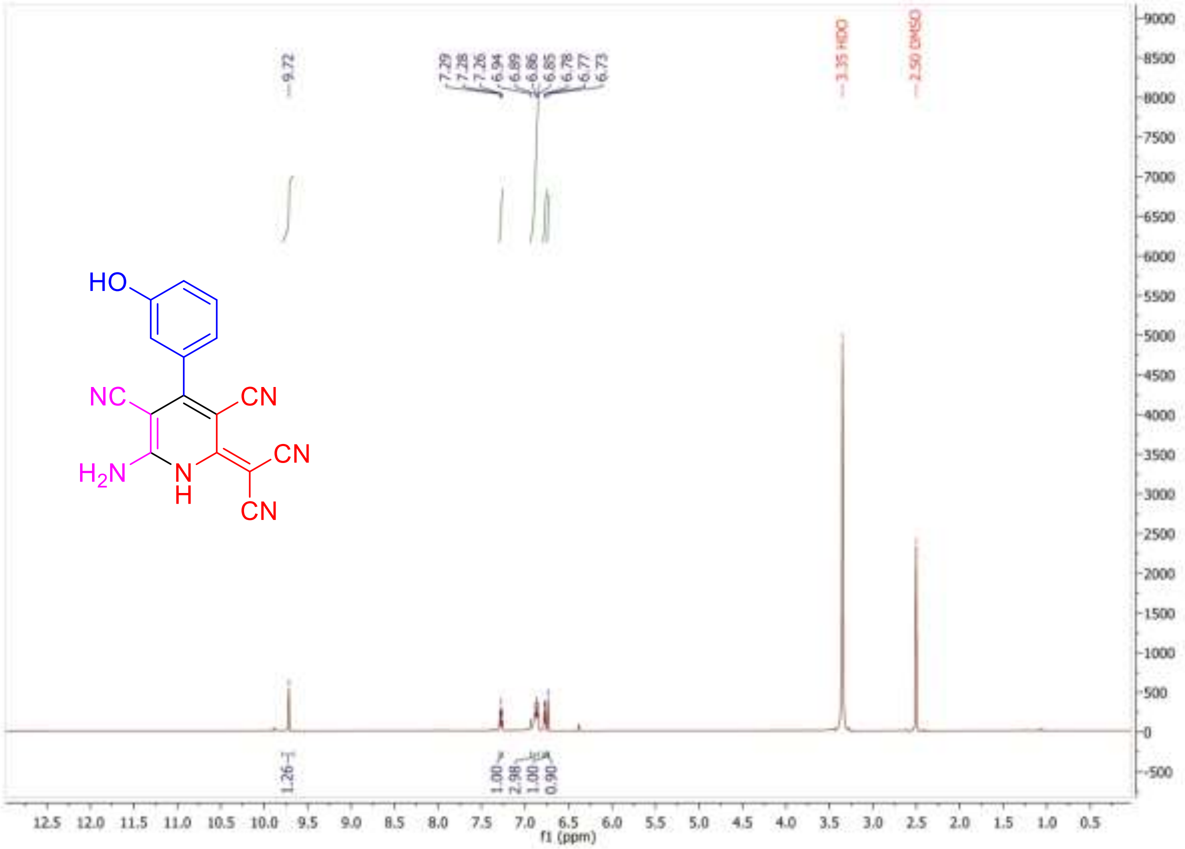

^{1}H NMR spectrum of 6-amino-2-(dicyanomethylene)-4-(3-hydroxyphenyl)-1,2-dihydropyridine-3,5-dicarbonitrile (p)

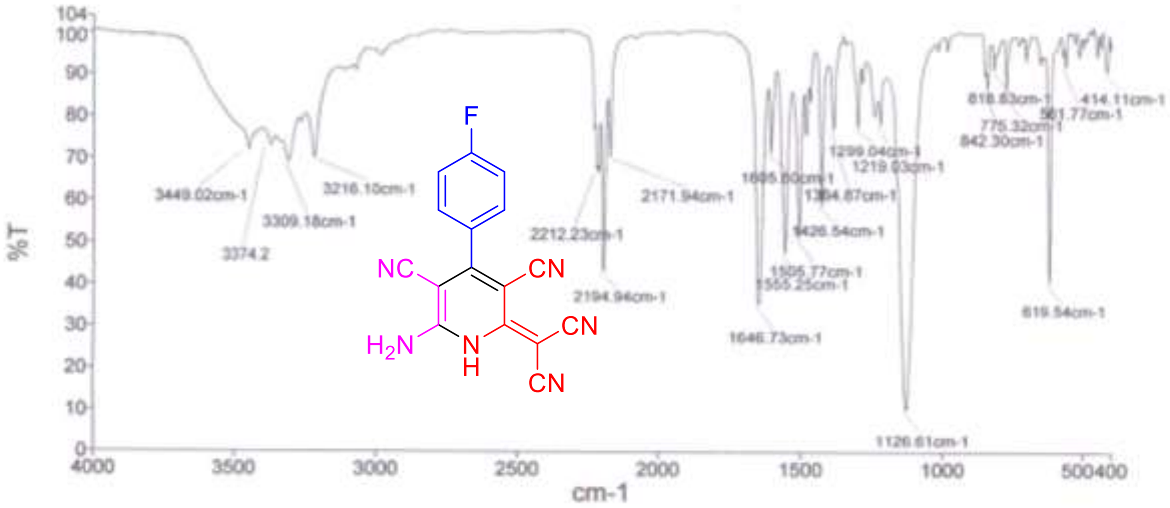

FT-IR spectrum of 6-amino-2-(dicyanomethylene)-4-(4-fluorophenyl)-1,2-dihydropyridine-3,5-dicarbonitrile (q)

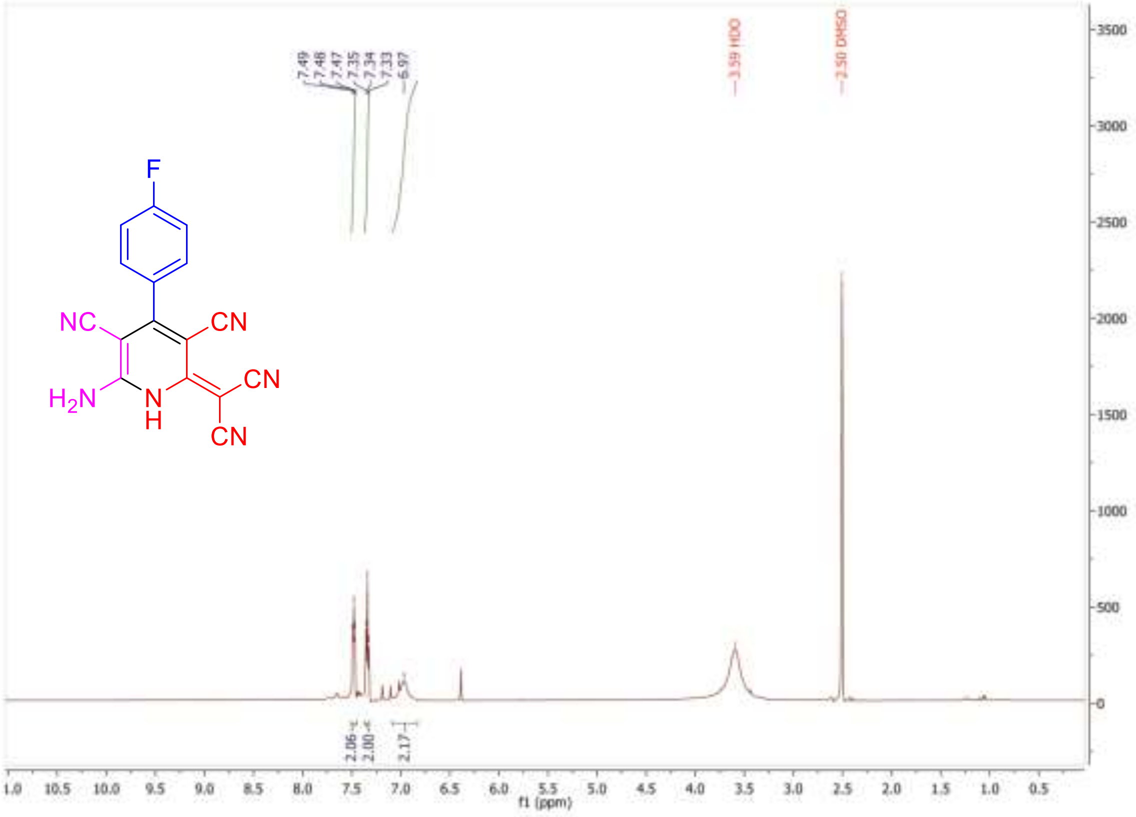

^{1}H NMR spectrum of 6-amino-2-(dicyanomethylene)-4-(4-fluorophenyl)-1,2-dihydropyridine-3,5-dicarbonitrile (q)

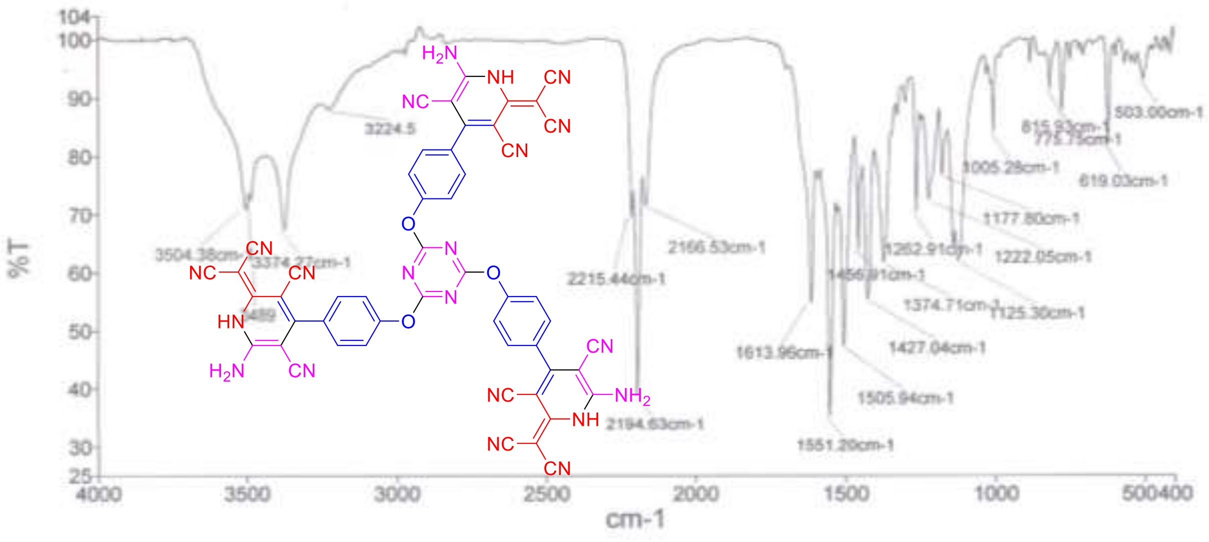

FT-IR spectrum of 4,4',4''-(((1,3,5-triazine-2,4,6-triyl)tris(oxy))tris(benzene-4,1-diyl))tris(6-amino-2-(dicyanomethylene)-1,2-dihydropyridine-3,5-dicarbonitrile) (r)

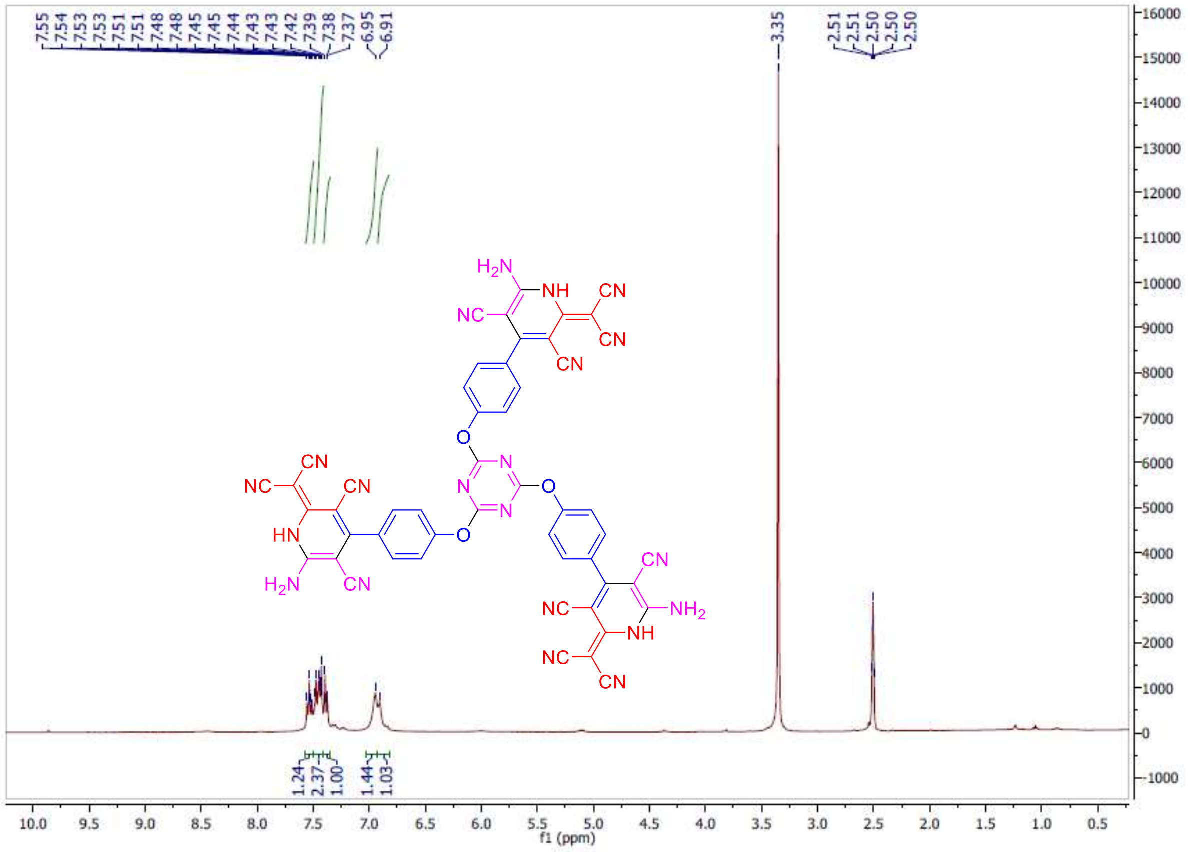

^{1}H NMR spectrum of 4,4',4''-(((1,3,5-triazine-2,4,6-triyl)tris(oxy))tris(benzene-4,1-diyl))tris(6-amino-2-(dicyanomethylene)-1,2-dihydropyridine-3,5-dicarbonitrile) (r)

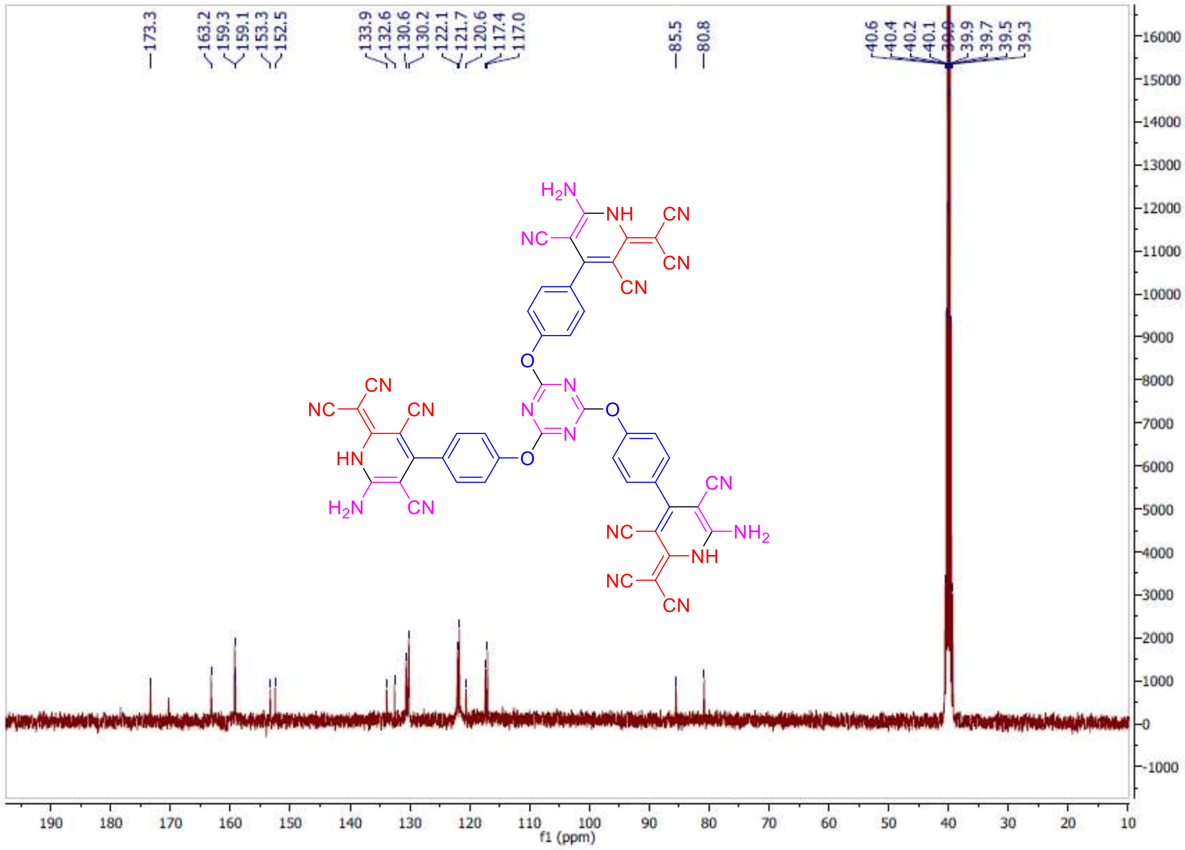

^{13}C NMR spectrum of 4,4',4''-(((1,3,5-triazine-2,4,6-triyl)tris(oxy))tris(benzene-4,1-diyl))tris(6-amino-2-(dicyanomethylene)-1,2-dihydropyridine-3,5-dicarbonitrile) (r)

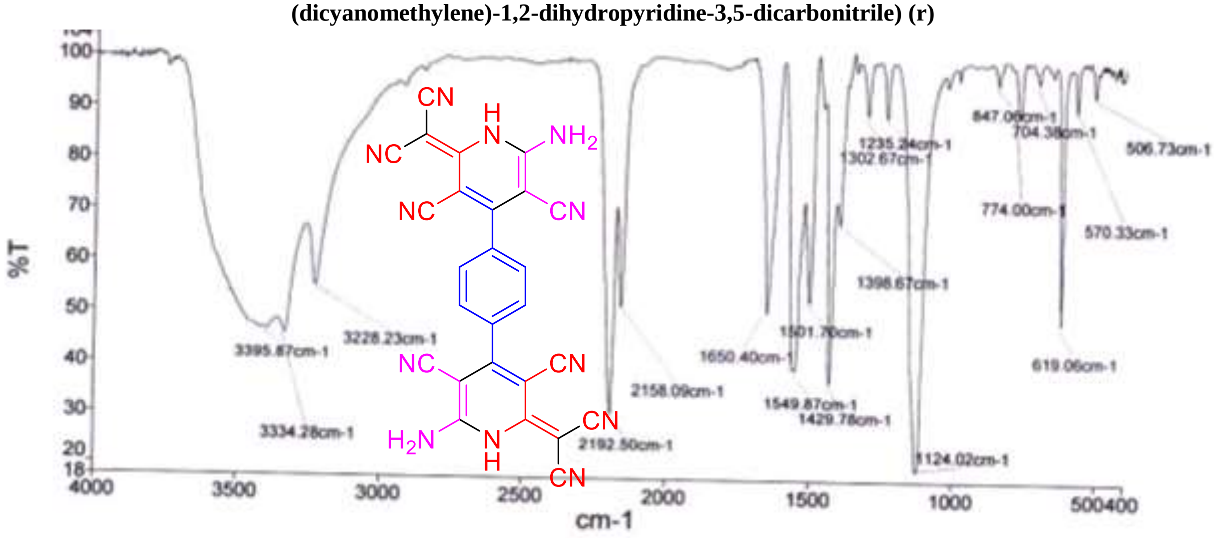

FT-IR spectrum of 4,4'-(1,4-phenylene)bis(6-amino-2-(dicyanomethylene)-1,2-dihydropyridine-3,5-dicarbonitrile) (s)

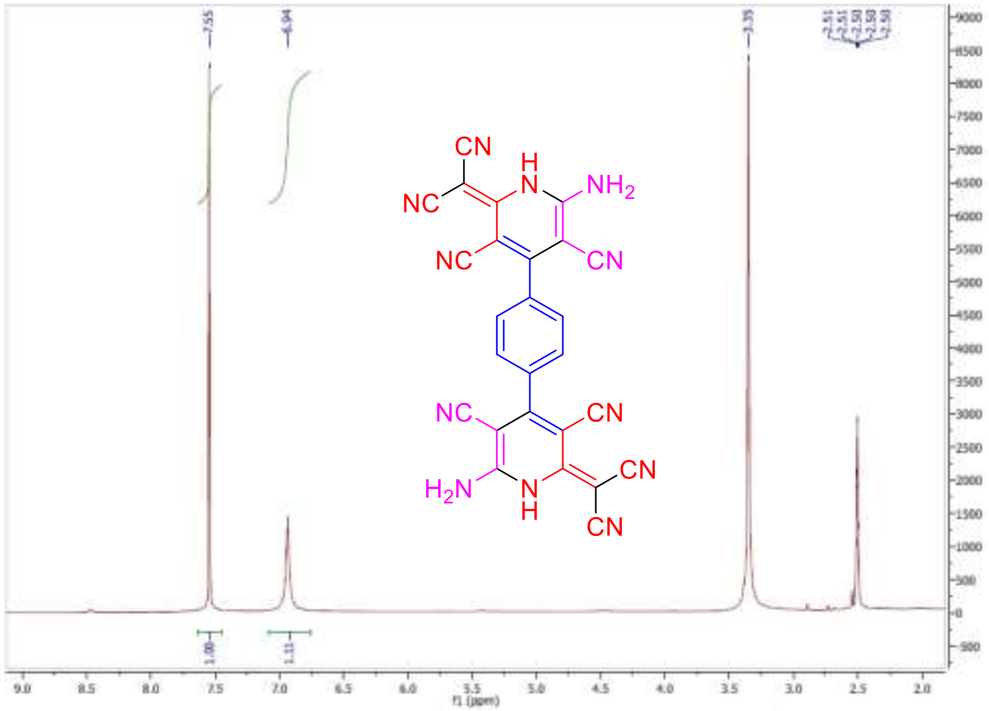

^{1}H NMR spectrum of 4,4'-(1,4-phenylene)bis(6-amino-2-(dicyanomethylene)-1,2-dihydropyridine-3,5-dicarbonitrile) (s)**

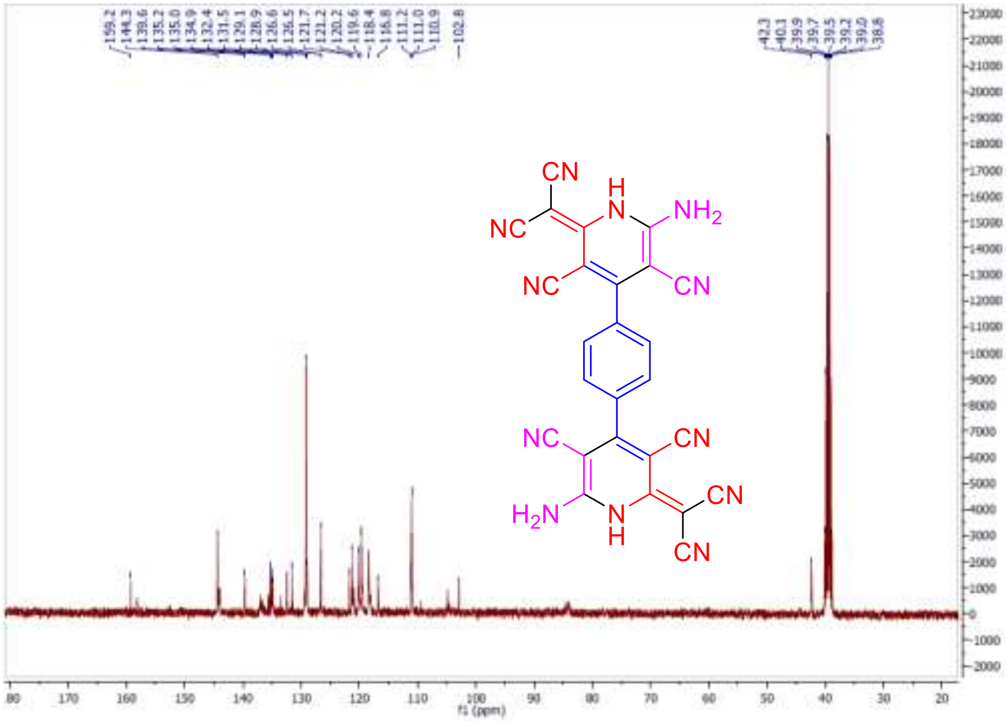

^{13}C NMR spectrum of 4,4'-(1,4-phenylene)bis(6-amino-2-(dicyanomethylene)-1,2-dihydropyridine-3,5-dicarbonitrile) (s)**
